# Supplementary material for: Ecosystem-based fisheries management forestalls climate-driven collapse
Source: Nat Commun. 2020 Sep 11;11:4579. doi: 10.1038/s41467-020-18300-3 (PMC7486947; doi:10.1038/s41467-020-18300-3)
Supplement: Supplementary file 1 — Supplementary Information [file 41467_2020_18300_MOESM1_ESM.pdf]

Supplementary Information for
Ecosystem based fisheries management forestalls climate-driven collapse

K. Holsman, A. Haynie, A. B. Hollowed, J.C.P. Reum, K. Aydin, A. J. Hermann, W. Cheng, A. Faig, J. Ianelli, K. Kearney, A. Punt.

Correspondence to: kirstin.holsman@noaa.gov

Supplementary Figures

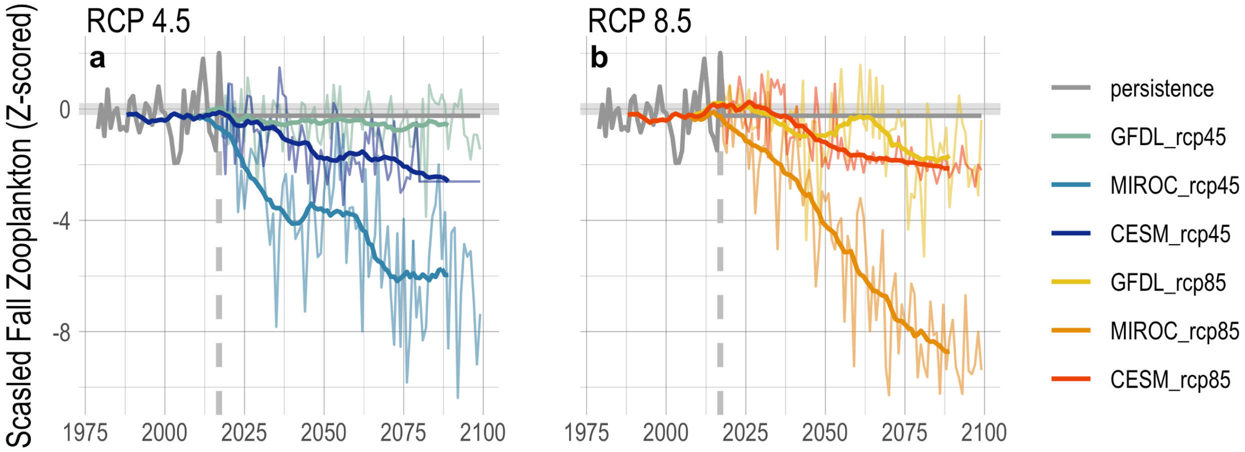

Supplementary Figure 1. Future Bering Sea fall zooplankton density. Scaled indices of fall zooplankton water column density (Z-scored) in the Bering Sea under CMIP5 Representative Concentration Pathway (RCP) 4.5 (a) and RCP 8.5 (b). Annual mean water column densities are scaled to historical mean values (thin lines) and 20 year running means (thick lines). Indices are derived from the Bering sea shelf downscaled ROMSNPZ Bering10K model and summarized for the Bering shelf during Sept-Nov of each simulation year. Persistence is based on 2006-2017 average conditions from the downscaled ROMSNPZ hindcast (gray line). Vertical dashed lines represent the start of the projection period (2018). Projections include three global climate models; the Geofluid Dynamics Lab Earth System Model (GFDL ESM2M), the MIROC-ESM, and the National Center for Atmospheric Research Community Earth System Model (CESM1).

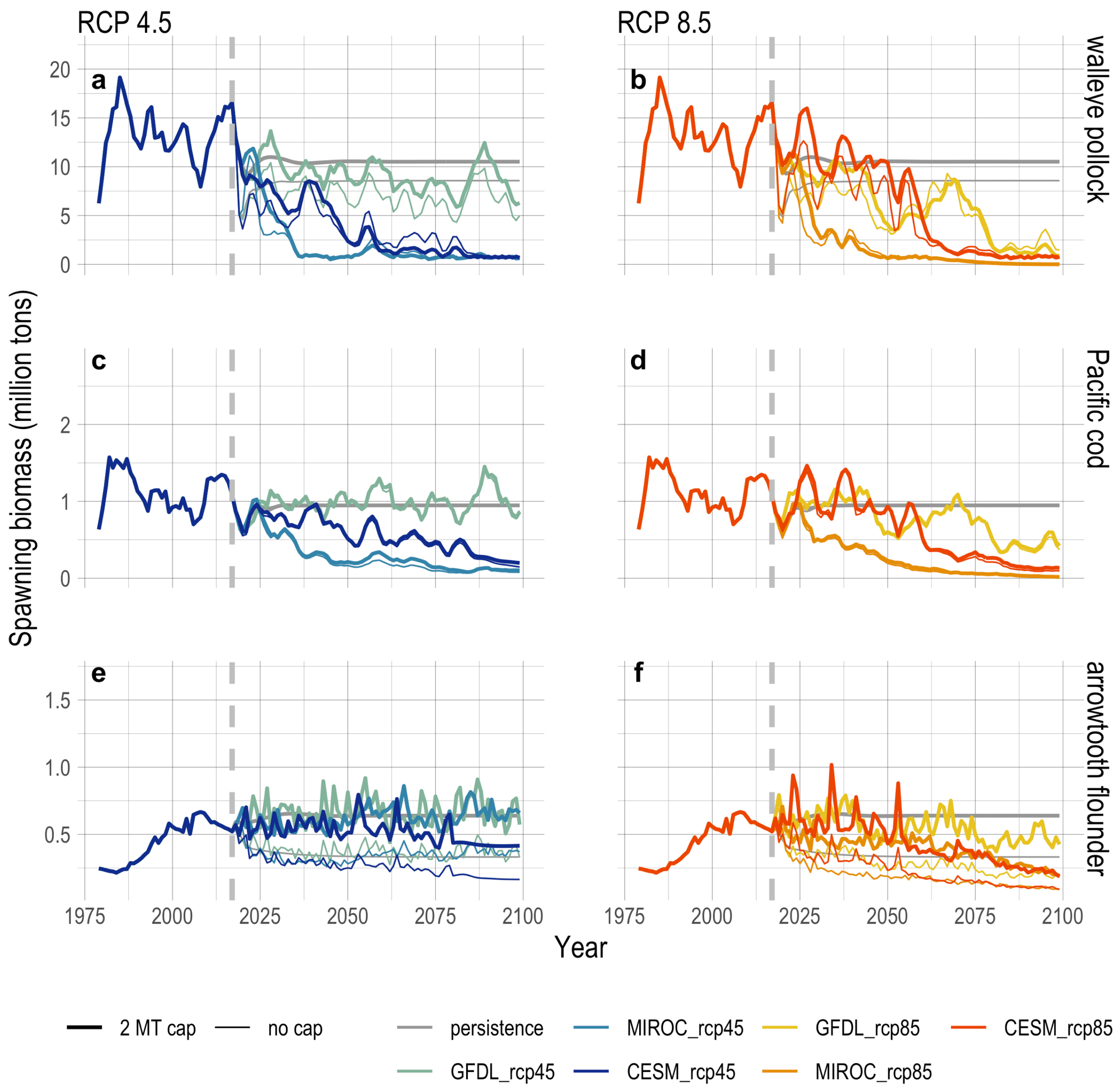

Supplementary Figure 2. Fished spawning stock biomass under each future scenario. Spawning biomass of pollock (a, b), Pacific cod (c, d), and arrowtooth flounder (e, f) under future climate change scenarios: moderate mitigation (RCP 4.5; left column), high baseline emissions (RCP 8.5; right column), and a persistence baseline climatology (solid gray lines in each panel). Lines represent management scenarios when catch is equal to the annual harvest limit (ABC_y) based on a sloping harvest control rule (i.e., no cap; thin lines) and when catch is equal to allocation using the same ABC_y in combination with a 2 MT ecosystem cap on groundfish harvest (i.e., 2 MT cap; thick lines). Vertical dashed lines represent the start of the projection period (2018-2100). For reference, the same scenario is included under the climate persistence scenario (gray lines).

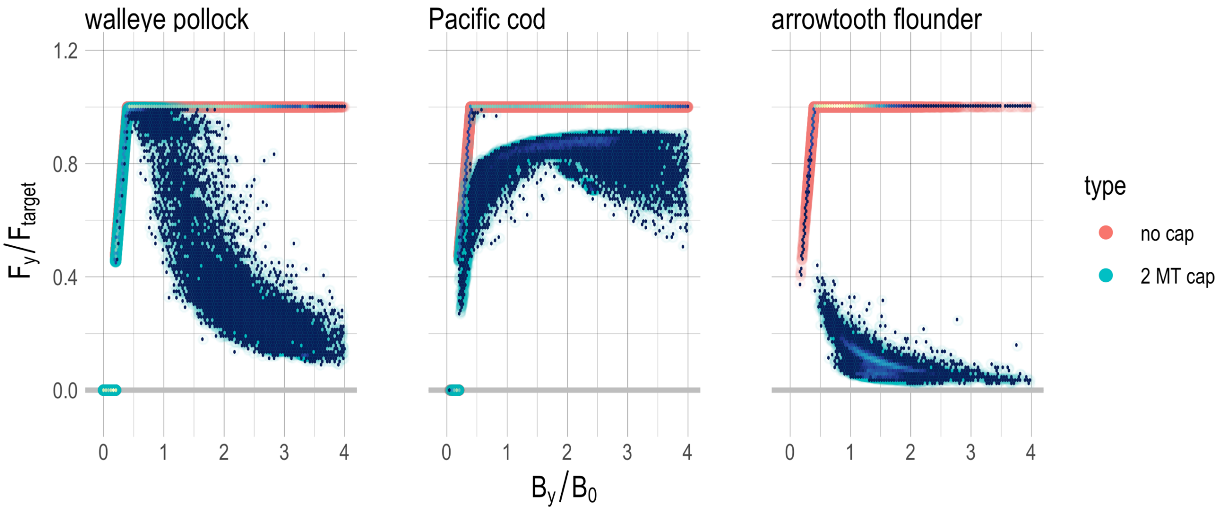

Supplementary Figure 3. Effective harvest rate F_y under the no cap and 2 MT cap scenarios. Ratio of effective F_y to F_{target} as a function of the ratio of biomass to unfished biomass reference points (B_y/B_0) for each pollock (left), Pacific cod (middle), and arrowtooth flounder (right). Scenarios without the cap (orange; follow the ABC harvest control rule exactly) and scenarios with the 2 MT cap (scatter, teal).

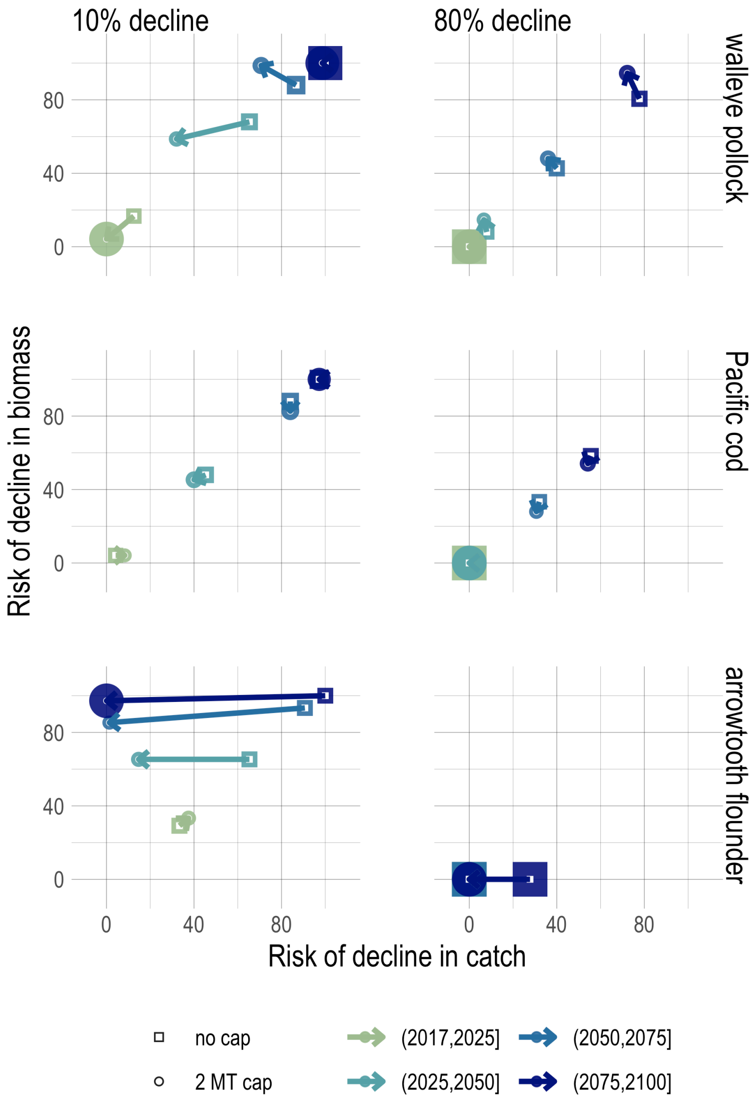

Supplementary Figure 4. Specific-specific risk of decline in catch and biomass. Species specific directional change in risk of decline under climate change (RCP 8.5 vs persistence scenario) in pollock catch or biomass with use of the EBM 2 MT cap relative to scenarios with no cap. Risk of decline (-10%; left column) and severe decline (-80%; right column) in catch or biomass for pollock (top row), Pacific cod (middle row), and arrowtooth flounder (bottom row), during four time periods for scenarios without the cap (squares) and those with the 2 MT cap (open circles). Length of arrows indicate the magnitude of change in risk between the no cap and 2 MT cap scenarios; arrows pointing towards the lower left corner indicate a decrease in risk under the 2 MT cap management strategy.

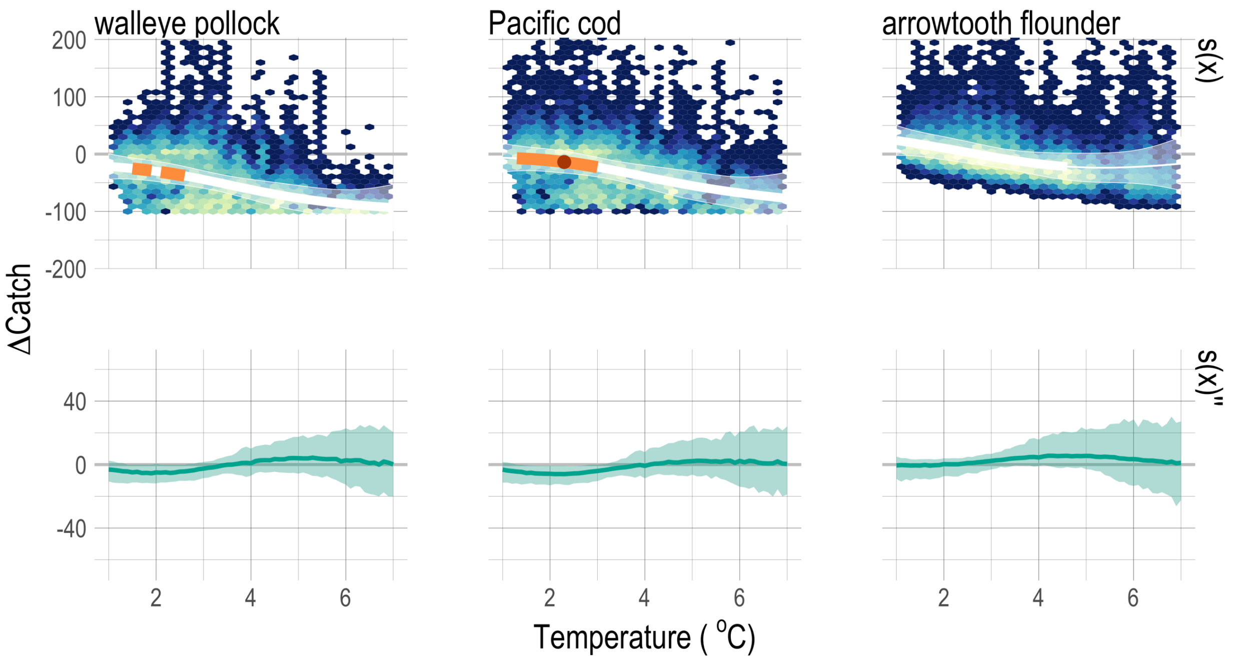

Supplementary Figure 5. Threshold analysis first and second derivatives. Threshold analyses for proportional change in catch (ΔCatch) of walleye pollock (left column), Pacific cod (middle column), arrowtooth flounder (right column) as a function of survey-replicated summer bottom temperature (°C). Top panels: solid lines represent the mean smoothing function (s(x)); shading indicates the 2.5% and 97.5% quantiles from 1000 bootstrap replicates. The thick white and orange lines indicate areas where the 95% CI of the first derivative (s(x)') of the smoothing functions do not include zero; orange bar indicates indicate where the 95% CI of the second derivative (s(x)") does not overlap zero; red points indicates the best estimate of the tipping point (i.e., s(x)" is most different from zero). Second derivatives (s"(x); bottom row) of the smoothing function (s(x)) were used to identify threshold ranges (orange band) and tipping points (red circles) for each species.

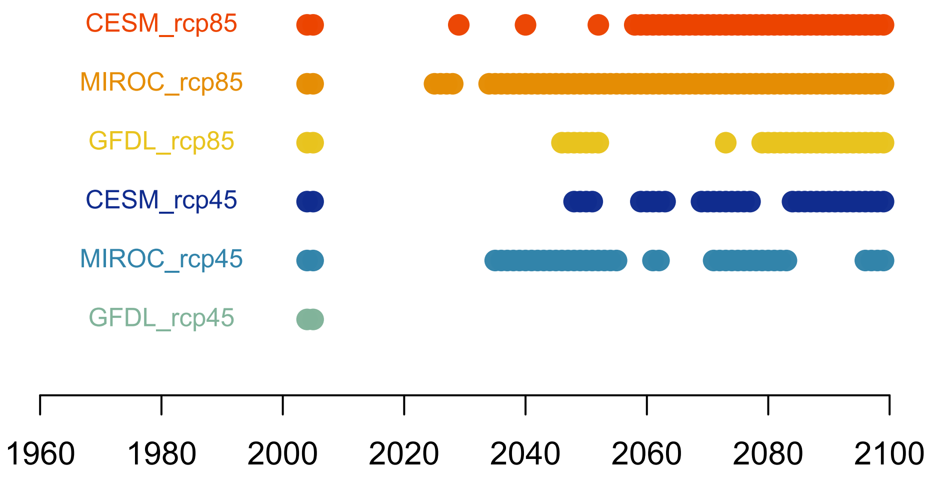

Supplementary Figure 6. Hindcast and projection years where Bering Sea bottom temperature (°C) in five consecutive years exceeds the 2.1 °C threshold. Survey replicated bottom temperature from the ROMSNPZ Bering10K downscaled projections of three global climate models; the Geofluid Dynamics Lab Earth System Model (GFDL ESM2M), the MIROC-ESM, and the National Center for Atmospheric Research Community Earth System Model (CESM1) under two representative concentration pathways; high-baseline emissions (RCP 8.5) and moderate carbon mitigation (RCP 4.5).

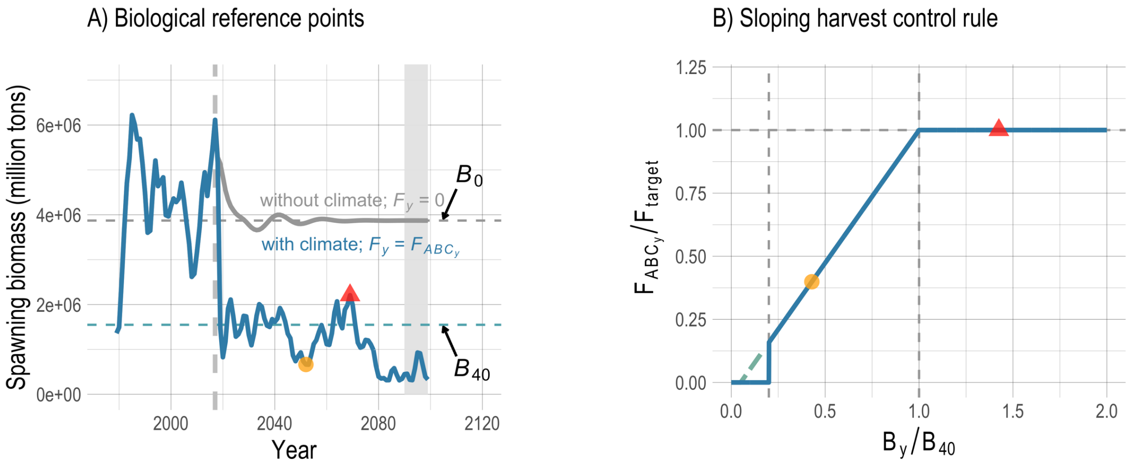

Supplementary Figure 7. Multispecies biological reference points (left panel) and sloping harvest control rule (right panel) used to project the model under management scenarios. The climate-native unfished biomass (gray line) reference point B_0 in years 2095-2099 under the persistence scenario was used to derive F_{target}, i.e., the harvest rate that results in mean spawning biomass in 2095-2099 equal to 40% of B_0 (i.e., B_{40} dashed line). Climate informed B_y (blue line left panel) was then used to adjust $F_{\text{ABC,y}}$ lower than F_{target} if $B_y <$ 40% of B_0 (e.g., yellow circle) using the North Pacific Marine Fisheries Council Tier 3 sloping harvest control rule with an ecosystem cutoff at 20% of B_0 (right panel); $F_{\text{ABC,y}} = F_{\text{target}}$ when $B_y \geq$ 40% of B_0 (e.g., red triangle).

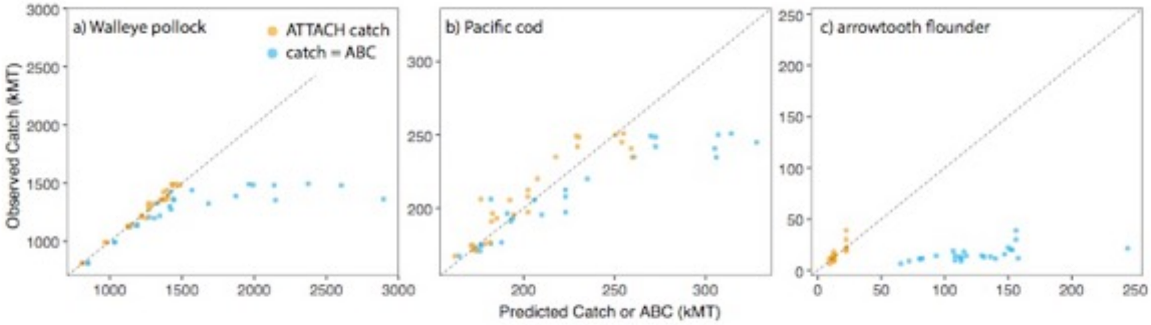

Supplementary Figure 8. Comparative performance of ATTACH to methods to those that assume catch is exactly equal to ABC. ATTACH model predicted catches (orange points) and catch = ABC (blue points) are plotted against observed catch from 1992 to 2017 for arrowtooth flounder (a), Pacific cod (b) and walleye pollock (c). The dashed line represents a 1:1 correlation. Figure modified with permission from the ATTACH model (http://doi.org/10.5281/zenodo.3966545).

Supplementary Table 1. Species-specific mean relative risk of decline in catch.

	Decline			Severe decline			Collapse		
	Risk of 10% decline in catch			Risk of 50% decline in catch			Risk of 80% decline in catch		
	**No Cap**	**2 MT cap**	**Δ risk**	**No Cap**	**2 MT cap**	**Δ risk**	**No Cap**	**2 MT cap**	**Δ risk**
**a) RCP 4.5**									
walleye pollock									
(2017,2025]	29.2 (10.2)	0 (0)	-29.2	0 (0.4)	0 (0)	0.0	0 (0)	0 (0)	0
(2025,2050]	78.7 (4.3)	41.3 (10.3)	-37.3	41.3 (5.1)	21.3 (5.5)	-20.0	20 (1.5)	20 (3.1)	0
(2050,2075]	90.7 (3.9)	66.7 (8.2)	-24 .0	65.3 (4.8)	61.3 (4.8)	-4.0	28 (6)	25.3 (7.8)	-2.7
(2075,2100]	91.7 (4.2)	68.1 (10)	-23.6	68.1 (3.2)	66.7 (3)	-1.4	58.3 (4.1)	59.7 (3.7)	1.4
Pacific cod									
(2017,2025]	12.5 (8.4)	12.5 (6.6)	0	0 (0)	0 (0)	0	0 (0)	0 (0)	0
(2025,2050]	58.7 (6.5)	57.3 (5.6)	-1.3	18.7 (3.4)	18.7 (2.9)	0	5.3 (2.5)	2.7 (2.1)	-2.7
(2050,2075]	73.3 (4.5)	73.3 (4.7)	0	50.7 (5.2)	46.7 (4.6)	-4.0	25.3 (5.5)	10.7 (4.7)	-14.7
(2075,2100]	72.2 (4)	72.2 (4.7)	0	63.9 (5.7)	62.5 (5.7)	-1.4	40.3 (5.9)	33.3 (4.7)	-7.0
arrowtooth flounder									
(2017,2025]	33.3 (5.6)	16.7 (8)	-16.7	0 (0)	0 (1.5)	0	0 (0)	0 (0)	0
(2025,2050]	61.3 (12.6)	5.3 (4.2)	-56.0	0 (0.1)	0 (0)	0	0 (0)	0 (0)	0
(2050,2075]	32 (8.9)	1.3 (3.5)	-30.7	0 (0.6)	0 (0)	0	0 (0)	0 (0)	0
(2075,2100]	38.9 (7.7)	5.6 (4.9)	-33.3	16.7 (3.9)	0 (0.3)	-16.7	0 (0)	0 (0)	0
**b) RCP 8.5**									
walleye pollock									
(2017,2025]	12.5 (5.4)	0 (0)	-12.5	0 (0)	0 (0)	0	0 (0)	0 (0)	0
(2025,2050]	65.3 (5.8)	32 (9)	-33.3	34.7 (4.9)	17.3 (6.6)	-17.3	8 (5.3)	6.7 (4.3)	-1.3
(2050,2075]	86.7 (4.2)	70.7 (12.6)	-16	64 (5)	50.7 (9.8)	-13.3	40 (6.2)	36 (5.5)	-4
(2075,2100]	100 (0.6)	98.6 (2.5)	-1.4	95.8 (4.3)	94.4 (7.1)	-1.4	77.8 (9.9)	72.2 (11.1)	-5.6
Pacific cod									
(2017,2025]	4.2 (5.9)	8.3 (7.2)	4.2	0 (0)	0 (0)	0	0 (0)	0 (0)	0

(2025,2050]	45.3 (5.4)	40 (5.3)	-5.3	17.3 (5.7)	14.7 (5.8)	-2.7	0 (0.5)	0 (0)	0
(2050,2075]	84 (5.6)	84 (7.2)	0	52 (6.4)	52 (6.5)	0	32 (6.9)	30.7 (7)	-1.3
(2075,2100]	97.2 (4.4)	97.2 (4.3)	0	83.3 (10.6)	81.9 (9.5)	-1.4	55.6 (7.3)	54.2 (5.4)	-1.4
arrowtooth flounder									
(2017,2025]	33.3 (4)	37.5 (13.5)	4.2	0 (0)	0 (2.2)	0	0 (0)	0 (0)	0
(2025,2050]	65.3 (9.9)	14.7 (5.6)	-50.7	2.7 (5.3)	0 (2)	-2.7	0 (0)	0 (0)	0
(2050,2075]	90.7 (11.5)	1.3 (3.3)	-89.3	41.3 (8.6)	0 (0)	-41.3	0 (0.8)	0 (0)	0
(2075,2100]	100 (15.2)	0 (0)	-100.0	69.4 (14.8)	0 (0)	-69.4	27.8 (3.6)	0 (0)	-27.8

Species-specific mean relative risk of decline (-10%), severe decline (-50%), and collapse (-80%) in catch under climate change relative to the baseline scenario. Risk (0-100) is reported during four time periods and under RCPs 4.5 and 8.5 for projections under management strategies without the cap and those with the 2 MT cap; differences in risk between the cap and no cap policies are also reported (i.e., Δ risk; negative values indicate a reduction in risk when the 2 MT cap is in place). Values in parentheses represent standard deviations in risk across years and GCMs.
