# Supplementary material for: Ecosystem-based fisheries management forestalls climate-driven collapse
Source: Nat Commun. 2020 Sep 11;11:4579. doi: 10.1038/s41467-020-18300-3 (PMC7486947; doi:10.1038/s41467-020-18300-3)
Supplement: Supplementary file 3 — Description of Additional Supplementary Files [file 41467_2020_18300_MOESM3_ESM.docx]

Supplementary Information Guide for

Ecosystem based fisheries management forestalls climate-driven collapse

K. Holsman, A. Haynie, A. B. Hollowed, J.C.P. Reum, K. Aydin, A. J. Hermann, W. Cheng,

A. Faig, J. Ianelli, K. Kearney, A. Punt.

Correspondence to: [kirstin.holsman@noaa.gov](mailto:kirstin.holsman@noaa.gov)

Title: Supplementary_Information.pdf

Description: Supplementary Figures 1 – 8 and Supplementary Table 1 in support of the manuscript including additional information relevant to figures and results including projections of zooplankton indices, spawning biomass, effective harvest rates, risk of decline or collapse in fisheries, tipping point methodology, biological reference point methodology, and ATTACH model performance.

Title: Supplementary_Software.zip

Description: A zipped folder containing the R code repository for regenerating figures and analyses including the folder EBM_Holsman_NatComm-master, README_Holsman_EBMpaper.pdf instructions for running the code, and EBSmultispp_2019.pdf with additional information about the CEATTLE multispecies stock assessment model. Data to support the code can be accessed at the following locations <https://github.com/kholsman/EBM_Holsman_NatComm> (working version) or <http://doi.org/10.5281/zenodo.3965248> (archived version).
