# Supplementary material for: Ecosystem-based fisheries management forestalls climate-driven collapse
Source: Nat Commun. 2020 Sep 11;11:4579. doi: 10.1038/s41467-020-18300-3 (PMC7486947; doi:10.1038/s41467-020-18300-3)
Supplement: Supplementary file 4 — Supplementary Software [file 41467_2020_18300_MOESM4_ESM.zip › Supplementary_Software/EBM_Holsman_NatComm-master/Figures/tableS1.html]

Table 1

| YrBin | 10%\_NoCapRC | 10%\_CapRC | 10%\_deltaRC | 50%\_NoCapRC | 50%\_CapRC | 50%\_deltaRC | 80%\_NoCapRC | 80%\_CapRC | 80%\_deltaRC |
| --- | --- | --- | --- | --- | --- | --- | --- | --- | --- |
| **a) RCP 4.5** | | | | | | | | | |
| **walleye pollock** | | | | | | | | | |
| (2017,2025] | 29.2 (10.2) | 0 (0) | -29.17 | 0 (0.4) | 0 (0) | 0 | 0 (0) | 0 (0) | 0 |
| (2025,2050] | 78.7 (4.3) | 41.3 (10.3) | -37.34 | 41.3 (5.1) | 21.3 (5.5) | -20 | 20 (1.5) | 20 (3.1) | 0 |
| (2050,2075] | 90.7 (3.9) | 66.7 (8.2) | -24 | 65.3 (4.8) | 61.3 (4.8) | -4 | 28 (6) | 25.3 (7.8) | -2.67 |
| (2075,2100] | 91.7 (4.2) | 68.1 (10) | -23.61 | 68.1 (3.2) | 66.7 (3) | -1.39 | 58.3 (4.1) | 59.7 (3.7) | 1.38999999999999 |
| **Pacific cod** | | | | | | | | | |
| (2017,2025] | 12.5 (8.4) | 12.5 (6.6) | 0 | 0 (0) | 0 (0) | 0 | 0 (0) | 0 (0) | 0 |
| (2025,2050] | 58.7 (6.5) | 57.3 (5.6) | -1.34 | 18.7 (3.4) | 18.7 (2.9) | 0 | 5.3 (2.5) | 2.7 (2.1) | -2.66 |
| (2050,2075] | 73.3 (4.5) | 73.3 (4.7) | 0 | 50.7 (5.2) | 46.7 (4.6) | -4 | 25.3 (5.5) | 10.7 (4.7) | -14.66 |
| (2075,2100] | 72.2 (4) | 72.2 (4.7) | 0 | 63.9 (5.7) | 62.5 (5.7) | -1.39 | 40.3 (5.9) | 33.3 (4.7) | -6.95 |
| **arrowtooth flounder** | | | | | | | | | |
| (2017,2025] | 33.3 (5.6) | 16.7 (8) | -16.66 | 0 (0) | 0 (1.5) | 0 | 0 (0) | 0 (0) | 0 |
| (2025,2050] | 61.3 (12.6) | 5.3 (4.2) | -56 | 0 (0.1) | 0 (0) | 0 | 0 (0) | 0 (0) | 0 |
| (2050,2075] | 32 (8.9) | 1.3 (3.5) | -30.67 | 0 (0.6) | 0 (0) | 0 | 0 (0) | 0 (0) | 0 |
| (2075,2100] | 38.9 (7.7) | 5.6 (4.9) | -33.33 | 16.7 (3.9) | 0 (0.3) | -16.67 | 0 (0) | 0 (0) | 0 |
| **b) RCP 8.5** | | | | | | | | | |
| **walleye pollock** | | | | | | | | | |
| (2017,2025] | 12.5 (5.4) | 0 (0) | -12.5 | 0 (0) | 0 (0) | 0 | 0 (0) | 0 (0) | 0 |
| (2025,2050] | 65.3 (5.8) | 32 (9) | -33.33 | 34.7 (4.9) | 17.3 (6.6) | -17.34 | 8 (5.3) | 6.7 (4.3) | -1.33 |
| (2050,2075] | 86.7 (4.2) | 70.7 (12.6) | -16 | 64 (5) | 50.7 (9.8) | -13.33 | 40 (6.2) | 36 (5.5) | -4 |
| (2075,2100] | 100 (0.6) | 98.6 (2.5) | -1.39 | 95.8 (4.3) | 94.4 (7.1) | -1.39 | 77.8 (9.9) | 72.2 (11.1) | -5.56 |
| **Pacific cod** | | | | | | | | | |
| (2017,2025] | 4.2 (5.9) | 8.3 (7.2) | 4.16 | 0 (0) | 0 (0) | 0 | 0 (0) | 0 (0) | 0 |
| (2025,2050] | 45.3 (5.4) | 40 (5.3) | -5.33 | 17.3 (5.7) | 14.7 (5.8) | -2.66 | 0 (0.5) | 0 (0) | 0 |
| (2050,2075] | 84 (5.6) | 84 (7.2) | 0 | 52 (6.4) | 52 (6.5) | 0 | 32 (6.9) | 30.7 (7) | -1.33 |
| (2075,2100] | 97.2 (4.4) | 97.2 (4.3) | 0 | 83.3 (10.6) | 81.9 (9.5) | -1.39 | 55.6 (7.3) | 54.2 (5.4) | -1.39 |
| **arrowtooth flounder** | | | | | | | | | |
| (2017,2025] | 33.3 (4) | 37.5 (13.5) | 4.17 | 0 (0) | 0 (2.2) | 0 | 0 (0) | 0 (0) | 0 |
| (2025,2050] | 65.3 (9.9) | 14.7 (5.6) | -50.66 | 2.7 (5.3) | 0 (2) | -2.67 | 0 (0) | 0 (0) | 0 |
| (2050,2075] | 90.7 (11.5) | 1.3 (3.3) | -89.34 | 41.3 (8.6) | 0 (0) | -41.33 | 0 (0.8) | 0 (0) | 0 |
| (2075,2100] | 100 (15.2) | 0 (0) | -100 | 69.4 (14.8) | 0 (0) | -69.44 | 27.8 (3.6) | 0 (0) | -27.78 |
