# Supplementary material for: Ecosystem-based fisheries management forestalls climate-driven collapse
Source: Nat Commun. 2020 Sep 11;11:4579. doi: 10.1038/s41467-020-18300-3 (PMC7486947; doi:10.1038/s41467-020-18300-3)
Supplement: Supplementary file 4 — Supplementary Software [file 41467_2020_18300_MOESM4_ESM.zip › Supplementary_Software/EBM_Holsman_NatComm-master/README_Holsman_EBMpaper.pdf]

Code & analyses for 'Ecosystem based fisheries management forestalls climate-driven collapse'

Holsman et al. Nature Communications

Data and code are subject to change

Repo maintained by: Kirstin Holsman
Alaska Fisheries Science Center
NOAA Fisheries, Seattle WA
kirstin.holsman@noaa.gov
Last updated: Jul 30, 2020

---

1 Overview

This is an overview of the data, code, and workflow used to generate intermediate and final data for the Holsman et al. in review Nature Communications paper. Below are instructions for downloading the code and intermediate and final data and for re-generating the figures and analyses presented in the paper. Note, in all cases species 1 = walleye pollock, species 2 = Pacific cod, and species 3 = arrowtooth flounder.

1.1. Data and Figures

Intermediate data from the ACLIM models and simulations can be found in the **data/in** sub-folder in the form of `.Rdata` files. Final data from the analyses of the paper can be found in the **data/out** sub-folder (also `.Rdata` files).

Final figures and tables (including illustrator files that were used to add fish icons) can be found in the **Figures** sub-folder. These figures can be called direct or re-generated (see section below).

2 Getting started:

2.1. Download the code from the github repository:

```
# download the code:
main_nm       <- "EBM_Holsman_NatComm-master"
download_path <- path.expand("~/desktop")
main          <- file.path(download_path,main_nm)

# download the code:
dest_file     <- file.path(download_path,paste0(main_nm,".zip"))
url           <-"https://github.com/kholsman/EBM_Holsman_NatComm/archive/master.zip"
download.file(url=url, destfile=dest_file)
```

```r
# unzip the .zip file
setwd(download_path)
unzip (dest_file, exdir = "./",overwrite = T)
setwd(main)
```

2.2. Download data from figshare:

To run the analyses or create the paper figures you will now need to download the large zipped data folder here: https://figshare.com/s/81007e2dd5edee0a5a7a (Data 10.6084/m9.figshare.12568625) and copy - paste the contents (folders "in" and "out"") it in the directory: '[your local directory path]/EBM_Holsman_NatComm/data' or simply run the following script to download and place the data in the correct sub-folders:

```r
cat("The download takes a few mins (large data files)...\n")

url <-   "https://ndownloader.figshare.com/files/23756117?private_link=81007e2dd5edee0a5a7a"

dest_path  <-  file.path(main,"data.zip")
download.file(url=url, destfile=dest_path,method="libcurl")

cat("\nDownload complete...\n")

unzip (file.path(main,"data.zip"), exdir = "./",overwrite=T)
cat("\nFiles unzipped successfully...\n")
```

If you plan to use the data within the folder for purposes beyond rerunning the paper analyses and figures please contact kirstin.holsman@noaa.gov and provide the following citation for the data:

Holsman, K. K., A. Haynie, A. Hollowed, J.C.P. Reum, K. Aydin, A. J. Hermann, W. Cheng, A. Faig, J. N. Ianelli, K. Kearney, A. E. Punt,2020.The Alaska Climate Integrated Modeling study Multi-species Ecosystem Based Fisheries Management and climate change simulation and results data. DOI: 10.6084/m9.figshare.12568625

as well as the corresponding manuscript:

Holsman, K. K., A. Haynie, A. Hollowed, J.C.P. Reum, K. Aydin, A. J. Hermann, W. Cheng, A. Faig, J. N. Ianelli, K. Kearney, A. E. Punt, 2020.Ecosystem-based fisheries management forestalls climate-driven collapse, Nature Communications.

3 Regenerating analyses and figures:

Below are instructions for running the R scripts to recreate the figures, tables, results, and run the risk and threshold analyses for the paper. The scripts below require R version 3.5.3 (available at https://cran.r-project.org/bin/macosx/el-capitan/base/).

3.1. Set things up:

The first step is to run the make.R script to load the data, packages, and setup (where various options are specified). Note that the scripts depend on a number of packages that will be installed the first time through running make.R if they are not already included. *A list of those packages can be found in 'EBM_Holsman_NatComm/R/packages.R'.*

```r
    # set your local path:
    # main        <-  file.path(download_path,"EBM_Holsman_NatComm/")
    # e.g., main  <-  getwd()
    setwd(main)

    # load data, packages, setup, etc.
    source("R/make.R")
```

3.2. Regenerate plots:

To generate the figures in the paper without overwriting them run the 'make_plots.R' code.

```r
    cat(paste("\n\n update.figs  = ",update.figs,"\n"))
    cat(paste("\n\n update.outputs  = ",update.outputs,"\n"))

    # You can generate individual plots like this (some take a few mins and throw warnings):
    graphics.off()
    fig2()
    fig3() #  slow....
    fig4() #  slow....
    fig5()
    fig6()
    figS1()
    figS2() #  slow....
    figS3() #  slow....
    figS4()
    figS5()
    figS6()
    figS7()
```

Alternatively, by setting `update.figs` to `TRUE` the script will overwrite the existing figures in the `Figures` folder:

```r
    update.figs  <- TRUE
    warning("warning! this will overwrite existing figures in the Figures folder")

    source("R/sub_scripts/make_plots.R")  # this throws warnings that can be ignored
    update.figs  <- FALSE                 # return this to it's default setting
```

3.3. Regenerate analyses and final data

Analysis results are located in the `data/out` folder and loaded during `R/make.R`:

```r
   head(risk12)  # preview the risk table for "No cap" simulations
   head(risk13)  # preview the risk table for "2 MT cap" simulations
   C_thresh_12_1$thrsh_x # Temperature tipping point for pollock under "No cap" simulations
   C_thresh_12_2$thrsh_x # Temperature tipping point for p cod under "No cap" simulations
   C_thresh_12_3$thrsh_x # No tipping point was found for arrowtooth under "No cap" simulations

 tmp <- list("No Cap" = c(
   C_thresh_12_1$thrsh_x,
   C_thresh_12_2$thrsh_x,
   C_thresh_12_3$thrsh_x),
```

```
  "2 MT Cap" = c(
  C_thresh_13_1$thrsh_x,
  C_thresh_13_2$thrsh_x,
  C_thresh_13_3$thrsh_x))

# get mean and var for tipping points:
mean(as.numeric(unlist(tmp)))
sd(as.numeric(unlist(tmp)))

# double check:
threshIN     <- C_thresh_13_2
thrsh2_all   <- intersect(threshIN$signif2,threshIN$ix_pks)
df2_qnt      <- threshIN$df2_qnt
df2_qnt$tmp[(thrsh2_all[which( abs(df2_qnt$smoothed_mn[thrsh2_all])  == max(abs(df2_qnt$smoothed_mn[th
df2_qnt$tmp[(thrsh2_all[which( abs(df2_qnt$smoothed_dwn[thrsh2_all]) == max(abs(df2_qnt$smoothed_dwn[t
df2_qnt$tmp[(thrsh2_all[which( abs(df2_qnt$smoothed_up[thrsh2_all])  == max(abs(df2_qnt$smoothed_up[th
```

To re-run the paper analyses, including risk calculations and threshold/tipping points, set `update.outputs = TRUE`(this is set to `FALSE` by default) and run the `SUB_EBM_paper.R` script:

```
    update.outputs  <- TRUE
    warning("warning! Setting update.outputs to TRUE will overwrite Rdata files in the data folder")

    source("R/sub_scripts/SUB_EBM_paper.R")

    # once complete set to FALSE and reload new datafiles:
    update.outputs  <- FALSE
    source("R/make.R")
```

To recreate the Table S1 (uses kable to create table as html):

```
    # to print table set eval =T
    tbl <- tableS1()
    tbl
    save_kable(tbl,file=file.path(main,"Figures/tableS1.html"))
```

.

4 Primary and Intermediate Data sources and models

Various simulation outputs were made available for use in this analysis through the interdisciplinary Alaska Climate Integrated Modeling (ACLIM) project. An overview of the project and simulation experiments can be found in Hollowed et al. 2020.

Hollowed, A. B., K. K. Holsman, A. C. Haynie, A. J. Hermann, A. E. Punt, K. Aydin, J. N. Ianelli, S. Kasperski, W. Cheng, A. Faig, K. A. Kearney, J. C. P. Reum, P. Spencer, I. Spies, W. Stockhausen, C. S. Szuwalski, G. A. Whitehouse, and T. K. Wilderbuer. 2020. Integrated Modeling to Evaluate Climate Change Impacts on Coupled Social-Ecological Systems in Alaska .

4.1. Bering10K ROMSNPZ model

ACLIM indices used in this analysis can be viewed interactively online at: https://kholsman.shinyapps.io/aclim. The indices were produced for the ACLIM project and derived from the outputs of the Bering10K

ROMSNPZ model. Downscaled hindcasts and CMIP5 projections of oceanographic and lower trophic conditions from the Bering10K model were developed as part of the ACLIM project. An overview of these projections and the Bering10K ROMSNPZ model can be found in Hermann et al. 2019, Kearney et al. 2020, and Hollowed et al. 2020. An overview of the Bering10K ROMSNPZ model can be found here.

Citation:

Hermann, A. J., G. A. Gibson, W. Cheng, I. Ortiz, K. Aydin, M. Wang, A. B. Hollowed, and K. K. Holsman. 2019. Projected biophysical conditions of the Bering Sea to 2100 under multiple emission scenarios. ICES Journal of Marine Science.

Kearney K, Hermann A, Cheng W, Ortiz I, Aydin K (2020) A coupled pelagic-benthic-sympagic biogeochemical model for the Bering Sea: documentation and validation of the BESTNPZ model (v2019.08.23) within a high-resolution regional ocean model. Geosci Model Dev 13:597-650. DOI:10.5194/gmd-13-597-2020.

4.2. CEATTLE multispecies model

CEATTLE is a climate-enhanced multispecies stock assessment model for walleye pollock, Pacific cod, and arrowtooth flounder (Holsman et al. 2016, 2019) that has been updated annually and included as an appendix to the BSAI walleye pollock stock assessment (Ianelli et al. 2019) since 2016 as part of the Bering Sea fishery stock assessment process. As part of ACLIM CEATTLE was coupled to the ROMSNPZ model and the ATTACH model (below) to generate projections of species biomass and catch under future climate conditions in the Bering Sea. Methods for this coupling and projection simulation can be found in Holsman et al. submitted and Hollowed et al. 2020. The simulation outputs, scripts, and input data files used to generate these simulations can be found on the ACLIM-CEATTLE gitrepo and figshare sites. Details about the CEATTLE model can be found in the 2018 Multispecies assessment and 2019 Assessments.

Citation: Holsman, K. K., J. N. Ianelli, K. Aydin, and I. Spies. 2019. Multi-species supplement: 2019 Climate-enhanced multi-species Stock Assessment for walleye pollock, Pacific cod, and arrowtooth flounder in the Eastern Bering Sea. Pages 1–43 NPFMC Stock Assessment and Fishery Evaluation Report for the Groundfish Resources of the Bering Sea/Aleutian Islands Regions.

Holsman, K. K., J. Ianelli, K. Aydin, A. E. Punt, and E. A. Moffitt. 2016. A comparison of fisheries biological reference points estimated from temperature-specific multi-species and single-species climate-enhanced stock assessment models. Deep-Sea Research Part II: Topical Studies in Oceanography 134:360–378.

Three harvest simulations are included in the available simulations:
1. Catch = ABC where multispecies assessment simulations (run in ADMB) using climate naive reference points for BO but climate specific B40 and projections (climate effects on growth, M2, and recruitment).
2. Catch = ABC + sloping harvest control rule below B40, and with F = 0 when B < B20.
3. As in 2 but where catch~f(ABC,TAC) via the ATTACH package (below).

4.3. ATTACH model

The catchfunction package (which we refer to as the ABC To TAC and Commercial Harvest, aka ATTACH, model: R package rename forthcoming) was created for the Alaska Climate Integrated Modeling Project (ACLIM) by Amanda Faig (University of Washington; School of Aquatic Fisheries and Sciences) and Alan Haynie (NOAA; NMFS). This function, in a nutshell, takes Bering Sea (BS) acceptable biological catch (ABC) as input and uses a series of regression estimates to predict total allowable catch (TAC) and from that the commercial harvest in the Bering Sea, based on ABC, TAC, and catch data from 1992 to 2017. Version 1.6.0 ATTACH code and documentation used in this study can be found at: http://doi.org/10.5281/zenodo.3966545. Updated documentation and code for ATTACH can be found on the attach github.

Faig, A., and A. Haynie. 2020. The ATTACH model Package Version 1.6.0 http://doi.org/10.5281/zenodo.3966545.
