# Supplementary material for: Ecosystem-based fisheries management forestalls climate-driven collapse
Source: Nat Commun. 2020 Sep 11;11:4579. doi: 10.1038/s41467-020-18300-3 (PMC7486947; doi:10.1038/s41467-020-18300-3)
Supplement: Supplementary file 4 — Supplementary Software [file 41467_2020_18300_MOESM4_ESM.zip › Supplementary_Software/EBSmultispp_2019.pdf]

2019 Climate-enhanced multi-species Stock Assessment for walleye pollock, Pacific cod, and arrowtooth flounder in the Eastern Bering Sea

Kirstin K. Holsman, James N. Ianelli, Kerim Aydin, Ingrid Spies

kirstin.holsman@noaa.gov

November 2019

Alaska Fisheries Science Center, National Marine Fisheries Service, NOAA,
7600 Sand Point Way N.E., Seattle, Washington 98115

Summary of assessment results for 2019:

Biomass

- At 4.9 million tons, the 2019 SEBS pollock spawning biomass is roughly equivalent to the long term (1979-2015) average, however this level represents a 15% decline from 2018 and 23% decline from 2017 spawning biomass levels. Similarly, the downward trend in total biomass observed in the 2018 assessment has continued through 2019, with recent declines placing the total 2019 biomass (14.3 million t) slightly below the 1979-2015 average of 14.9 million tons.
- Similarly,2019 SEBS Pacific cod female spawning biomass has declined 17% since 2018 and 24% since 2017 and 2019 estimates are ~10% below the 1979-2015 average. Total biomass in the SEBS has declined -49% since 2016, and at approximately 635 thousand tons, is 41% below the 1979-2015 average. These patterns are driven in part by continued low survey indices in 2019.
- Arrowtooth total and spawning biomass estimates are 30% and 39% greater than average (respectively), and trends suggest continued stable and increasing biomass from low levels in 2012.
- The multispecies model estimates of a -15% and -17% change in spawning biomass (SSB) between 2018 and 2019 for pollock and Pacific cod (respectively) agree with CEATTLE single species model estimates of decline (-14% and -17%, respectively). Both models predict very little change in spawning biomass for arrowtooth flounder.

Recruitment

- Pollock age 1 recruitment has increased in 2019 for the third year in a row and is 53% above the 1979-2015 average.
- Pacific cod age 1 recruitment remains 32% below average, but has increased in 2019 relative to the record low 2018 recruitment estimate. This trend is consistent with the 2018 assessment climate-enhanced recruitment model projections of increased recruitment in 2019 based on favorable fall foraging conditions preceding record warm 2019 late winter-summer bottom temperatures.
- Estimates of Arrowtooth flounder age 1 recruitment continue to the decline and are 27% below the 1979-2015 average.

Outlook for 2020 and 2021:

Probabilty of Biomass decline or increase:

- Relative to 2019 levels, the model projects SSB of pollock will increase (slightly) in 2020 (projected based on 2019 catch) followed by a decline in SSB in 2021 (projected with F_{ABC}). For Pacific cod the model projects a continued declinein SSB in both 2020 and 2021.
- Ensemble projections using climate-enhanced recruitment models and projected future warming scenarios (including status quo warming, moderate warming, and no warming scenarios) and assuming 2019 catch for 2020 and F_{ABC} for 2021) based on long-term projections estimate a 95% chance that pollock SSB will remain between 96-109% of 2019 SSB in 2020 and will be between 80-87% of 2019 SSB levels in 2021.
- Ensemble projections using climate-enhanced recruitment models based on long-term projections estimate a 95% chance that Pacific cod SSB will continue to decline to between 63-77% of 2019 SSB in 2020 and between 38-43% of 2019 SSB levels in 2021.
- Ensemble projections using climate-enhanced recruitment models based on long-term projections estimate a 95% chance that arrowtooth SSB will be between 94 and 149% of 2019 SSB in 2020 and will be between 88 and 128% of 2019 SSB levels in 2021.

ABC and harvest recommendations

In order to derive ABC estimates the model was projected through the year 2100 to attain relative equilibrium under a climate-naive projection without fishing (B_0, simultaneously for pollock and Pacific cod, then for arrowtooth). Using the approach of Holsman et al. (2016) and Moffitt et al. (2016), the model was then projected under fishing to iteratively solve for the harvest rate (F_{target}) that results in an average of 40% of unfished biomass in the last 5 years of the projection period (2094-2099), with the constraint that spawning biomass under fishing is always greater than 35% unfished biomass during the projection years. F_{target} was then applied to the model to derive ABC and F_{ABC} for 2020 to 2021 projections.

- This method for estimating F_{target} resulted in an proxy ABC harvest rate at equilibrium that corresponds to about 47% SSB_0 for pollock, 50% for Pacific cod, and 40% for arrowtooth flounder for single species models, and about 44%, 50%, and 40% SSB_0 for pollock, Pacific cod, and arrowtooth flounder using the multispecies model.
- Single and multispecies CEATTLE models both project increases in 2020 recommended ABC for pollock over 2019 ABC (from last year's assessment) of 19% and 39%, respectively. 2021 ABC is -3% and 1% of 2019 ABC, respectively.
- Single and multispecies CEATTLE models both project increases in 2020 recommended ABC for Pacific cod over 2019 ABC (from last year's assessment) of 23% and 36%, respectively. While, 2021 ABC is -24% and -25% of 2019 ABC, respectively.
- Single and multispecies CEATTLE models both project a decline and increase (slightly) in 2020 recommended ABC for arrowtooth flounder 2019 ABC (from last year's assessment) of -11% and 1%, respectively. 2021 ABC is -18% and -8% of 2019 ABC, respectively.

Model Summary

This is a three species stock assessment for walleye pollock (*Gadus chalcogrammus*), Pacific cod (*Gadus macrocephalus*) and arrowtooth flounder (*Atheresthes stomias*), from the Eastern Bering Sea (EBS), Alaska updated from Holsman et al.(2016). Results are presented from models estimated and projected without trophic interactions (single-species mode, SSM) and with trophic interactions (multi-species mode, MSM). The main features and settings for this multispecies model include:

- Northern Bering Sea survey data (2010,2017-2019) are not included.
- Predation natural mortality is age specific and annually varying (M2). Residual (non-predation) natural mortality (M1) is age specific but not-annually varying and differs slightly from current assessments for each species (see Table 3 below).
- Predator overlap index is set to 1 for all species (i.e., all prey are available to all predators).
- A moderately "climate-informed" approach is used to derive biological reference points through projecting the model forward with climate effects on weight at age and predation mortality but with a climate-naive Ricker stock recruitment curve (i.e., without environmental covariates).
- Evaluation of F_{ABC} is performed using an ensemble of warming scenarios for 2020 and 2021.
- Weights at age for pollock are based on values from the 2019 SAFE report; for Pacific cod and arrowtooth, they are calculated outside of the model using a temperature-dependent von Bertalanffy to fill in for missing years between 1979-2012 and assume 2012 weight at ages for 2013-2019. For projections, all three species use temperature-specific weights at age using the temperature-dependent von Bertalanffy for 1979-2012.
- Acoustic trawl survey selectivity was set equal to the SAFE report model estimates.
- Fisheries selectivity and survey selectivity are age specific but constant over time.
- Predator-prey suitability is age-specific but constant over time.
- Arrowtooth flounder stock is treated as sexes combined (weight at age and proportion mature is calculated separately for males and females and combined using a mortality-based mean).
- Maturity schedules are based on 2012 assessments and differ slightly from SAFE assessments.
- Projections to derive ABC include a sequential method for determining universal climate-naive B0, and include the constraint that $\text{SSB}_F > 0.35\text{SSB}_0$ for all years in the projection.

Key updates from the 2018 assessment:

- Survey biomass and age composition from the NMFS bottom trawl survey and fishery observer database was updated through 2019. Arrowtooth survey and fishery age composition data were updated with shelf and slope data. Bottom temperature from the survey was updated through 2019.
- Residual natural mortality (M1_{ij} for ages 1-4 of all three species was set to the mean of the total natural mortality from the multispecies assessment. Natural mortality for older Pacific cod was set to 0.38 for to match the Pacific cod stock assessment (Thompson et al. 2018).
- Recruitment forecasts from a climate-enhanced Ricker model were included in the assessment and based on methods in Holsman et al. (in prep).

Key updates from the original Holsman et al. 2016 paper include:

- For single species mode, the residual mortality matches that of current single species assessment models for pollock, Pacific cod, and arrowtooth flounder. The multi-species mode uses the same residual mortality vectors except for the ages 1 and 2 mortality rates for pollock, which were adjusted downward to 0.01 and 0.30, respectively.
- A universal B_0 approach for estimating ABC for single- and multi-species modes.
- Pacific cod fishery composition data is based on lengths rather than model estimates of catch at age.
- Bottom temperature is based on average survey bottom temperatures (observed) for the Bering Sea and is updated through 2019.
- Only one harvest control rule is presented here; find the harvest rate that results in spawning biomass at 40% of unfished biomass (for all three species simultaneously) given that $\text{SSB}_F > 0.35\text{SSB}_0$ for all years in the projection.

As estimated or recommended this year (2019) for:

Quantity	Walleye pollock		Pacific cod		Arrowtooth flounder	
	SSM	MSM	SSM	MSM	SSM	MSM
2019 M (age 1)	1.498	1.492	0.69	0.602	0.65	0.548
2019 Average 3+ M	0.307	0.307	0.38	0.38	0.227	0.227
Projected (age 3+) B_{2020} (t)	10,753,524	10,592,988	430,666	428,104	562,528	569,079
SSB_{2019} (t)	4,812,540	4,907,840	167,214	166,239	378,148	378,954
% change in SSB (t)	-14.2	-15.0	-17.1	-16.9	-1.5	-1.4
Projected SSB_{2020} (t)	5,029,220	5,075,400	118,281	117,726	436,361	437,196
Projected SSB_{2021} (t)	4,463,130	4,059,940	74,146	66,415	425,151	419,454
*Projected $SSB_{0,2100}$ (t)	5,963,739	5,865,572	330,693	343,871	507,282	555,882
*Projected $SSB_{target,2100}$ (t)	2,808,460	2,587,132	165,542	171,322	202,982	222,432
**Target 2100 B/B_0	0.471	0.441	0.501	0.498	0.4	0.4
F_{target}	0.104	0.1	0.334	0.336	0.011	0.011
$F_{ABC,2020}$	0.21	0.305	0.498	0.605	0.047	0.061
ABC_{2020}	2,280,810	3,173,584	143,621	163,493	28,960	37,361
ABC_{2021}	1,860,660	2,317,910	89,016	90,557	26,767	34,105

** $SSB_{0,2100}$ and $SSB_{target,2100}$ are based on the projected SSB at 2100 (equilibrium) given $F = 0$ and $F = F_{target}$, respectively.*
*** Target ratios in 2100 are based on B/B_0=0.4, given that $B/B_0 > 0.35$ for all future yr.*
Projected SSB_{2020} (t) refers to SSB at the start of 2020 and Projected SSB_{2021} (t) refers to SSB at the start of 2021 using $F_{ABC,2020}$ for 2020

As estimated or recommended in 2018 for:

Quantity	Walleye pollock		Pacific cod		Arrowtooth flounder	
	SSM	MSM	SSM	MSM	SSM	MSM
2018 M (age 1)	1.574	1.599	0.675	0.668	0.611	0.512
2018 Average 3+ M	0.308	0.308	0.38	0.38	0.227	0.227
Projected (age 3+) B_{2019} (t)	10,707,945	10,267,813	460,269	450,423	516,849	512,967
SSB_{2018} (t)	5,154,540	5,138,950	157,532	155,408	378,963	376,279
% change in SSB (t)	-11.7	-13.0	-13.5	-13.5	-1.2	-1.1
Projected SSB_{2019} (t)	5,936,460	5,931,980	152,085	150,041	449,872	446,635
Projected SSB_{2020} (t)	4,911,660	4,494,340	138,325	133,529	413,567	405,854
*Projected $SSB_{0,2100}$ (t)	5,890,027	5,454,678	311,210	306,294	488,820	496,647
*Projected $SSB_{target,2100}$ (t)	2,872,060	3,159,189	164,408	168,170	195,537	198,700
**Target 2100 B/B_0	0.488	0.579	0.528	0.549	0.4	0.4
F_{target}	0.112	0.113	0.5	0.508	0.007	0.007
$F_{ABC,2019}$	0.166	0.224	0.309	0.333	0.057	0.066
ABC	2,272,840	2,965,770	132,921	139,485	35,846	41,145
ABC_{2020}	1,914,190	2,283,970	116,827	120,091	32,524	36,956

Figure 1:

Response to SSC and Plan Team comments

Comments specific the Multi-species stock assessment model (CEATTLE)

> *GOA (not EBS):The SSC received a summary of on-going research on the impacts of size selectivity in the winter acoustic trawl survey, an update on research plans for the development of a multispecies model CEATTLE application for the GOA, and the author's plans for introducing alternative models to explore random- walk time-varying values of natural mortality. The SSC agrees with the GOA GPT's concern that the method does not account for size selectivity of the predator. The SSC encourages further exploration of this modeling approach and recommends that results are compared with the CEATTLE model. -SSC Minutes - Oct. 2018 pp. 22*

We are continuing to develop an operational CEATTLE model for the GOA. This work is being led by Grant Adams (UW), Kirstin Holsman, and Martin Dorn and includes multiple enhancements that will likely be applied to the EBS as well in 2020.

Introduction

MSCAA models for evaluating annually varying M

Multi-species statistical catch-at-age models (MSCAA) are an example of a class of multi-species Models with Intermediate Complexity for Ecosystem assessments (i.e., MICE; Plaganyi et al., 2014), which have particular utility in addressing both strategic and tactical EBFM questions (Hollowed et al. 2013; Fogarty 2014; Link and Browman 2014; Plaganyi et al., 2014). MSCAA models may increase forecast accuracy, may be used to evaluate propagating effects of observation and process error on biomass estimates (e.g., Curti 2013; Ianelli et al., 2016), and can quantify climate and trophic interactions on species productivity. As such MSCAA models can address long recognized limitations of prevailing single species management, notably non-stationarity in mortality and biological reference points, and may help reduce risk of over-harvest (Link 2010; Plaganyi et al., 2014; Fogarty 2014). Because multispecies biological reference points (MBRPs) from MSCAA model are conditioned on the abundance of other species in the model (Collie and Gislason 2001; Plaganyi et al., 2014; Fogarty 2014), they may also have utility in setting harvest limits for multi-species fleets, evaluating population dynamics in marine reserves or non-fishing areas, and quantifying trade-offs that emerge among fisheries that impact multiple species in a food web (see reviews in Pikitch et al., 2004; Link 2010; Levin et al., 2013; Link and Browman 2014; Fogarty 2014).

Depending on their structure, MSCAA models can be used to evaluate climate- and fisheries-driven changes to trophodynamic processes, recruitment, and species abundance (Plaganyi et al., 2014). MSCAA models differ somewhat among systems and species, but most use abundance and diet data to estimate fishing mortality, recruitment, stock size, and predation mortality simultaneously for multiple species in a statistical framework. Similar to age structured single species stock assessment models widely used to set harvest limits, MSCAA models are based on a population dynamics model, the parameters of which are estimated using survey and fishery data and maximum likelihood methods (e.g., Jurado-Molina et al., 2005; Kinzey and Punt, 2009; Van Kirk et al., 2010; Kempf 2010; Curti et al., 2013; Tsehaye et al., 2014). Unlike most single-species models (but see Hollowed et al. 2000b; Spencer et al. 2016), MSCAA models additionally separate natural mortality into residual and annually varying predation mortality, and model the latter as a series of predator-prey functional responses. Thus, natural mortality rates for each species in MSCAA models depend on the abundance of predators in a given year and vary annually with changes in recruitment and harvest of each species in the model.

MSCAA models have specific utility in quantifying direct and indirect effects of fisheries harvest on species abundance and size distributions (see reviews in Hollowed et al., 2000a, 2013; Link 2010; Fogarty 2014; Link and Browman 2014; Plaganyi et al., 2014), which is important for EBFM and trade-off analyses of various management strategies. Rapidly shifting climate conditions are also of growing concern in fisheries management as changes in physical processes are known to influence individual growth, survival, and reproductive success of fish and shellfish (Hanson et al., 1997; Kitchell et al., 1977; Morita et al., 2010; Hollowed et al., 2013, Cheung et al., 2015). Climate-driven changes in water temperature can directly impact metabolic costs, prey consumption, and somatic or gonadal tissue growth, with attendant indirect effects on survival, production, and sustainable harvest rates (e.g., Hanson et al., 1997; Morita et al., 2010, Cheung et al., 2015). Temperature-dependent predation, foraging, metabolic, and growth rates are common in more complex spatially-explicit food web or whole of ecosystem models such as GADGET (e.g., Howell and Bogstad 2010; Taylor et al., 2007), Atlantis (e.g., Fulton et al., 2011; Kaplan et al., 2012; 2013), and FEAST (Ortiz et al., 2016). Temperature functions for growth and predation can also be incorporated into MSCAA models, allowing this class of models to be used to evaluate interacting climate, trophodynamic, and fishery influences on recommended fishing mortality rates.

Numerous studies point to the importance of using multi-species models for EBFM (see review in Link 2010). Multi-species production models produced different estimates of abundances and harvest rates than single species models for Northeast US marine ecosystems (Gamble and Link, 2009; Tyrrell et al., 2011), and MSY of commercial groundfish stocks estimated from aggregated production models were different than the sum of MSY estimates from single-species assessments (Mueter and Megrey, 2006; Gaichas et al., 2012; Smith et al., 2015). Multi-species models have been used to demonstrate long-term increases in yield of Icelandic stocks of

Atlantic cod (*Gadus morhua*) and reductions in capelin (*Mallotus villosus*) and Northern shrimp (*Pandalus borealis*) catch associated with short-term decreases in cod harvest (Danielsson et al., 1997). Kaplan et al. (2013) demonstrated the disproportionately large ecosystem impacts of applying the same Fx% (e.g., Fx%, or the harvest rate that reduces spawning stock biomass to x% of unfished spawning stock biomass, SSB0; Caddy and Mahon, 1995; Collie and Gislason, 2001) harvest control rule approach to forage fish as is used for groundfish in the northeast Pacific, and trophodynamics in a southern Benguela ecosystem resulted in higher carrying capacity for small pelagic species under fishing (versus no-fishing) scenarios (Smith et al., 2015).

Since natural mortality and recruitment rates in a MSCAA model are conditioned on harvest rates of predators in the model, an ongoing area of research is evaluating MSCAA model analogs to single-species biological reference points (see Moffitt et al., 2016), such as harvest rates that correspond to maximum yield (FMSY) or proxies thereof (e.g., Fx%). Other multi-species models have been used to derive and evaluate MBRPs, although these have largely focused on MSY (e.g., Kaplan et al., 2013; Smith et al., 2015). A notable exception is Collie and Gislason (2001), who evaluated a variety of MBRPs using a multi-species, virtual population analysis and found MBRPs to be sensitive to variation in natural mortality (much less so to variability in growth), and as such proposed that fishing mortality reference levels for prey species with high mortality be conditioned on the level of predation mortality. Building on this approach, Moffitt et al. (2016) recently demonstrated a projection approach for using multi-species models to derive a variety of MBRPs for EBFM. This provides a basis for the application of MSCAA models for increased use in tactical and strategic EBFM decision-making across a diversity of management frameworks worldwide.

MSCAA for EBM in Alaska

The eastern Bering Sea (Alaska), is defined by large, climate-driven changes to trophodynamics and species productivity that can vary on annual and multi-annual timescales (see reviews in Aydin and Mueter 2007; Hunt et al., 2011; Stabeno et al., 2012; Baker et al., 2014). Accordingly, fisheries management in Alaska has a long history of using ecosystem information and multi-species models for strategic management advice (e.g., multi-species model-based indices, such as mean trophic level, are regularly reported in the annual Ecosystem Considerations chapter of Alaska Stock Assessment and Fishery Evaluation (SAFE) reports; see review in Livingston et al., 2011). Development of multiple MSCAA models in the region (Jurado-Molina et al., 2005; Kinzey and Punt , 2009; Van Kirk, 2010, Holsman et al. 2016) has advanced regional EBFM, facilitating use of estimates from MSCAA models in single-species models used for tactical decisions in the region. For instance,the CEATTLE multispecies model has been included as an appendix to the BSAI pollock assessment (Ianelli et al. 2019) since 2016. Similarly, Dorn et al. (2014) recently evaluated predation mortality estimates from a regional MSCAA model developed by Van Kirk (2010) to inform natural mortality for the Gulf of Alaska walleye pollock (*Gadus chalcogrammus*, hereafter pollock) stock assessment.

The climate-enhanced MSCAA like CEATTLE, have considerable utility in accounting for climate effects on harvest and are useful for species that exhibit strong trophic interactions (predator and prey species) or contrasting management or biological constraints that require simultaneous evaluation (Link 2010). In the eastern Bering Sea, pollock are both predators (adults) and prey (i.e., ages <2; Dunn and Matarese, 1987; Nishiyama et al., 1986) for a variety of species including cannibalistic conspecifics (e.g., Boldt et al., 2012). Variable climate conditions, particularly the spatial extent of winter sea ice, the timing of sea ice spring melt, and subsequent summer bottom temperatures, can differentially promote survival of pollock and their predators and/or modulate predator and prey overlap in the region (e.g., Baily 1989; Zador et al., 2011; Boldt et. al 2012; Hunsicker et al. 2013; Baker and Hollowed 2014). Diet analyses suggest Pacific cod (*Gadus macrocephalus*), cannibalistic conspecifics, and arrowtooth flounder (*Atheresthes stomias*), among others, are important predators of pollock populations in the eastern Bering Sea (Livingston 1993; Aydin and Mueter 2007; Mueter et al., 2007). Climate driven changes to food webs, thermal experience, distribution, and growth collectively impact natural mortality for juveniles, especially juvenile pollock. Accounting for these multispecies interactions is increasingly important under climate-driven change (Holsmane et al. 2019, Karp et al. 2019).

Multispecies model

Here we present a three species climate-enhanced MSCAA model for the Bering Sea (hereafter CEATTLE, for Climate-Enhanced, Age-based model with Temperature-specific Trophic Linkages and Energetics) that includes temperature-dependent von Bertalanffy weight-at-age functions (VBGF; von Bertalanffy, 1938) and temperature-specific, bioenergetics-based predation interactions. CEATTLE, is an example of an environmentally-enhanced stock assessment model (sensu Link 2010), where temperature-specific algorithms predict size-at-age and predation mortality. CEATTLE is programmed in AD model builder (Fournier et al., 2012), and builds on earlier models that combine catch-at-age assessment models with multi-species virtual population analysis (MSVPA) in a statistical framework (i.e., Jurado-Molina et al., 2005). Abundance and biomass of each cohort is modeled using standard population dynamics equations, accounting for a plus age group (Table 1, Eqs. 1, 2). The initial age-structure is assumed to correspond to unfished equilibrium, and the numbers of each species i at age j in year 1 ($N_{0,ij}$) are treated as estimable parameters (Eq.1), such that:

Eq. 1

$$N_{ij,1} = \begin{array}{lll} R_{0,i}e^{\left(-j \text{ M1}_{ij}\right)}N_{0,ij} & y=1 & 1<j<A_i \\ R_{0,i}e^{\left(-j \text{ M1}_{i,A_i}\right)}N_{0,i,A_i}/\left(1-e^{\left(-\text{M1}_{i,A_i}\right)}\right) & y=1 & j \geq A_i \end{array}$$

The number of each species i, age a each year y is then:

Eq. 2

$$\begin{array}{lll} N_{i,j+1,y+1} = N_{i,j,y}e^{-Z_{ij,y}} & 1 \leq y \leq n_y & 1 \leq j < A_i - 1 \\ N_{i,A_i,y+1} = N_{i,A_i-1,y}e^{-Z_{i,A_i-1,y}} + N_{i,A_i,y}e^{-Z_{i,A_i,y}} & 1 \leq y \leq n_y & j \geq A_i \end{array}$$

Total mortality of each prey species i, age j (or predator species p age a) in each year y is the sum of mortality due to predators in the model ($\text{M2}_{ij,y}$), fishing mortality ($\text{F}_{ij,y}$), and residual mortality (M1_{ij}), Eq. T1.6). Predation mortality (Eq. T2.1) is based on the assumption that the annual age-specific ration of a predator is allocated to prey species of a given age according to predator selectivity (Table 2, Eq. T.2.2). Predator selectivity is based on the suitability function derived by Jurado-Molina et al. (2005) and fit to available data from 1981-2015, while annual ration is a function of temperature-specific allometric relationships between ration and fish weight based on bioenergetics models for each species (Eqs. T2.4 and T2.5; see Holsman et al. 2016, and Holsman and Aydin, 2015 for more detail).

The length-to-weight relationships, predator size and species diet preference, bioenergetics-based, temperature-specific predator rations, and maturity were based on previous studies (Tables 1 and 2; Table 5; Holsman et al. Holsman and Aydin, 2015, Holsman et al. 2016). Size-specific diet compositions for each species were assumed known based on diet data collected during the AFSC bottom trawl survey (i.e., diet data were not included in the objective function) and trophic patterns in survey and fishery-based diet data were used to calculate mean (across years and stations) predator-prey suitability (Eq. T2.2).

Temperature specific weight at age

Water temperature is known to directly impact growth through influencing metabolic and digestion rates, which often scale exponentially with body weight and temperature (see Hanson et al., 1997 for an overview). Thus we modified the generalized formulation of the von Bertalanffy growth function (VBGF; von Bertalanffy 1938; Pauly 1981; Temming 1994) to predict temperature-dependent growth by allowing the allometric scaling parameter d to increase with temperature. Essington et al. (2010) and Holsman and Aydin (2015), and Holsman et al. (2016) describe the derivation and application of the VBGF towards bioenergetics modeling in great detail, so we do not repeat it here. Essentially, in this formulation d represents the realized allometric slope of consumption, which integrates both the direct effect of temperature on consumption and indirect ecological interactions that scale with temperature and influence relative foraging rates (see Essington et al., 2010; Holsman and Aydin, 2015). We fit the VBGF to otolith-based length- and weight-at-age data (n

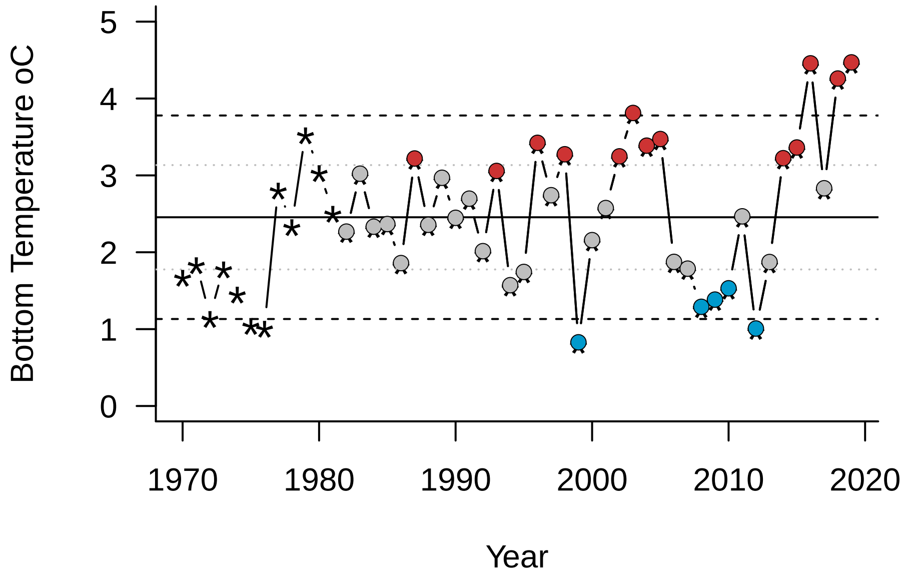

Figure 2: Mean summer bottom temperature for the Eastern Bering Sea (^{o}C); blue and red represent temperatures below or above (respectively) 1 standard deviation of the 1979-2015 mean; dashed lines represent 95% confidence intervals; $*$ represent survey replicated temperature estimates from the Bering 10K regional ocean model.

= 21,388, 14,362, and 772, for pollock, Pacific cod, and arrowtooth flounder, respectively) collected during AFSC Bering Sea surveys and analyzed at the AFSC such that:

Eq. 2 $W_{ij,y} = W_{\infty,iy}(1 - e^{(-K_i(1-d_{i,y})(j-t_{0,i}))})^{1/(1-d_{i,y})}e^{\varepsilon}$, where ε $N(0, \sigma^2_{d,i})$

where $t_{0,i}$ is the age at which $W_{ij,y} = 0$, $W_{\infty,iy}$ is the asymptotic mass which can vary by species i and year y (i.e., $W_{\infty,iy} = (H_i/K_i)^{1(1-d_{i,y})}$), H_i is the assimilation constant K_i is the energy loss constant (Essington et al., 2010), and ε is a normally and independently distributed random variable with mean 0 and variance $\sigma^2_{d,i}$. Essington et al. (2010) and Holsman and Aydin, (2015) statistically estimated the d, K and H parameters for various species to estimate consumption rates. In particular, Holsman and Aydin (2015) found that the d parameter varied between species and regions in Alaska (USA). We further modified this approach to estimate d annually for each year y in the dataset, as a linear function of temperature T_y such that:

Eq. 3 $d_{i,y} = e^{(\alpha_{d,i,y} + \alpha 0_{d,i} + \beta_{d,i} T_y)}$

where $\alpha 0_{d,i}$ and $\alpha_{d,i,y}$ represent the mean d intercept and $\beta_{d,i}$ is the coefficient for the residual effect of temperature on the d consumption parameter. We chose this formulation based on the empirical relationship between temperature and consumption, assuming that d would capture the differential effects of temperature on growth, and that waste rates scale proportionally with weight but do not vary over time with diet or temperature (i.e. K is constant but d can vary with temperature). This formulation allows both the slope and asymptotic limit of growth to vary with temperature. Similar approaches, with slightly different modifications to the VBGF, including temperature and prey specific terms for d and K, respectively, have been used elsewhere to evaluate climate impacts on fish growth (e.g., Cheung et al., 2015; Hamre, 2003).

Table 1. Population dynamics equations for species i and age j in each simulation year y. BT indicates the AFSC bottom trawl survey and EIT represents the echo-integrated acoustic-trawl survey. For all other parameter definitions see Table 3.

Definition	Equation		
Recruitment	$N_{i,1,y} = R_{i,y} = R_{0,i}e^{\tau_{i,y}}$	$\tau_{i,y} \sim N(0,\sigma^2)$	T1.1
Catch (numbers)	$C_{ij,y} = \frac{F_{ij,y}}{Z_{ij,y}}\left(1 - e^{-Z_{ij,y}}\right)N_{ij,y}$		T1.2
Total yield (kg)	$Y_{i,y} = \sum_j^{A_i}\left(\frac{F_{ij,y}}{Z_{ij,y}}\left(1 - e^{-Z_{ij,y}}\right)N_{ij,y}W_{ij,y}\right)$		T1.3
Biomass at age (kg)	$B_{ij,y} = N_{ij,y}W_{ij,y}$		T1.4
Spawning biomass at age (kg)	$SSB_{ij,y} = B_{ij,y}\rho_{ij}$		T1.5
Total mortality at age	$Z_{ij,y} = \text{M1}_{ij} + \text{M2}_{ij} + F_{ij}$		T1.6
Total mortality at age	$F_{ij,y} = F_{0,i}e^{\epsilon_{i,y}S^f_{ij}}$	$\epsilon_{i,y} \sim N\left(0,\sigma^2_{\text{F},i}\right)$	T1.7
Weight at age (kg)	$W_{ij,y} = W_{\infty,iy}\left(1 - e^{(-K_i(1-d_{i,y})(j-t_{0,i}))}\right)^{\frac{1}{1-d_{i,y}}}$		T1.8a
	$d_{i,y} = e^{(\alpha_{d,i,y}+\alpha_{0,d,i}+\beta_{d,i}T_y)}$		T1.8b
	$W_{\infty,iy} = \left(\frac{H_i}{K_i}\right)^{1/(1-d_{i,y})}$		T1.8c
Bottom trawl survey biomass (kg)	$\hat{\beta}^s_{i,y} = \sum_j^{A_i}\left(N_{ij,y}e^{-0.5Z_{ij,y}}W_{ij,y}S^{\text{S}}_{ij}\right)$		T1.9
Acoustic survey biomass (kg)	$\hat{\beta}^{eit}_y = \sum_j^{A_i}\left(N_{1j,y}e^{-0.5Z_{1j,y}}W_{1j,y}S^{eit}_{1j}q^{eit}_{1,j}\right)$	(pollock only)	T1.10
Fishery age composition	$\hat{O}^f_{ij,y} = \frac{C_{ij,y}}{\sum_j C_{ij,y}}$		T1.11
Bottom trawl age composition	$\hat{O}^s_{ij,y} = \frac{N_{ij,y}e^{0.5(-Z_{ij,y})S^{\text{S}}_{ij}}}{\sum_j\left(N_{ij,y}e^{0.5(-Z_{ij,y})S^{\text{S}}_{ij}}\right)}$		T1.12
Acoustic trawl age composition	$\hat{O}^{eit}_{1j,y} = \frac{N_{1j,y}e^{-0.5Z_{1j,y}}S^{eit}_{1j}q^{eit}_{1,j}}{\sum_j\left(N_{1j,y}e^{-0.5Z_{1j,y}}S^{eit}_{1j}q^{eit}_{1,j}\right)}$	(pollock only)	T1.13
Bottom trawl selectivity	$S^{\text{s}}_{ij} = \frac{1}{1+e^{\left(-b^{\text{S}}_i\cdot j-a^{\text{S}}_i\right)}}$		T1.14
Fishery selectivity	$S^f_{ij} = e^{\eta_{ij}} \qquad j \leq A_{\eta,i}$	$\eta_{ij} \sim N\left(0,\sigma^2_{f,i}\right)$	T1.15
	$S^f_{ij} = e^{\eta_{ij}A_{\eta,i}} \qquad j > A_{\eta,i}$		
Proportion female	$\omega_{ij} = \frac{e^{-jM_{\text{fem}}}}{e^{-jM_{\text{fem}}}+e^{-jM_{\text{male}}}}$		T1.16
Proportion of mature females	$\rho_{ij} = \omega_{ij}\phi_{ij}$		T1.17
Adjusted weight at age (kg)	$W_{ij,y} = W^{\text{fem}}_{ij,y}\omega_{ij} + (1-\omega_{ij})W^{\text{male}}_{ij,y}$		T1.18
Adjusted residual natural mortality (kg)	$\text{M1}_{ij} = \text{M1}^{\text{fem}}_{ij}\omega_{ij} + (1-\omega_{ij})\text{M1}^{\text{male}}_{ij,y}$		T1.19

We used this approach to derive annual temperature-specific coefficients of d for pollock and Pacific cod (combined sexes) and separately for male and female arrowtooth flounder (Table 3; Table 5). For arrowtooth flounder, we then used the age-specific proportions of mature females (ρ_{ij}) and males ($1-\rho_{ij}$) to derive the mean weight-at-age for both sexes combined (Eq. T1.18 and Table 5). Lastly, male and female natural mortality rates (M_{male} and M_{fem} , respectively) and age-specific maturity proportions (ϕ_{ij}) from the 2012 stock assessments for eastern Bering Sea pollock (Ianelli et al., 2012), and Bering Sea and Aleutian Islands Pacific cod (Thompson and Lauth, 2012) and arrowtooth flounder (Spies et al., 2012), were used to derive

estimates of the proportion of mature females at age (ρ_{ij}; Eq. T1.17).

Table 2. Predation mortality ($M2$) equations for predators p of age a, and prey i of age j.

Definition	Equation	
Predation mortality	$\text{M2}_{ij,y} = \sum_{pa} \left(\frac{N_{pa,y}\delta_{pa,y}\bar{S}_{paij}}{\left(\sum_{ij} \bar{S}_{paij} B_{ij,y}\right) + B_p^{other}\left(1 - \sum_{ij}\left(\bar{S}_{paij}\right)\right)} \right)$	T2.1
Predator-prey suitability	$\bar{S}_{paij} = \frac{1}{n_y}\sum_y \left(\frac{\frac{\bar{U}_{paij}}{B_{ij,y}}}{\sum_{ij}\left(\frac{\bar{U}_{paij}}{B_{ij,y}}\right) + \frac{1-\sum_{ij}\bar{U}_{paij}}{B_p^{other}}} \right)$	T2.2
Mean gravimetric diet proportion	$\bar{U}_{paij} = \sum_y \frac{U_{paijy}}{n_y}$	T2.3
Individual specific ration (kg yr^{-1})	$\delta_{pa,y} = \hat{\varphi}_{pa,y}\alpha_\delta W_{pa,y}^{(1+\beta_\delta)} f\left(T_y\right)_p$	T2.3
Temperature scaling consumption algorithm	$f\left(T_y\right)_p = V^X e^{(X(1-V))}$	T2.5
	$V = \left(T_p^{cm} - T_y\right) / \left(T_p^{cm} - T_p^{co}\right)$	T2.5a
	$X = \left(Z^2\left(1 + \left(1 + 40/Y\right)^{0.5}\right)^2\right)/400$	T2.5b
	$Z = ln\left(Q_p^c\right)\left(T_p^{cm} - T_p^{co}\right)$	T2.5c
	$Y = ln\left(Q_p^c\right)\left(T_p^{cm} - T_p^{co} + 2\right)$	T2.5d

Parameter estimation & data

The parameters of the model are either pre-specified or estimated by selecting parameters that minimize the log-likelihood function (Table 3) and include fishing mortality rates ($F_{ij,y}$), fishery and survey selectivity (s_{ij}^{f} and s_{ij}^{s} , respectively), initial (pre-harvest) abundance in year 1979 ($N_{0,ij}$), and annual recruitment ($R_{i,y}$), while the estimable parameter of the likelihood function is the catchability coefficient for the acoustic survey (q_1^{eit}; Table 3; Table 4). We fit the model to available survey and fishery data for the eastern Bering Sea including biomass estimates and age-composition data from the annual AFSC summer bottom trawl survey (Eqs. T4.1 and T4.2), biomass and age-composition data from the AFSC Acoustic-trawl (AT) survey (pollock only) (Eqs. T4.3 and T4.4), and the total fishery catch and fishery age-composition data collected by AFSC observers and analyzed at AFSC (Eqs. T4.5 and T4.6; Hilborn and Walters, 1992; Quinn and Deriso, 1999). Penalties were imposed on the changes over age in fishery selectivity (Eq. T4.7). Likelihood priors were applied to normalize the log of annual recruitment and the fisheries mortality deviations, as well as initial abundances (Eqs. T4.8-T4.10). Selectivity for the AT survey was set to previously reported values (Table 3; Honkalehto et al., 2011; Ianelli et al., 2012).

Table 3. Parameter definition (n is the number of parameters for estimated parameters only, value (Plk: Pollock; Cod: Pacific cod; Atf: Arrowtooth flounder both sexes; AtfM: Arrowtooth flounder males; AtfF: Arrowtooth flounder females), and source. I: Input parameter (assigned); M: model index; E: Estimated parameter; F: fixed parameter P: Derived quantity; D: Data.

Parameter	Definition	Type	Value	Source
y	Year	M	$[1, 2, 3 \ldots n_y]$	e
p	Predator	M	$[1, 2, 3 \ldots n_p]$	e
a	Predator age (years)	M	$[1, 2, 3 \ldots A_p]$	e
i	Prey	M	$[1, 2, 3 \ldots n_i]$	e
j	Prey age (years)	M	$[1, 2, 3 \ldots A_i]$	e
n_i	Number of prey species	I	3	e
n_p	Number of predator species	I	3	e
$R_{0,i}$	Mean Recruitment; $n = [1, 1, 1]$	E	≥ 0	e

Parameter	Definition	Type	Value	Source
$\tau_{i,y}$	Annual recruitment deviation; $n = [34, 34, 34]$	E	number	e
$N_{0,ij}$	Initial abundance; $n = [11, 11, 20]$	E	≥ 0	e
$F_{0,i}$	Mean fishing mortality; $n = [1, 1, 1]$	E	≥ 0	e
$\epsilon_{i,y}$	Annual fishing mort. deviation; $n = [34, 11, 20]$	E	number	e
η_{ij}	Fishery age selectivity coef. ; $n = [8, 8, 8]$	E	number	e
b_i^{s}	Survey age selectivity slope; $n = [1, 1, 1]$	E	number	e
a_i^{s}	Survey age selectivity limit ; $n = [1, 1, 1]$	E	number	e
$d_{i,y}$	VBGF allometric slope of consumption	P	≥ 0	e
$W_{\text{inf},iy}$	VBGF max asymptotic weight (kg)	P	> 0	e
ρ_{ij}	Proportion of mature females at age	P	$\in [0, 1]$	e
M1_{ij}	Residual natural mortality	F	≥ 0	e, h
n_y	Number of estimation years	I	41	e
y_0	Start year	I	1979	e
ω_{ij}	Female proportion of population	F	$\in [0, 1]$	c
ϕ_{ij}	Age-specific maturity proportions	F	$\in [0, 1]$	c
$C_{i,y}^{*}$	Observed total yield (kg)	D	≥ 0	f
$O_{ij,y}^{\text{f}}$	Observed fishery age comp.	D	$\in [0, 1]$	f
$O_{ij,y}^{\text{s}}$	Observed BT age comp.	D	$\in [0, 1]$	b
$O_{1j,y}^{\text{eit}}$	Observed AT age comp.	D	$\in [0, 1]$	g
$\beta_{i,y}^{\text{s}}$	Observed BT survey biomass (kg)	D	number	b
β_{y}^{eit}	Observed AT survey biomass (kg)	D	number	g
T_y	Bottom temperature (^oC)	D	number	b
$U_{paij,y}$	Gravimetric proportion of prey in predator stomach	D	$\in [0, 1]$	b
B_p^{other}	Biomass of other prey (kg)	D	$0 \geq$	h
S_{1j}^{eit}	AT survey selectivity	F	$\in [0, 1]$	c

Table 3 (continued). Parameter definition (n is the number of parameters for estimated parameters only, value (Plk: Pollock; Cod: Pacific cod; Atf: Arrowtooth flounder both sexes; AtfM: Arrowtooth flounder males; AtfF: Arrowtooth flounder females), and source. I: Input parameter (assigned); M: model index; E: Estimated parameter; F: fixed parameter P: Derived quantity; D: Data.

Parameter	Definition	Type	Pollock	Cod	ATF	Source
A_i	Number of prey ages	I	12	12	21	e
A_p	Number of predator ages	I	12	12	21	e
$\hat{\varphi}_p$	Annual relative foraging rate (d yr^{-1})	I				d
α_δ	Intercept of the allometric maximum consumption function (g g^{-1} yr^{-1})	I	0.119	0.041	0.125	a
β_δ	Allometric slope of maximum consumption	I	-0.460	-0.122	-0.245	a
T_p^{cm}	Consumption maximum physiological temperature (^{o}C)	I	15.00	21.00	34.13	a
T_p^{co}	Consumption optimum physiological temperature (^{o}C)	I	10.00	13.70	19.60	a
Q_p^c	Max consumption parameter	I	2.60	2.41	2.18	a
$O_{pi,y}$	Spatial predator-prey overlap index	I	1	1	1	e
$\alpha0_{d,i}$	Intercept for VBGF d parameter	F	-0.817	-0.375	M: -0.213<br>F: -0.340	d
$\alpha_{d,i,y}$	Annual intercept for VBGF d	F				
$\beta_{d,i}$	Temperature covariate for VBGF d	F	0.009	0.004	M: -0.0057<br>F: -0.0115	d

Parameter	Definition	Type	Pollock	Cod	ATF	Source
K_i	VBGF energy loss (kg kg^{-1} yr^{-1})	F	0.22	0.45	M: 1.08<br>F: 0.38	d
H_i	VBGF assimilation (kg kg^{-d} yr^{-1})	F	16.34	9.30	M: 5.19<br>F: 5.90	d
$t_{0,i}$	VBGF age when $W_{ij,y}$= 0 (years)	F	0.53	-0.16	M: -1.00<br>F: -0.28	d
M_i^{fem}	Female natural mortality	F	NA*	0.37	0.35	c
M_i^{male}	Male natural mortality	F	NA*	0.37	0.20	c

** pollock age-specific M1 residual mortalities from the assessment were used (same values for male and females).*
a. Holsman and Aydin 2015
b. Alaska Fisheries Science Center eastern Bering Sea bottom trawl survey
c. Stock assessments (Ianelli et al., 2012; Thompson and Lauth, 2012; Spies et al., 2012)
d. Tables 5 & 6
e. This assessment
f. Fishery observer data
g. Alaska Fisheries Science Center echo-integrated acoustic trawl survey
h. Juarado Molina et al., 2005

Harvest scenarios and reference points

For all future scenarios, we set the bottom temperature in the model to the mean of the historical observed temperatures (Fig. 2). We used the approach for deriving biological reference points (BRPs) proposed by Moffitt et al. (2016) and implemented by Holsman et al. (2016). All projections use a Ricker stock recruitment curve without environmental covariates that was fit to model estimates of recruitment and spawning stock biomass. Here we adopted the current over fishing limit (OFL) for Tier 3 acceptable biological catch ABC and MSY proxies for Bering Sea groundfish stocks; 40% of unfished biomass (SSB_0) as the proxy target biomass for the B_{ABC}, and 35% of SSB_0 as the proxy for B_{MSY} (female spawning biomass corresponding to maximum sustainable yield, MSY, i.e., 35% of ; Punt et al., 2014; NPMFC, 2013; Clark et al., 1991; Brooks et al., 2010).

The species-specific, acceptable biological catch ($\text{ABC}_{x,i,y}$) for each harvest scenario (x) was calculated as the fishery yield for each year y of the projection period [1, n_y^{fut}] given a constant fishing mortality rate for the projection period that satisfies each harvest scenario objective ($F^*_{\text{ABC},x,i}$), such that:

Eq. 4 $$\text{ABC}_{x,i,y} = \sum_j^{A_i}((F^*_{\text{ABC},x,i}s^{\text{f}}_{ij}/Z_{x,ij,y})(1 - e^{-Z_{x,ij,y}})N_{x,ij,y}W_{ij,y})$$

where $Z_{x,ij,y}$ is the control-rule specific total annual mortality for species i age j in the set $[1, 2, \dots A_i]$, s^{f}_{ij} is fishery age selectivity, and $N_{x,ij,y}$ and $W_{ij,y}$ are the annual species-specific abundance and weight-at-age for each projection year y. Using this approach, we found the species-specific fishing mortality rate ($F^*_{x,i}$) that results in mean female spawning biomass ($\overline{SSB}_{F,i}$) in the target projection period (i.e., last 5 years; 2046-2050) under fishing that is equal to the target proxy percentage (i.e., 40%) of mean unfished female spawning biomass ($\overline{SSB}_{0,i}$; Table 5). To find $F^*_{\text{ABC},x,i}$, we iteratively project the model to find the $\overline{SSB}_{F,i}$ that corresponds to a given harvest rate $F^*_{x,i}$, adjusting $F^*_{x,i}$ downwards if $\overline{SSB}_{F,i}$ is below the target or upwards if $\overline{SSB}_{F,i}$ is above the target, until we achieve $\overline{SSB}_{F,i}$ near or at the proxy of 40% of $\overline{SSB}_{0,i}$. We ran this harvest scenario with the following variations:

- Project the model through the year 2100 to attain relative equilibrium under a climate-naive projection without fishing (B_0, simultaneously for pollock and Pacific cod, then for arrowtooth).
- Using the approach of Holsman et al. (2016) and Moffitt et al. (2016), the model was then projected under fishing to iteratively solve for the harvest rate (F_{target}) that results in an average of 40% of

unfished biomass in the last 5 years of the projection period (2094-2099), with the constraint that spawning biomass under fishing is always greater than 35% unfished biomass during the projection years.

- $F_{0,target}$ was then applied to the model to derive $\text{ABC}_{target,2020}$ and $F_{ABC,2020}$ for 2020.

We used a combination of observed and modeled 2019 environmental conditions in the Bering Sea and estimates of SSB for each species in 2019 to predict 2020 age 1 recruitment. We modified the approach of Mueter et al. (2011) to estimate recruitment in a given year y as a function of spawning biomass and environmental covariates in the previous year *y-1*. We used Akaike information criterion to evaluate a set of possible candidate models with different covariate combinations, including the null model whereby no covariates were included. We then used each model to project log(recruitment) in 2020 based on conditions in 2019 under 1000 random draws. We present here the mean and 10% and 90% quantile forecasts for age 1 recruitment based on the recruitment model for each species with the highest R^2 value.

Results

Model parameterization

The multi-species mode of the model achieved a slightly higher overall fit to the data (i.e., lower negative log-likelihood with the same number of estimated parameters for both models) for pollock and similar fits to the data for Pacific cod and arrowtooth. Both models fit annual total catch for all three species closely (0.998; Fig. 3). We observed similar fits to survey biomass and age composition data from the single-species (i.e.,$\text{M2}_{ij,y}$ set to 0, hereafter single-species model) and multi-species modes of CEATTLE (Figs. 4,12,13,14,15). Although both models predicted similar historical total and female spawning biomass, inclusion of trophic interactions in the multi-species model resulted in slightly higher estimates of total biomass for pollock (Fig. 4).

Including predation interactions in CEATTLE resulted in similar model fit to observations of survey age composition for pollock, with average annual Pearson correlation coefficient (i.e., R^2) values from CEATTLE model in multi-species mode of 0.87 versus single-species version of CEATTLE model values of 0.87. The single- and multi-species models performed similarly well for the annual Pacific cod and arrowtooth survey age composition data (0.75 for Pacific cod and 0.64 for arrowtooth, respectively). The single- and multi-species models fit the survey estimates of biomass with similar accuracy (single- and multi-species , R^2 respectively, of 0.47 and 0.46 for pollock, 0.78 for both models for Pacific cod, and 0.7 for arrowtooth; negative log-likelihood = 397.72, 667.79, 576.52 and 389.45, 677.18, 575.21 for the single- and multi-species models, respectively). Survey and fishery age selectivity curves were similar for single- and multi-species models for each species (Fig. 5).

Table 4. Correlation coefficients for 1979-2019 survey biomass and age composition data from the model run in single-species mode (SSM) and multi-species mode (MSM).

Table 4.	*SSM*	*MSM*
Total survey biomass		
Pollock	0.47	0.46
Pacific cod	0.78	0.77
Arrowtooth	0.7	0.7
Survey age composition		
Pollock	0.83	0.83
Pacific cod	0.89	0.89
Arrowtooth	0.46	0.47

Predation mortality

Predation mortality varied considerably with changes in predator abundance over time (Figs. 6,7). Total mortality ($M1_{ij}$+$M2_{ij,y}$) for all three species peaked in 2016 (Fig. 6). At 1.49 yr^{-1}, age 1 mortality estimated by the model was greatest for pollock and lower for P. cod and arrowtooth, with total age 1 natural mortality at around 0.69 and 0.65 yr^{-1}. 2019 natural mortality across species is 23% to 33% lower than in 2016 and is no longer above average for pollock (relative to the long-term mean) (Fig. 6), while P. cod and arrowtooth mortality continue to decline and are well below the long-term mean.Total mortality ($M1_{ij}$+$M2_{ij,y}$) for all three species peaked in 2016 (Fig. 6). At 1.49 yr^{-1}, age 1 mortality estimated by the model was greatest for pollock and lower for P. cod and arrowtooth, with total age 1 natural mortality at around 0.69 and 0.65 yr^{-1}. 2019 natural mortality across species is 23% to 33% lower than in 2016 and is no longer above average for pollock (relative to the long-term mean) (Fig. 6), while P. cod and arrowtooth mortality continue to decline and are well below the long-term mean.

Temporal patterns in natural mortality reflect annually varying changes in predation mortality that primarily impact age 1 fish (and to a lesser degree impact ages 2 and 3 fish in the model). Pollock are primarily consumed by older conspecifics, and pollock cannibalism accounts for 87% (on average) of total age 1 pollock consumed annually; Fig. 7), with the exception of the years 2006-2008 when predation by arrowtooth exceeded cannibalism as the largest source of predation mortality of age 1 pollock; Fig. 7). Cannibalistic older conspecifics exhibit a high preference (i.e., total pollock suitability >0.75; Fig. 5) for juvenile pollock (ages 1-3; Fig. 5.g). Larger pollock also appear to target small arrowtooth flounder, as evidenced by a slight increase in total suitability of arrowtooth for pollock ages 6-10 (Fig. 5.g). The relative proportion of pollock consumed by P. cod predators declined in 2019 and is estimated to be roughly equivalent to the proportion consumed by arrowtooth flounder.

Similarly, younger Pacific cod (ages 2-6) also target arrowtooth flounder (Fig. 5.h). Pacific cod increasingly target pollock prey as they age, and larger, older Pacific cod diets are dominated by age 1 pollock prey. Pacific cod also appear to be cannibalistic from ages 4 through 9. In contrast arrowtooth flounder prefer pollock throughout their lives, with total suitability coefficients (for all pollock ages) between 0.5 and 1.0 for arrowtooth flounder ages 1 through 18 (Fig. 5.i). Pollock are both the dominant predator and a primary prey species in the multi-species model, second only to the other prey category (Fig. 5a,b). After other prey and pollock, the next most dominant prey category consumed is Pacific cod, followed by arrowtooth flounder (Fig. 5b).

The total biomass of each species consumed by the predators in the model reflects patterns in age 1 natural mortality. In 2019, the total biomass of pollock consumed by all three predators in the model (typically 1-3 yr old fish) remained slightly above the long-term mean and was similar to 2017 and 2017. Meanwhile, P. cod and arrowtooth biomass consumed was below and near the long-term means, respectively (i.e., within 1 SD of the long-term mean; Fig. 8).

Combined annual predation demand (annual ration) of pollock, P. cod, and arrowtooth flounder in 2019 was 7.3 million tons, down slightly from the 7.86 million t annual average during the warm years of 2014-2016. Walleye pollock represent approximately 79% of the model estimates of combined prey consumed with a long term average of 5.31 million tons of pollock consumed annually by all three predators in the model. Individual annual rations remain significantly above average for all three predator species, driven by annomlously warm water temperautres in the Bering Sea during recent years (Fig. 9).

Implications:

We find evidence of continued decline in predation mortality on age 1 pollock, P. cod and arrowtooth flounder. While warm temperatures continue to lead to high metabolic (and energetic) demand of predators, declines in total predator biomass are contributing to an overall decline in total consumption and therefore reduced predation rates and mortality. This pattern may also explain recent increases in recruitment of EBS pollock over 2017-2019 and P. cod in 2019.

Between 1980 and 1993, relatively high natural mortality rates reflect patterns in combined annual demand

for prey by all three predators that was highest in the mid 1980's (collectively 8.35 million t per year), and in recent years (collectively 7.72 million t per year). The peak in predation mortality of age 1 pollock in 2006 corresponds to the maturation of a large age class of 5-7 year old pollock and 2 year old P. cod that dominated the age composition of the two species in 2006. Similarly, the recent peaks in mortality in 2016 reflect anomalously warm water temperatures combined with the maturation of the large 2010-2012 year class of pollock.

Recruitment

The multi-species version of CEATTLE compensates for elevated predation mortality on younger age classes by increasing estimates of recruitment. Thus, recruitment is higher in the multi-species model than in the single-species model for all three species, especially those with high predation rates (i.e., pollock). The direction of change in annual recruitment estimates from year-to-year was generally the same for both models (i.e., both models increased or decreased recruitment in the same year; Fig. 10a). Pollock recruitment from the single-species version of CEATTLE was slightly positively correlated with Pacific cod recruitment (R^2 = 0.53) and uncorrelated with arrowtooth recruitment (R^2 = 0.01). Correlations between pollock recruitment and Pacific cod or arrowtooth recruitment were similar between the single- and multispecies versions, although correlations were weaker in the multi-species model for Pacific cod (R^2 = 0.38).

Pollock age 1 recruitment has increased in 2019 for the third year in a row and is 53% above the 1979-2015 average.Pacific cod age 1 recruitment remains 32% below average, but has increased in 2019 relative to the record low 2018 recruitment estimate. This trend is consistent with the 2018 assessment climate-enhanced recruitment model projections of increased recruitment in 2019 based on favorable fall foraging conditions preceding record warm 2019 late winter-summer bottom temperatures. Estimates of Arrowtooth flounder age 1 recruitment continue to the decline and are 27% below the 1979-2015 average.

Trends in recruitment may partially reflect climate-driven changes in predation mortality through redistribution of predators (P. cod and pollock) to the Northern Bering sea (not included in this assessment for the SEBS).

Fishing mortality

The single- and multi-species models estimate similar fishing mortality rates for pollock that have remained relatively stable at around 0.13 since the early 1980s (Fig. 11). Both models also estimate low and relatively steady fishing mortality rates for arrowtooth flounder (i.e., ~ 0.03). The adjustment of residual mortality for Pacific cod from 0.37 to 0.38, to match the recent stock assessment (Thompson et al. 2018), results in slightly higher estimates of fishing mortality over time (0.22-0.45) as compared to the 2016 assessment (0.24-0.45), with indications of slight increases in fishing mortality in recent years (Fig. 11).

Harvest scenarios and reference points

Projecting CEATTLE forward under mean recruitment produces trajectories of female spawning stock biomass that can be used to derive multi-species biological reference points and attendant fishing mortality rates (Holsman et al. 2016). Projections under the Ricker spawner-recruit model lead to some overcompensation recruitment dynamics for pollock in the first years of the projection (especially for single-species models; Fig. 16; sensu Botsford, 1986). However, a >70 year projection period was sufficient to allow such dynamics to reach a relative equilibrium (Figs. 16,17).

In general, unfished and harvested female spawning stock biomass ($SSB_{0,iy}$ and $SSB_{target,iy}$, respectively) were similar for projections of the multi- than the single-species model (Figs. 16,20) due to adjusted age 1 M_1 values for the single species model that match the mean (across years) age 1 M_2 from the multispecies model.

Inclusion of trophic interactions (predation) in the model resulted in slightly different stock-recruitment curves (Figs. 18, 19), with stronger density dependence for pollock in the single-species model than in the multispecies model (where density dependence would be from non-predation interactions), and peak recruitment occurring at much higher spawning biomass levels (i.e., ~3.5 million tons versus ~2 million tons for the single species model).

Relative to 2019 levels, the multispecies model projects SSB of pollock will increase (slightly) in 2020 (projected based on 2019 catch) followed by a decline in SSB in 2021 (projected with F_{ABC}). For Pacific cod the model projects a continued declinein SSB in both 2020 and 2021.

Ensemble projections using climate-enhanced recruitment models and projected future warming scenarios (including status quo warming, moderate warming, and no warming scenarios) and assuming 2019 catch for 2020 and F_{ABC} for 2021) based on long-term projections estimate a 95% chance that pollock SSB will remain between 96-109% of 2019 SSB in 2020 and will be between 80-87% of 2019 SSB levels in 2021.Ensemble projections using climate-enhanced recruitment models based on long-term projections estimate a 95% chance that Pacific cod SSB will continue to decline to between 63-77% of 2019 SSB in 2020 and between 38-43% of 2019 SSB levels in 2021. Ensemble projections using climate-enhanced recruitment models based on long-term projections estimate a 95% chance that arrowtooth SSB will be between 93 and 148% of 2019 SSB in 2020 and will be between 88 and 128% of 2019 SSB levels in 2021.

In order to derive ABC estimates the model was projected through the year 2100 to attain relative equilibrium under a climate-naive projection without fishing (B_0, simultaneously for pollock and Pacific cod, then for arrowtooth). Using the approach of Holsman et al. (2016) and Moffitt et al. (2016), the model was then projected under fishing to iteratively solve for the harvest rate (F_{target}) that results in an average of 40% of unfished biomass in the last 5 years of the projection period (2094-2099), with the constraint that spawning biomass under fishing is always greater than 35% unfished biomass during the projection years. F_{target} was then applied to the model to derive ABC and F_{ABC} for 2020 to 2021 projections.

This method for estimating F_{target} resulted in an proxy ABC harvest rate at equilibrium that corresponds to about 47% SSB_0 for pollock, 50% for Pacific cod, and 40% for arrowtooth flounder for single species models, and about 44%, 50%, and 40% SSB_0 for pollock, Pacific cod, and arrowtooth flounder using the multispecies model.

Single and multispecies CEATTLE models both project increases in 2020 recommended ABC for pollock over 2019 ABC (from last year's assessment) of 19% and 39%, respectively. 2021 ABC is -3% and 1% of 2019 ABC, respectively.Single and multispecies CEATTLE models both project increases in 2020 recommended ABC for Pacific cod over 2019 ABC (from last year's assessment) of 23% and 36%, respectively. While, 2021 ABC is -24% and -25% of 2019 ABC, respectively. Single and multispecies CEATTLE models both project a decline and increase (slightly) in 2020 recommended ABC for arrowtooth flounder 2019 ABC (from last year's assessment) of -11% and 1%, respectively. 2021 ABC is -18% and -8% of 2019 ABC, respectively.

Application of MBRPs toward climate-resilient EBFM

Development of climate-informed multi-species biological reference points (MBRPs) from climate-enhanced models like CEATTLE is a necessary step in managing for cliamte impacts on fisheries resources (Holsman et al. 2019; Karp et al. 2019; Link, 2010; Link and Browman, 2014). Projecting CEATTLE provides proxies for MBRPs that can readily be implemented in current OFL control rules for Alaska fisheries management and demonstrates the range of possible considerations as well as individual strengths and weaknesses of each control rule approach. Like previous authors, we found that ABC proxies were lower than the single-species CEATTLE model estimates (e.g., Gaichas et al., 2012). That said, Holsman et al. (2016) found that MBRPs do not inherently result in lower harvest recommendations than single-species corollaries (i.e., BRPs); comparative risk of over- or under-harvest depends on the degree of inter-specific predation and cannibalism. They also found that recommended harvest rates were relatively consistent between harvest scenarios, especially if target minimum biomass is included for individual species. They also found that climate and trophic drivers can interact to affect MBRPs, but for prey species with high predation rates, trophic and management-driven changes may exceed direct effects of temperature on growth and predation.

Given this, MSCAA models can readily be used for tactical EBFM decisions under changing climate conditions, if, as suggested by Holsman et al. (2016) and by various authors previously, harvest scenarios used for deriving MBRPs combined a minimum biomass threshold with yield targets to meet biodiversity and yield objectives (Worm et al., 2009; Gaichas et al., 2012). Biomass thresholds will require development of criteria for minimum limits in order represents a necessary advancement of the current approach.

Short-term utility: potential application within current single species assessments

This work demonstrates some alternative applications of multispecies trophic models within a management setting and there may be immediate relevance for current stock assessment models. For example, the estimated historical time series of natural mortality at age over time (M1 + M2) could be used directly within the assessment or used as priors in alternative assessment models with estimated annually varying natural mortality. Similarly, for the case of EBS pollock, the stock recruitment relationship may provide a basis for better estimates or prior distribution specification. It may be that by adding the time series of estimated total natural mortality at age that the estimated stock recruitment relationship may differ substantially given the relative differences in age 1 abundances. Further research on applying alternative stock recruitment relationships is needed as well, especially since the application of the Ricker curve has traditionally been justified due to cannibalistic nature of pollocka situation that is partially accounted for in this application.

Long-term utility: climate- and trophic-specific biological reference points

Long-term projections of climate conditions (ideally ensembles to capture future uncertainty) are needed to inform long-term climate-specific reference points (Holsman et al. 2019), while short-term forecasts (e.g., <2 years) would also advance near-term understanding of harvest and productivity reference points, especially change in weight at age and survival within the 2 year harvest specification period (Karp et al. 2019). In this, the model could be combined with short-term forecasts of physical and lower trophic conditions in the Bering Sea, and used to refine estimates of recruitment and spawning stock biomass under changing conditions. Extensive ROMSNPZ model validation is currently underway will be useful for evaluating predictive performance and potential utility going forward. Incorporating additional species into the model, such as northern fur seals and Pacific halibut could help provide quantitative estimates of changes in juvenile pollock forage resources associated with different harvest rates of groundfish species in the EBS, as well as refine estimates of predation mortality for prey species in the model under changing conditions. Finally, ongoing incorporation of harvest scenarios into the model will add realism to projections both for assessment purposes and for research applications.

Acknowledgments

Our work is the result of numerous collaborations with researchers at the University of Washington (UW), University of Alaska Fairbanks (UAF), and the NOAA Alaska Fisheries Science Center (AFSC) and Northwest Fisheries Science Center (NWFSC). In particular, Ron Heintz (AFSC), Franz Mueter (UAF), and Elizabeth Siddon (UAF) supported an excellent discussion of the bioenergetics model sub-component of CEATTLE. We thank Grant Adams (UW), Grant Thompson (AFSC), I. Kaplan (NWFSC), and P. Sean McDonald (UW) for providing feedback on previous drafts. Drs. E. Moffitt and A. Punt contributed significantly to the development of multispecies harvest control rules used in this assessment. Support for the CEATTLE model came from the Alaska Integrated Ecosystem Assessment program (noaa.gov/iea), the NMFS Fisheries And The Environment (FATE) program, the Stock Assessment Analytical Methods program under award number 0002, and the North Pacific Research Board (publication number 547). This effort would not be possible without the help of numerous researchers and volunteers who contribute annually to the collection of biomass, demography, and diet information through Alaska Fishery Science Center

surveys and the NOAA observer program, and the help of those who provide access to fishery-dependent and independent data through the Alaska Fisheries Science Center.

References

Botsford, L. W., 1986. Effects of environmental forcing on age-structured populations: Northern California Dungeness crab (Cancer magister) as an example. Can. J. Fish. Aquat. Sci. 43, 2345-2352.

Brooks, E. N., Powers, J. E., and Cort??s, E., 2010. Analytical reference points for age-structured models: application to data-poor fisheries. ICES J. Mar. Sci., 67, 165175.

Caddy, J. F., Mahon, R., 1995. Reference points for fishery management. FAO Fisheries Technical Paper 347.

Cheung, W.W.L., Brodeur, R. D., Okey, T. A., Pauly, D., 2015. Projecting future changes in distributions of pelagic fish species of Northeast Pacific shelf seas. Prog. Oceanogr. 130, 1931.

Clark, W. G., 1991. Groundfish exploitation rates based on life history parameters. Can. J. Fish. Aquat. Sci. 48, 734750.

Collie, J. S., Gislason, H., 2001. Biological reference points for fish stocks in a multispecies context. Can. J. Fish. Aquat. Sci. 58, 2167-2176.

Coyle, K. O., Eisner L. B., Mueter F. J., Pinchuk A. I., Janout M. A.,Cieciel, K. D., Farley, E.V., Andrew, A. G., 2011. Climate change in the southeastern Bering Sea: impacts on pollock stocks and implications for the Oscillating Control Hypothesis. Fish. Ocean. 20(2), 139156.

Curti, K. I., Collie, J. S., Legault, C. M., and Link, J. S., 2013. Evaluating the performance of a multispecies statistical catch-at-age model. . Can. J. Fish. Aquat. Sci. 70, 470-484.

Danielsson, A., Stefansson, G., Baldursson, F. M., Thorarinsson K., 1997. Utilization of the Icelandic cod stock in a multispecies context. Mar. Res. Econ. 12(4), 329-344.

Dorn, M., Aydin, K., Jones, D., Palsson, W., Spalinger, K., 2014. Chapter 1: Assessment of the Walleye Pollock Stock in the Gulf of Alaska. In Stock Assessment and Fishery Evaluation Report for the Groundfish Resources of the Gulf of Alaska Region, Alaska Fisheries Science Center, National Marine Fisheries Service, Anchorage, AK, p 53170.

Dunn, J. R. Matarese, A. C., 1987. A review of the early life history of Northeast Pacific gadoid fishes. Fish. Res. 5, 165-184.

Gaichas, S., Gamble, R., Fogarty, M., Beno??t, H., Essington, T., Fu, C., Koen-Alonso, M., Link, J., 2012. Assembly rules for aggregate-species production models: simulations in support of management strategy evaluation. Mar. Eco. Prog. Ser. 459, 275292.

Gamble R. J. and Link, J. S., 2009. Analyzing the tradeoffs among ecological and fishing effects on an example fish community: a multispecies (fisheries) production model. Ecol. Model. 220, 2570-2582.

Gamble, R. J. and Link, J., 2012. Using an aggregate production simulation model with ecological interactions to explore effects of fishing and climate on a fish community. Mar. Eco. Prog. Ser. 459, 259274, 2012 doi: 10.3354/meps09745

Gislason, H. 1999. Single and multispecies reference points for Baltic fish stocks. ICES J. Mar. Sci. 56, 571-583.

Essington, T., Kitchell J., Walters, C., 2001. The von Bertalanffy growth function, bioenergetics, and the consumption rates of fish. Can. J. Fish. Aquat. Sci. 58, 21292138.

Fogarty, M. J. 2014. The art of ecosystem-based fishery management. Can. J. Fish. Aquat. Sci. 71, 479-490.

Fogarty, M.J., Overholtz, W. J., Link, J. S., 2012. Aggregate surplus production models for demersal fishery resources of the Gulf of Main. Mar. Ecol. Prog. Ser. 459, 247-258.

Fournier, D. A., Skaug, H. J., Ancheta, J., Ianelli, J., Magnusson, A., Maunder, M. N., Nielsen, A., Sibert, J., 2012. AD Model Builder: using automatic differentiation for statistical inference of highly parameterized complex nonlinear models. Optimization Methods and Software, 27, 233-249.

Fulton, E. A., Link, J. S., Kaplan, I. C., Savina-Rolland, M., Johnson, P., Ainsworth, C., Horne, P., Gorton, R., Gamble, R.J., Smith, A.D.M., Smith, D.C., 2011. Lessons in modelling and management of marine ecosystems: the Atlantis experience. Fish Fish. 12, 171188.

Hanson, P., Johnson, T. Schindler, D., Kitchell, J., 1997. Fish Bioenergetics 3.0. Madison, WI: University of Wisconsin Sea Grant Institute.

Hamre, J. 2003. Capelin and herring as key species for the yield of north-east Arctic cod. Results from multispecies model runs. Sci. Mar. 67 (Suppl 1), 315-323.

Hilborn, R. and Walters, C. J., 1992. Quantitative Fisheries Stock Assessment: Choice, Dynamics and Uncertainty. Chapman and Hall, New York. 570 p.

Hollowed, A. B., Bax, N., Beamish, R., Collie, J., Fogarty, M., Livingston, P., Pope, J., Rice, J. C., 2000a. Are multispecies models an improvement on single-species models for measuring fishing impacts on marine ecosystems? ICES J. Mar. Sci., 57, 707719. doi:10.1006/jmsc.2000.0734.

Hollowed, A. B., Ianelli, J. N., and Livingston, P. A., 2000b. Including predation mortality in stock assessments: a case study for Gulf of Alaska walleye Pollock. ICES J. Mar. Sci., 57, 279293.

Hollowed, A. B., Curchitser, E. N., Stock, C. A., Zhang, C. 2013. Trade-offs associated with different modeling approaches for assessment of fish and shellfish responses to climate change. Climatic Change 119, 111129 DOI 10.1007/Table 6584-012-0641-z

Holsman, K. K., Ianelli, J., Aydin, K., Punt, A. E., Moffitt , E. A. (2016). Comparative biological reference points estimated from temperature-specific multispecies and single species stock assessment models. Deep Sea Res. II. doi:10.1016/j.dsr2.2015.08.001.

Holsman, K. K. and Aydin, K. 2015. Comparative methods for evaluating climate change impacts on the foraging ecology of Alaskan groundfish. Mar. Ecol. Prog. Ser. DOI 10.3354/mep102

Holsman et al. in prep. Differential impacts of climate change on Bering Sea groundfish recruitment. Mar Ecol Prog Ser.

Holsman, KK, EL Hazen, A Haynie, S Gourguet, A Hollowed, S Bograd, JF Samhouri, K Aydin. 2019. Toward climate-resiliency in fisheries management. ICES Journal of Marine Science. 10.1093/icesjms/fsz031

Honkalehto, T., Ressler, P.H., Towler, R.H., Wilson, C.D., 2011.Using acoustic data from fishing vessels to estimate walleye pollock (Theragra chalcogramma) abundance in the eastern Bering Sea. 2011. Can. J. Fish. Aquat. Sci. 68, 12311242

Howell, D., Bogstad, B. 2010. A combined Gadget/FLR model for management strategy evaluations of the Barents Sea fisheries. ICES J. Mar. Sci, 67, 000000.

Hunsicker, M. E., Ciannelli, L., Bailey, K. M., Zador, S., Stige, L. C., 2013. Climate and demography dictate the strength of predator-prey overlap in a subarctic marine ecosystem. PloS one 8(6), e66025. doi: 10.1371/journal.pone.0066025.

Hunt G. L. Jr, Coyle K. O., Eisner L., Farley E. V., Heintz R., Mueter, F., Napp, J. M., Overland, J. E., Ressler, P. H., Salo, S., Stabeno, P. J., 2011. Climate impacts on eastern Bering Sea foodwebs: A synthesis of new data and an assessment of the Oscillating Control Hypothesis. ICES J. Mar. Sci. 68(6), 12301243.

Ianelli, J. N., Honkalehto T., Barbeaux S., Kotwicki S., 2014. Chapter 1: Assessment of the walleye pollock stock in the Eastern Bering Sea. In Stock Assessment and Fishery Evaluation Report for the Groundfish Resources of the Bering Sea/Aleutian Islands Regions, Alaska Fisheries Science Center, National Marine Fisheries Service, Anchorage, AK, p 55156.

Ianelli, J. N., Barbeaux, S., Honkalehto, T., Kotwicki, S., Aydin, K., and Williamson, N. 2012. Assessment of Alaska Pollock Stock in the eastern Bering Sea. In Stock Assessment and Fishery Evaluation Report for the Groundfish Resources of the Bering Sea/Aleutian Islands Regions, pp. 31124.

Ianelli, J. N., et al. 2019. Assessment of Alaska Pollock Stock in the eastern Bering Sea. In Stock Assessment and Fishery Evaluation Report for the Groundfish Resources of the Bering Sea/Aleutian Islands Regions.

Ianelli, J. N., Holsman, K. K. Punt, A. E., Aydin, K. 2016. Multi-model inference for incorporating trophic and climate uncertainty into stock assessments. Deep Sea Res. II

Jurado-Molina, J., Livingston, P. A., Ianelli, J. N., 2005. Incorporating predation interactions in a statistical catch-at-age model for a predator-prey system in the eastern Bering Sea. Can. J. Fish. Aquat. Sci. 62, 1865-1873.

Kaplan, I. C. , P. J. Horne, P. S. Levin., 2012. Screening California Current fishery manamgent scenarios using the Atlantis end-to-end ecosystem model. Prog. Oceanogr. 102, 5-18.

Kaplan, I.C., Brown, C.J., Fulton, E.A., Gray, I.A., Field, J.C., Smith, A.D.M., 2013. Impacts of depleting forage species in the California Current. Environ. Cons. 40, 380393.

Karp, M ,JO Peterson, PD Lynch, RB Griffis, C Adams, B Arnold, L Barnett, Y deReynier, J DiCosimo, K Fenske, S Gaichas, A Hollowed, K Holsman, + 13. 2019. Accounting for Shifting Distributions and Changing Productivity in the Development of Scientific Advice for Fishery Management. ICES Journal of Marine Science fsz048, https://doi.org/10.1093/icesjms/fsz048

Kinzey, D. Punt, A. E., 2009. Multispecies and single-species age-structured models of fish population dynamics: Comparing parameter estimates. Nat. Res. Mod. 22, 67-104.

Kitchell, J. F., Stewart, D. J. and Weininger, D., 1977. Applications of a bioenergetics model to yellow perch (Perca flavenscens) and walleye (Stizostedion vitreum vitreum). J. Fish. Res. Board Can. 34, 1922-1935.

Levin, P. S., Kelble, C. R., Shuford, R., Ainsworth, C., deReynier, Y., Dunsmore, R., Fogarty, M. J., Holsman, K., Howell, E., Monaco, M., Oakes, S., Werner, F., 2013. Guidance for implementation of integrated ecosystem assessments: a US perspective. ICES J. Mar. Sci, doi:10.1093/icesjms/fst112.

Link J. S., 2010. Ecosystem-based fisheries management: confronting tradeoffs, Cambridge University Press, Cambridge.

Link, J. S., Browman, H. I., 2014. Integrating what? Levels of marine ecosystem-based assessment and management. ICES J. Mar. Sci, 71, 11701173

Livingston, P. A., Aydin, K., Bolt, J. L., Hollowed , A. B., Napp, J. M., 2011. Alaskan marine fisheries management: advances and linkages to ecosystem research. In A Belgrano and W Fowler (eds.), Ecosystem-Based Management for Marine Fisheries: An Evolving Perspective. Cambridge University Press, pp 113-152.

Livingston, P., 1993. Importance of predation by groundfish, marine mammals and birds on walleye pollock Theragra chalcogramma and Pacific herring Clupea pallasi in the eastern Bering Sea. Mar. Ecol. Prog. Ser. 102(3), 205215.

Moffitt, E., Punt, A. E., Holsman, K. K., Aydin, K. Y., Ianelli, J. N., Ortiz, I., 2016. Moving towards Ecosystem Based Fisheries Management: options for parameterizing multi-species harvest control rules. Deep Sea Res. II.

Morita, K., Fukuwaka, M. A., Tanimata, N. and Yamamura, O., 2010. Size-dependent thermal preferences in a pelagic fish. Oikos 119, 1265-1272.

Murawski, S., Matlock G., 2006. Ecosystem science capabilities required to support NOAAs mission in the year 2020. NOAA Technical Memorandum, NMFS-F/SPO-74, Silver Spring, MD.

Mueter, F. J. Megrey, B. A., 2006. Using multispecies surplus production models to estimate ecosystemlevel maximum sustainable yields. Fish. Res. 81, 189201.

Mueter, F. J., Boldt, J. L., Megrey, B. A., Peterman, R. M., 2007. Recruitment and survival of Northeast Pacific Ocean fish stocks: temporal trends, covariation, and regime shifts. Can. J. Fish. Aquat. Sci. 64(6), 911-927.

Mueter, F. J., Bond, N. A., Ianelli, J. N., & Hollowed, A. B. (2011). Expected declines in recruitment of walleye pollock (Theragra chalcogramma) in the eastern Bering Sea under future climate change.

Nishiyama, T., Hirano, K., and Haryu, T., 1986. The early life history and feeding habits of larval walleye pollock Theragra chalcogramma (Pallas) in the southeast Bering Sea. Int. North Pac. Fish. Comm. Bull. 45, 177227.

North Pacific Fishery Management Council (NPFMC). 2013. Fishery Management plan for groundfish of the Bering Sea and Aleutian Islands management area. North Pacific Fishery Management Council, Anchorage, AK.

Ortiz, I, K. Aydin, A. J. Hermann, G. Gibson. 2016. Climate to fisheries: Exploring processes in the eastern Bering Sea based on a 40 year hindcast. Deep Sea Res. II.

Pauly, D., 1981. The relationship between gill surface area and growth performance in fish: a generalization of von Bertalanffys theory of growth. Meeresforschung 28, 251-282.

Plaganyi, ??. E., Punt, A.E., Hillary, R., Morello, E.B., Th??baud, O., Hutton, T., Pillans, R.D., Thorson, J.T., Fulton, E. A., Smith, A. D. M., Smith, F., Bayliss, P., Haywood, M., Lyne, V., Rothlisberg, P.C., 2014. Multispecies fisheries management and conservation: tactical applications using models of intermediate complexity. Fish Fish. 15, 1-22.

Pikitch E. K., Santora C., Babcock E. A., Bakun A., Bonfi, R., Conover, D. O., Dayton, P., Doukakis, P., Fluharty, D., Heneman, B., Houde, E. D., Link, J., Livingston, P. A., Mangel, M., McAllister, M. K., Pope, J., Sainsbury, K. J., 2004. Ecosystem-based fishery management. Science 305, 346347

Punt, A.E., Smith, A.D.M., Smith, D.C., Tuck, G., Klaer, N., 2014. Selecting relative abundance proxies for BMSY and BMEY. ICES J. Mar. Sci. 71, 469-483.

Quinn, T. J., II, Deriso, R. B., 1999. Quantitative Fish Dynamics. Oxford University Press, New York.

Ricker, W. E. (1954) Stock and Recruitment Journal of the Fisheries Research Board of Canada, 11(5): 559623. doi:10.1139/f54-039

Siddon E. C., Kristiansen T., Mueter F. J., Holsman K. K., Heintz R. A., Farley, E. V., 2013. Spatial Match-Mismatch between Juvenile Fish and Prey Provides a Mechanism for Recruitment Variability across Contrasting Climate Conditions in the Eastern Bering Sea. PLoS ONE 8(12), e84526. doi:10.1371/journal.pone.0084526

Smith, M. D., Fulton, E. A., and Day, R.W. 2015. An investigation into fisheries interaction effects using Atlantis. ICES J. Mar. Sci, 72(1), 275283. doi:10.1093/icesjms/fsu114

Spencer, PD, KK Holsman, S Zador, NA Bond, FJ Mueter, AB Hollowed1, and JN Ianelli. (2016). Modelling spatially dependent predation mortality of eastern Bering Sea walleye pollock, and its implications for stock dynamics under future climate scenarios. ICES Journal of Marine Science; doi:10.1093/icesjms/fsw040

Spies, I. Wilderbuer, T. K., Nichol, D. G. and Aydin, K., 2012. Chapter 6. Arrowtooth Flounder. In Stock Assessment and Fishery Evaluation Report for the Groundfish Resources of the Bering Sea/Aleutian Islands Regions, pp. 31124.

Stabeno, P.J., Farley, E.V., Jr., Kachel, N.B., Moor, S., Mordy, C.W., Napp, J.M., Overland, J.E., Pinchuk, A.I., Sigler, M.F., 2012. A comparison of the physics of the northern and southern shelves of the eastern Bering Sea and some implications for the ecosystem. Deep Sea Res. II 65-70, 14-30.

Gouhier, T. C., Guichard, F., Gonzalez, A., 2010. Synchrony and Stability of Food Webs in Metacommunities. Am. Nat., 175 (2), E16-E34

Taylor, L. , Begley, J., Kupca1, V. Stefansson, G., 2007. A simple implementation of the statistical modelling framework Gadget for cod in Icelandic waters. Afr. J. Mar. Sci., 29(2), 223245

Temming, A., 1994. Food conversion efficiency and the von Bertalanffy growth function. Part II and conclusion: extension of the new model to the generalized von Bertalanffy growth function. NAGA The ICLARM Quarterly, 17(4), 41-45.

Tsehaye, I., Jones, M. I., Bence, J. R., Brenden, T. O., Madenjian, C. P., Warner, D. M., 2014. A multispecies statistical age-structured model to assess predator-prey balance: application to an intensively managed Lake Michigan pelagic fish community. Can. J. Fish. Aquat. Sci. 71, 627-644.

Thompson, G. G., Lauth, R. R., 2012. Chapter 2: Assessment of the Pacific Cod Stock in the Eastern Bering Sea and Aleutian Islands Area. In Stock Assessment and Fishery Evaluation Report for the Groundfish Resources of the Bering Sea/Aleutian Islands Regions, pp. 31124.

Tyrrell M. C., Link J. S., Moustahfid H., 2011. The importance of including predation in some fish population models: implications for biological reference points. Fish. Res. 108, 1-8.

Van Kirk, K. F., Quinn II, T. J., Collie, J. S., 2010. A multispecies age-structured assessment model for the Gulf of Alaska. Can. J. Fish. Aquat. Sci. 67, 1135-1148.

von Bertalanffy, L., 1938. A quantitative theory of organic growth. Hum. Biol. 10: 181-213.

Worm B., Hilborn R., Baum J. K., Branch T. A. Collie, J. S., Costello, C., Fogarty, M. J., Fulton, E. A., Hutchings, J. A., Jennings, S., Jensen, O. P., Lotze, H. K., Mace, P. M., McClanahan, T. R., Minto, C., Palumbi, S. R., Parma, A. M., Ricard, D. , Rosenberg, A. A., Watson, R., Zeller, D., 2009. Rebuilding global fisheries. Science 325, 578585

Zador S., Aydin K., Cope J., 2011. Fine-scale analysis of arrowtooth flounder Atherestes stomias catch rates reveals spatial trends in abundance. Mar. Ecol. Prog. Ser. 438, 229-239

Figures & Tables

Table 5. Proportion mature (ρ_{ij}) and residual natural mortality ($\mathrm{M1}_{ij}$) for each species i and age j in the single-species (ssm) or multi-species model (msm) for wallleye pollock (plk), Pacific cod (pcod), and Arrowtooth flounder (atf).

Age	1	2	3	4	5	6	7	8	9	10	11	12	13	14	15	16	17	18	19	20	21
ρ_{ij}																					
plk	0.00	0.01	0.29	0.64	0.84	0.90	0.95	0.96	0.97	1.00	1.00	1.00									
pcod	0.00	0.02	0.06	0.14	0.30	0.53	0.75	0.89	0.95	0.98	0.99	1.00									
atf	0.00	0.00	0.01	0.02	0.06	0.16	0.34	0.59	0.80	0.92	0.97	0.99	1.00	1.00	1.00	1.00	1.00	1.00	1.00	1.00	1.00
SSM																					
$\mathrm{M1}_{ij}$																					
plk	0.90	0.45	0.30	0.30	0.30	0.30	0.30	0.30	0.30	0.30	0.30	0.30									
pcod	0.34	0.34	0.34	0.34	0.34	0.34	0.34	0.34	0.34	0.34	0.34	0.34									
atf	0.27	0.26	0.26	0.25	0.25	0.24	0.24	0.23	0.23	0.23	0.22	0.22	0.22	0.22	0.21	0.21	0.21	0.21	0.21	0.21	0.21
MSM																					
$\mathrm{M1}_{ij}$																					
plk	0.01	0.30	0.30	0.30	0.30	0.30	0.30	0.30	0.30	0.30	0.30	0.30									
pcod	0.34	0.34	0.34	0.34	0.34	0.34	0.34	0.34	0.34	0.34	0.34	0.34									
atf	0.27	0.26	0.26	0.25	0.25	0.24	0.24	0.23	0.23	0.23	0.22	0.22	0.22	0.22	0.21	0.21	0.21	0.21	0.21	0.21	0.21

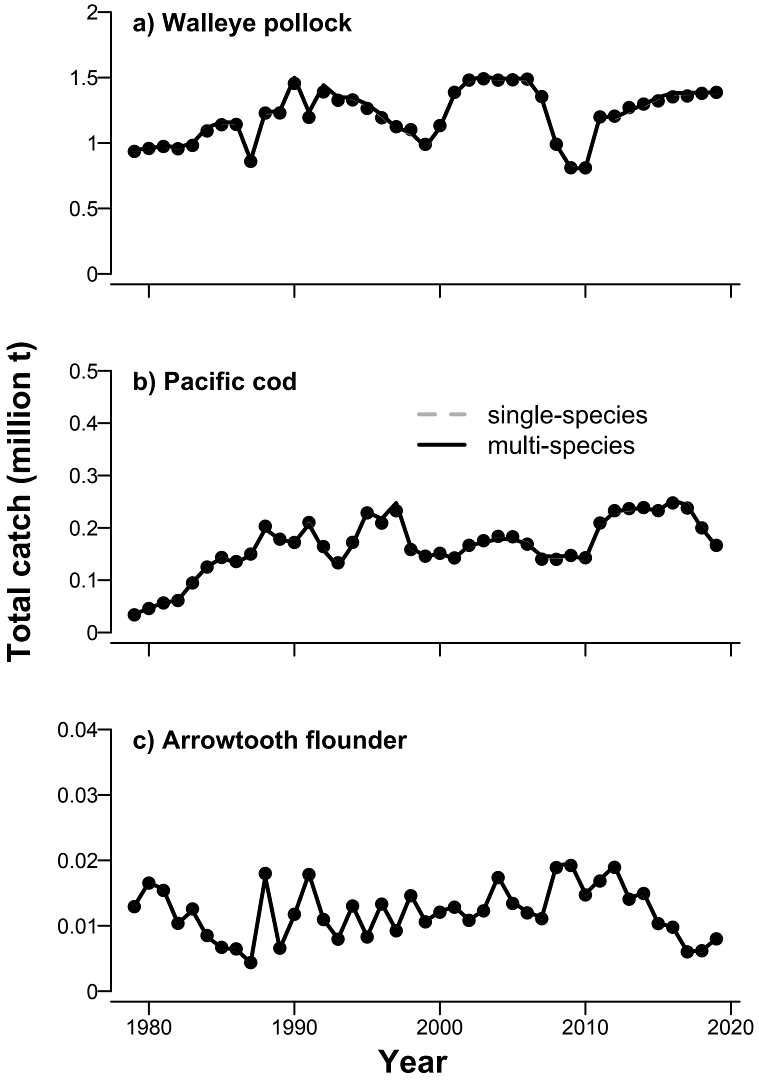

Figure 3: Total observed catch (circles) and model estimates of annual catch (lines) for single- and multi-species models (note that single species lines may not be visible as they overlap with multi-species estimates).

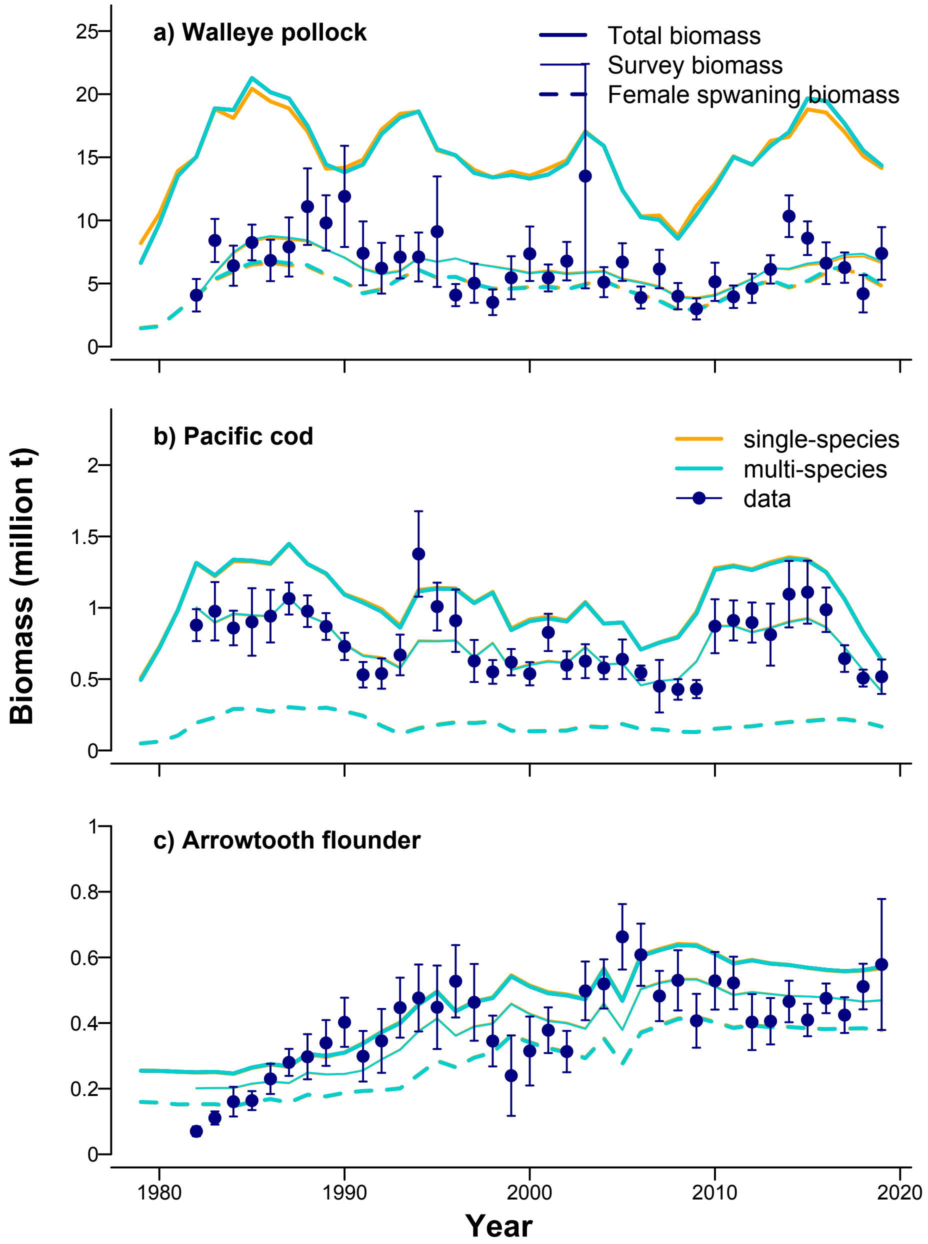

Figure 4: Single- (orange lines) and multi-species (blue lines) retrospective model estimates of total (thick solid lines), female spawning (dashed lines), and bottom-trawl survey biomass (thin solid lines). Filled circles represent mean observed groundfish survey biomass and standard errors of the mean (error bars).

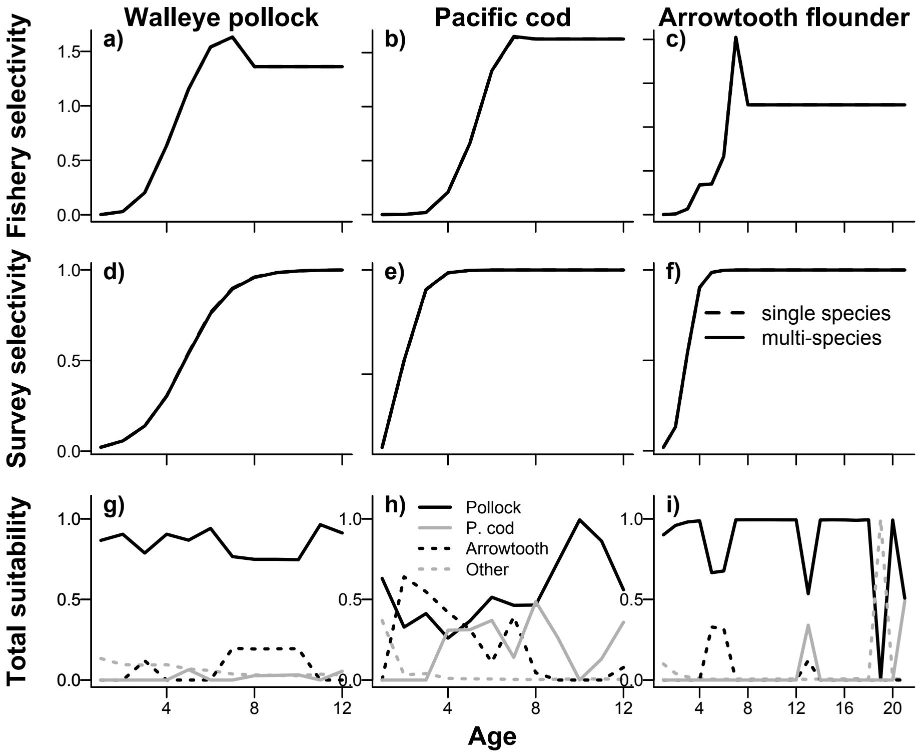

Figure 5: Single-species and multi-species fishery (first row; a-c) or survey selectivity (second row; d-f). Total suitability (across all prey species) for each predator age (third row; g-i).

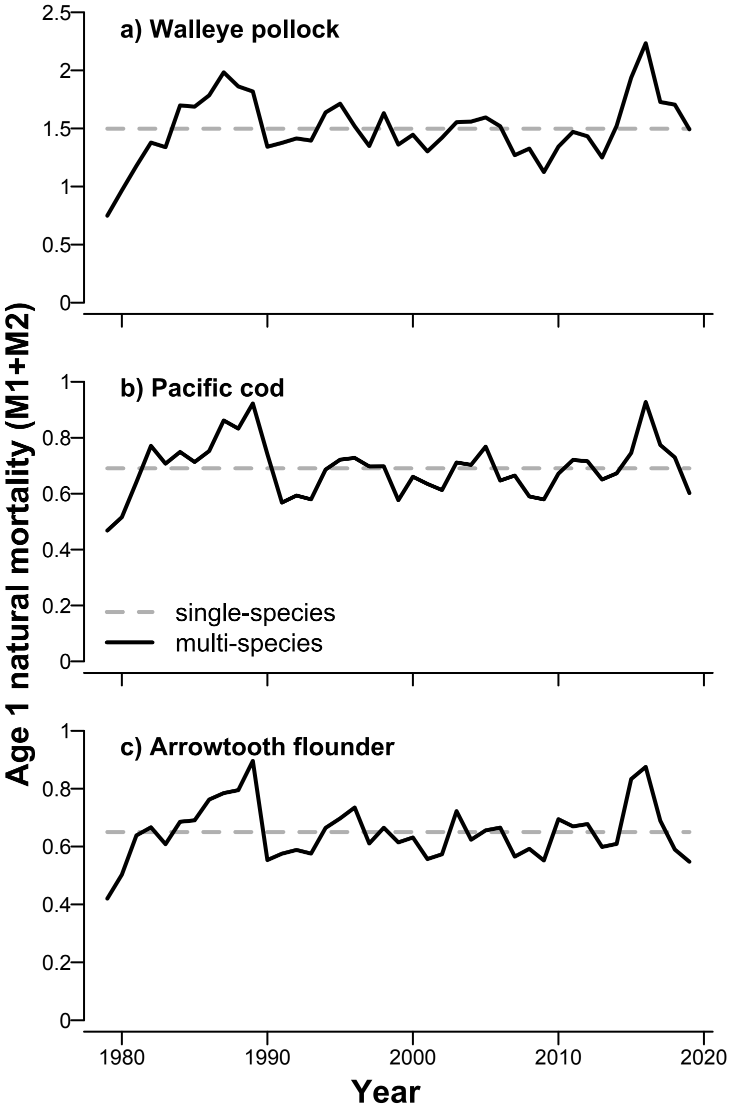

Figure 6: Annual variation in total mortality ($M1_{ij}$ $+M2_{ij,y}$) for age 1 pollock (a), Pacific cod (b), and arrowtooth flounder (c) from the single-species models (dashed line), multi-species models with temperature (black line).Note different Y-axis scales.

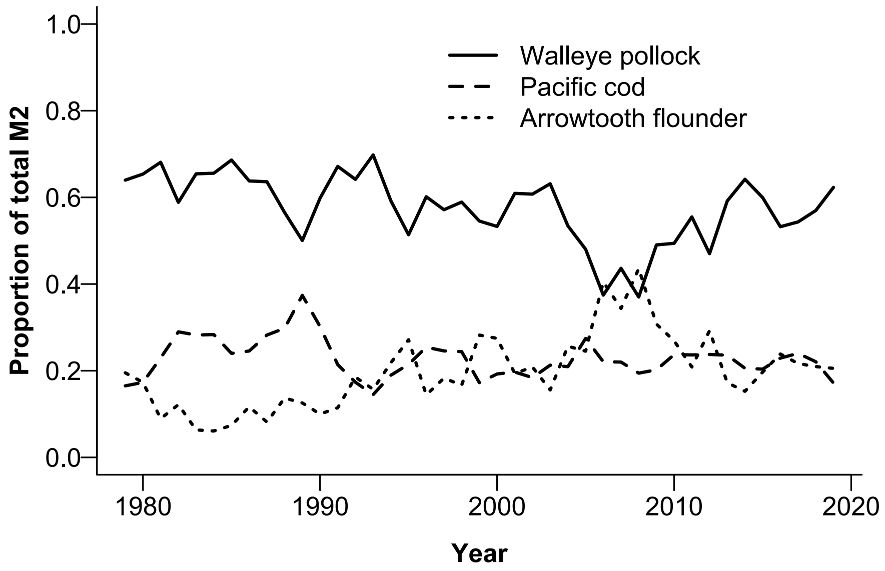

Figure 7: Proportion of total predation mortality for age 1 pollock from pollock (solid), Pacific cod (dashed), and arrowtooth flounder (dotted) predators across years.

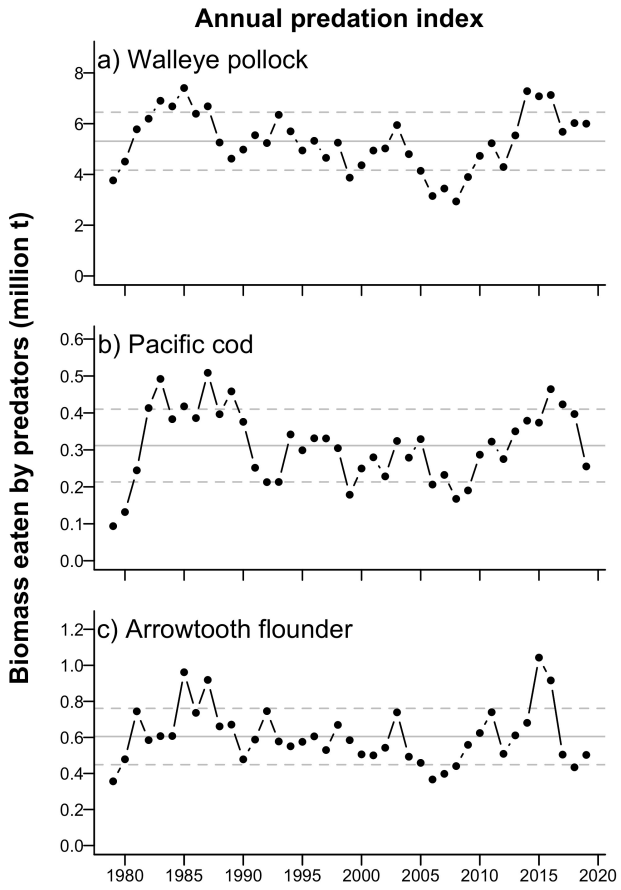

Figure 8: Multispecies estimates of prey species biomass consumed by all predators in the model a) total biomass of walleye pollock consumed by predators annually b) total biomass of P. cod consumed by predators annually, c) total biomass of arrowtooth flounder consumed by predators annually. Gray lines indicate 1979-2019 mean estimates for each species.

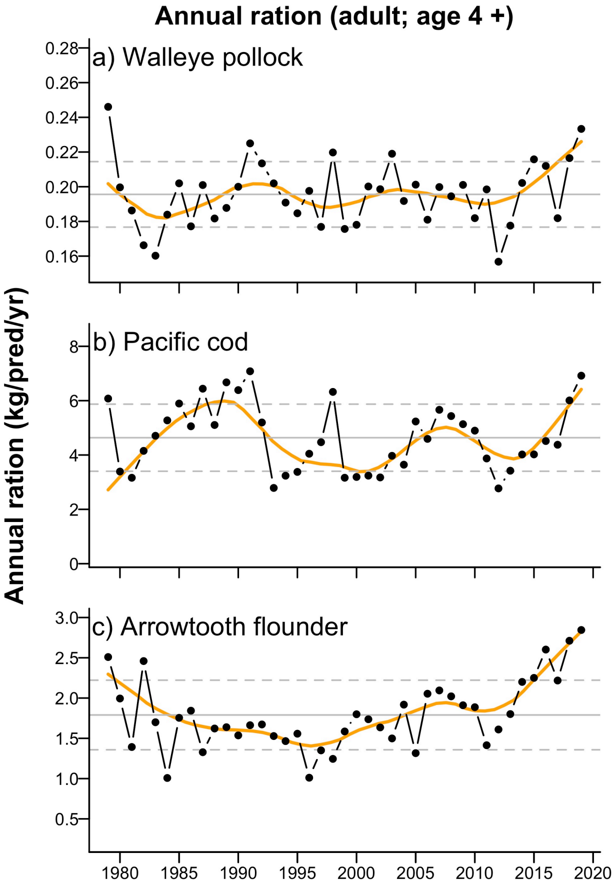

Figure 9: Multispecies estimates of annual ration (kg consumed per indiviudal per year) for adult (age 4 +) predators: a) pollock, b) P. cod, and c) arrowtooth flounder. Gray lines indicate 1979 -2019 mean estimates and 1 SD for each species; orange line is a 10 y (symmetric) loess polynomial smoother indicating trends in ration over time.

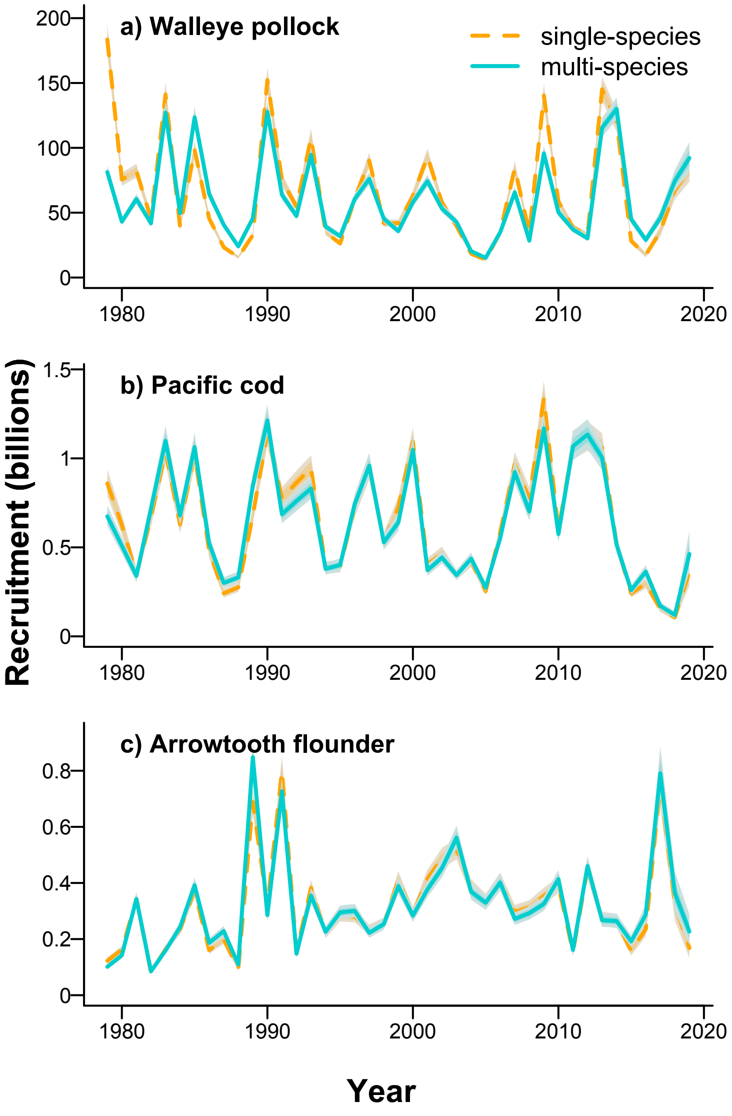

Figure 10: Annual single- and multi-species CEATTLE model estimates of recruitment (age 1) for pollock (a), Pacific cod (b), and arrowtooth flounder (c). Lighter shading represents the 95% CI around mean estimates. Darker shading represents +/- 1 standard error of the mean estimate.

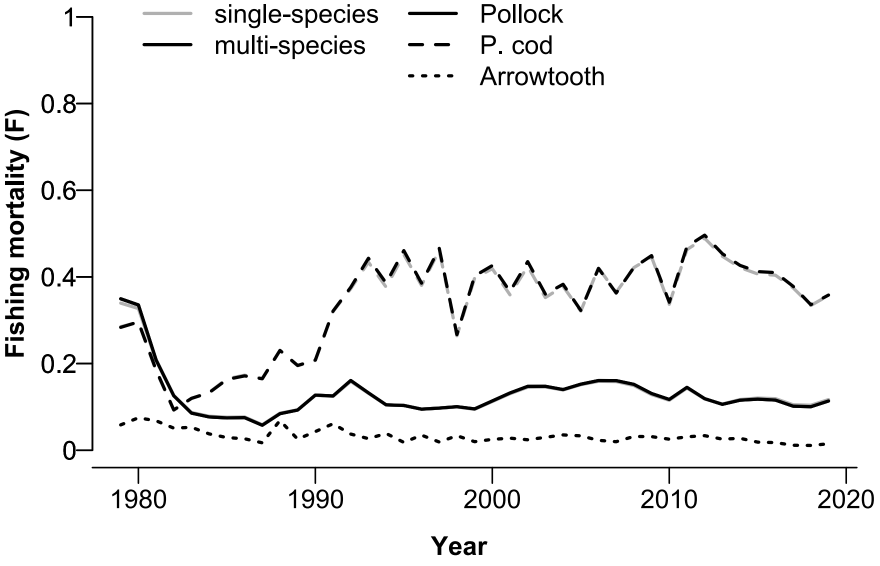

Figure 11: Timeseries of single- and multi-species (gray and black, respectively) CEATTLE model estimates of fishing mortality rate for eastern Being Sea walleye pollock (solid lines), Pacific cod (dashed lines), and arrowtooth flounder (dotted lines). Note that the single- and multi-species lines for arrowtooth flounder overlap.

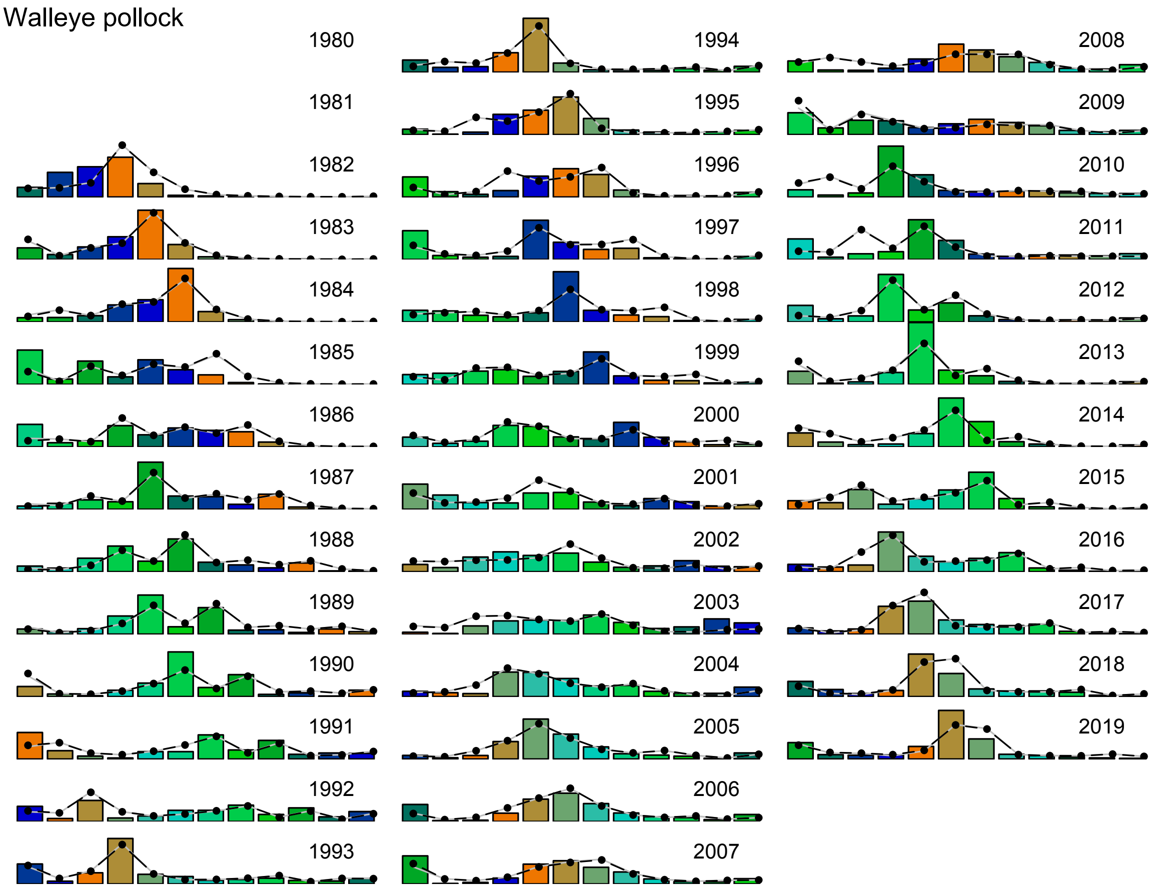

Figure 12: Survey age compostitions for walleye pollock. Bars represent observed values, black and gray points represent single- and multi-species fits to the data, respectively.

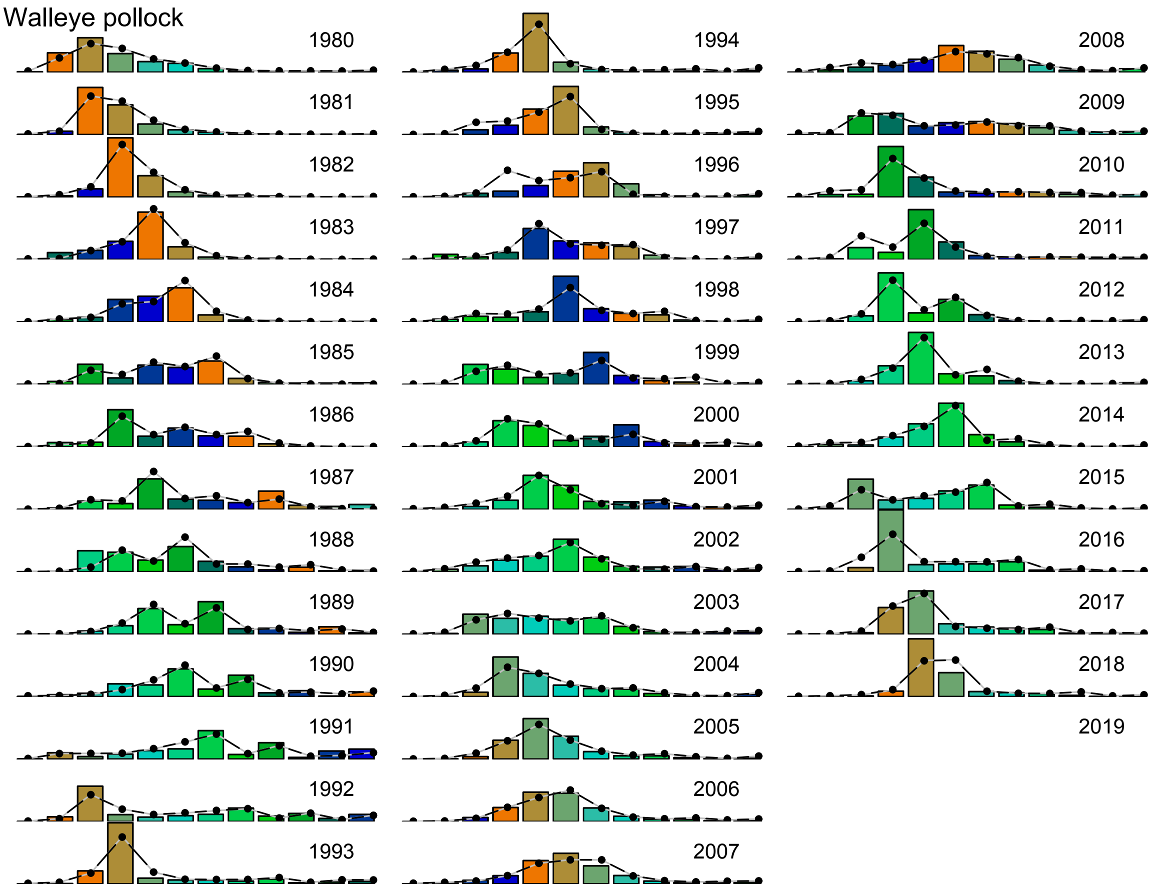

Figure 13: Fishery age compostitions for walleye pollock. Bars represent observed values, black and gray points represent single- and multi-species fits to the data, respectively.

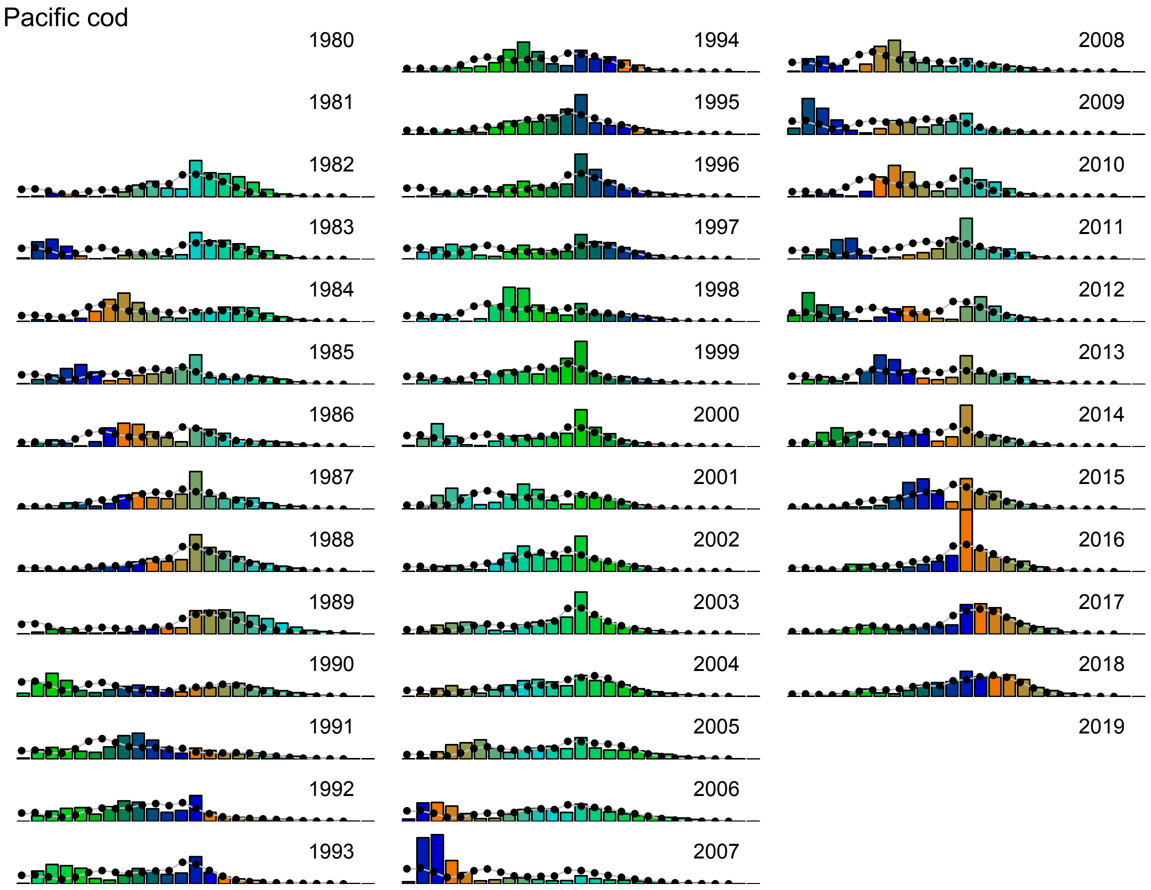

Figure 14: Survey length compostitions for Pacific cod Bars represent observed values, black and gray points represent single- and multi-species fits to the data, respectively.

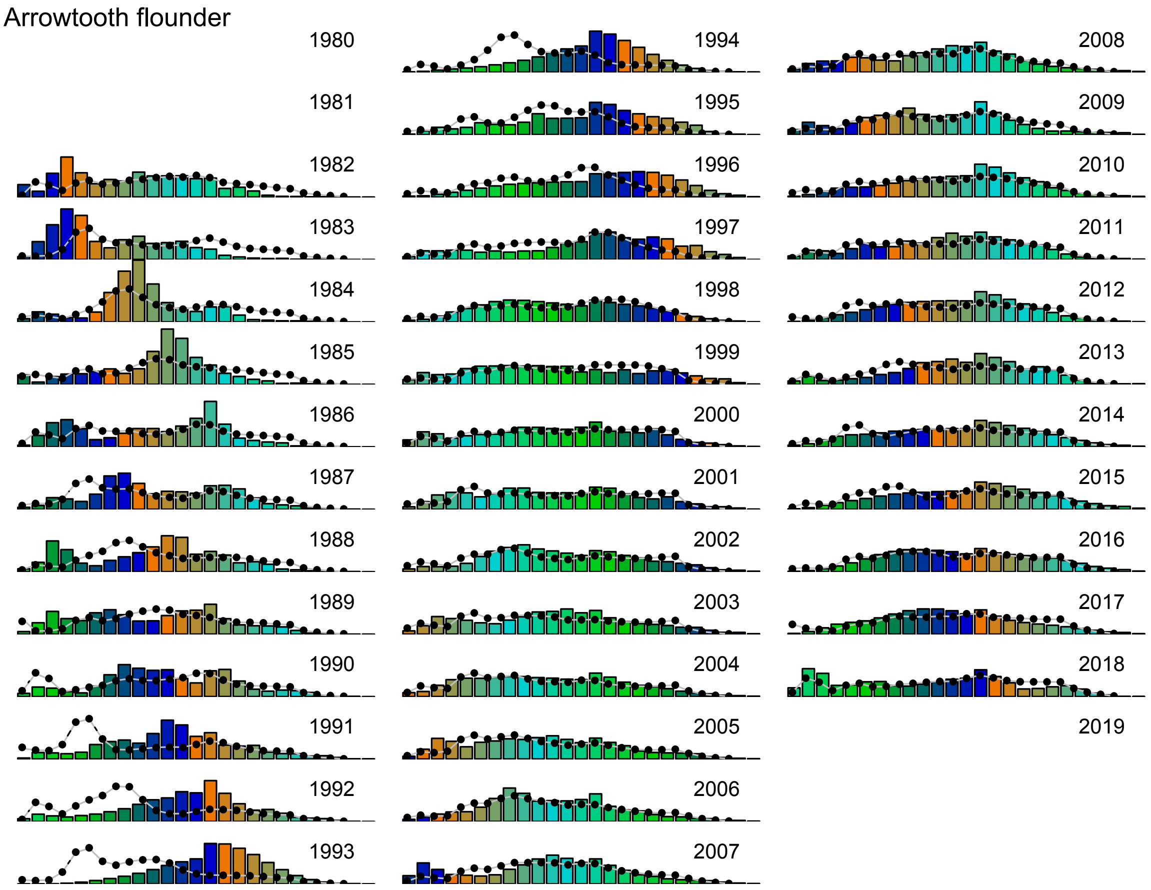

Figure 15: Survey length compostitions for arrowtooth flounder Bars represent observed values, black and gray points represent single- and multi-species fits to the data, respectively.

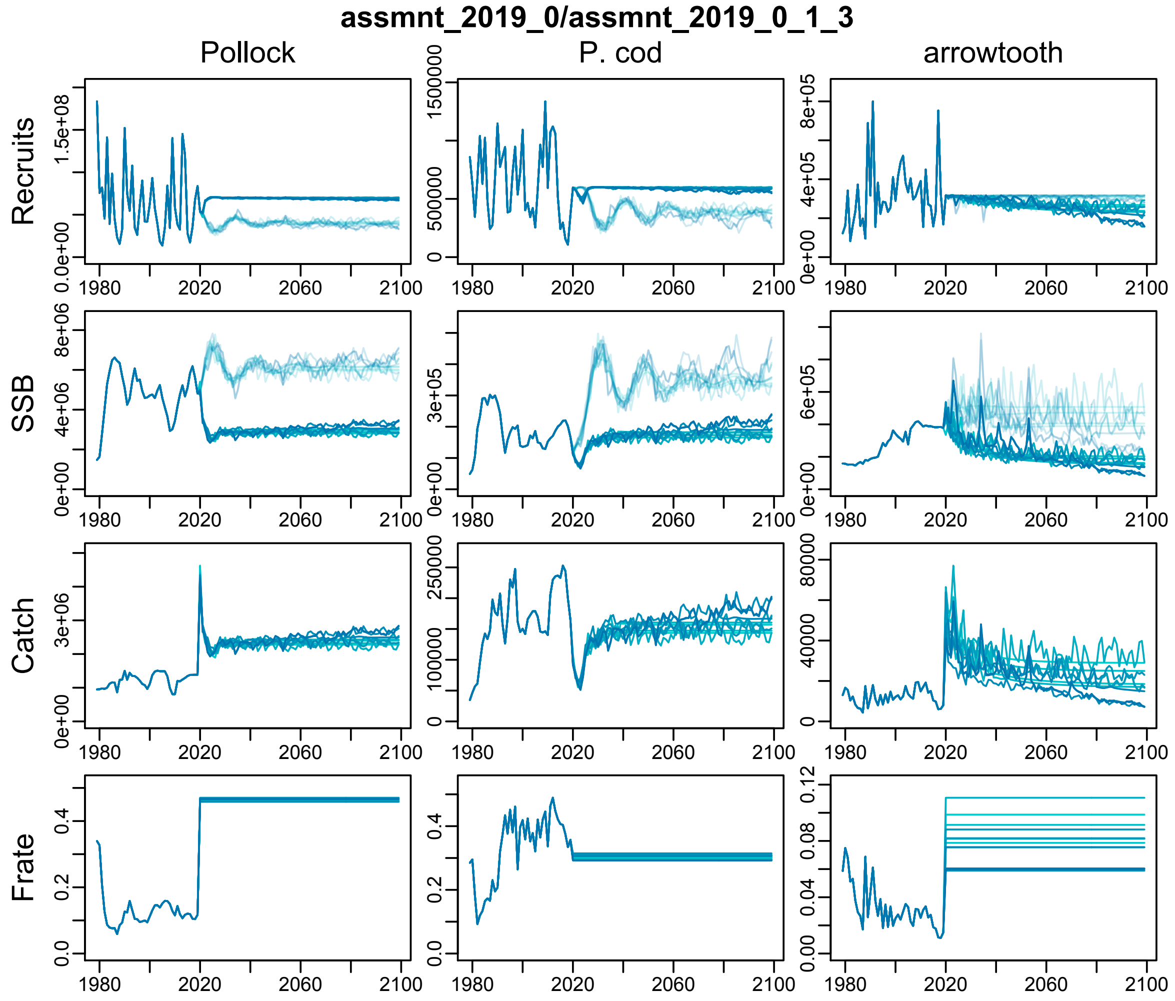

Figure 16: Single-species CEATTLE model projections of unfished (SSB_0; light shading) and fished spawning stock biomass at the harvest rate corresponding to the ABC proxy ($SSB_{40\%}$; darker shading) for each species. The lines represent different climate scenarios which impact weight at age and predation in the model.Only the constant scenario was used for ABC estimates.

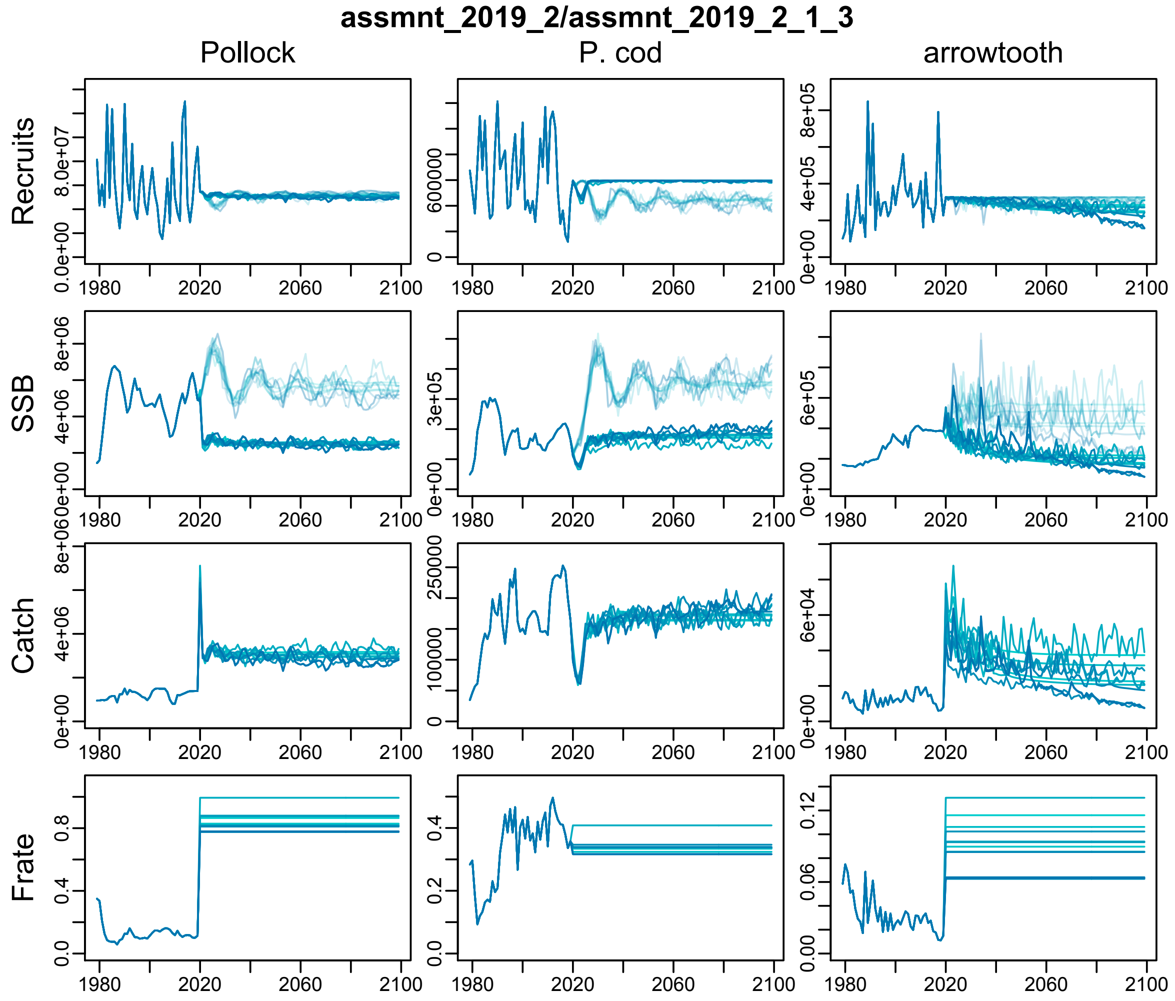

Figure 17: Multi-species CEATTLE model projections of unfished (SSB0; light shading) and fished spawning stock biomass at the harvest rate corresponding to the ABC proxy ($SSB_{40\%}$; darker shading) for each species. The lines represent different climate scenarios which impact weight at age and predation in the model. Only the constant scenario was used for ABC estimates.

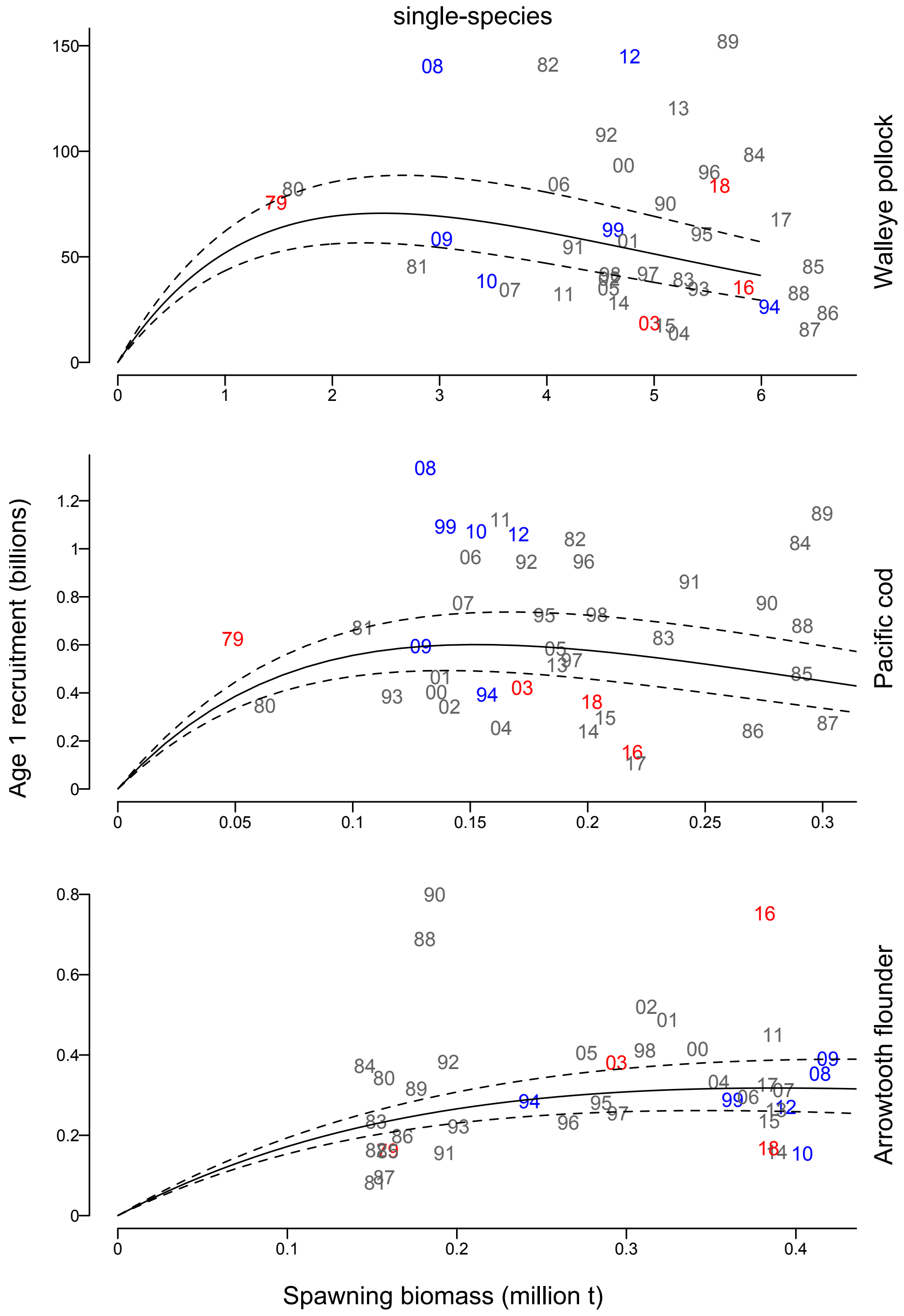

Figure 18: Stock-recruit curves for the single-species model. Red and blue text indicates years where bottom temperature was + or 1 standard deviation from the mean (respectively).

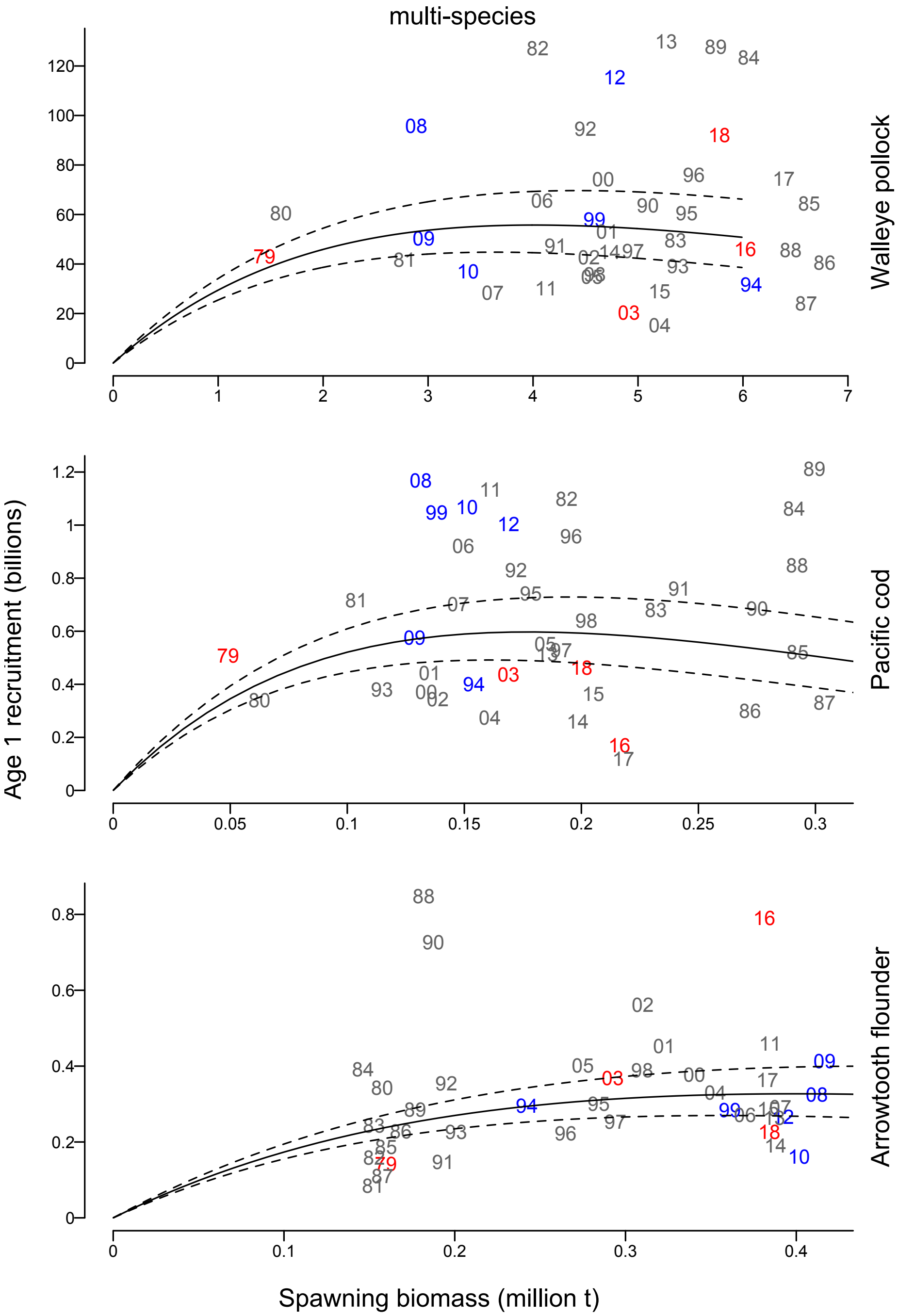

Figure 19: Stock-recruit curves for the multi-species model. Red and blue text indicates years where bottom temperature was + or 1 standard deviation from the mean (respectively).

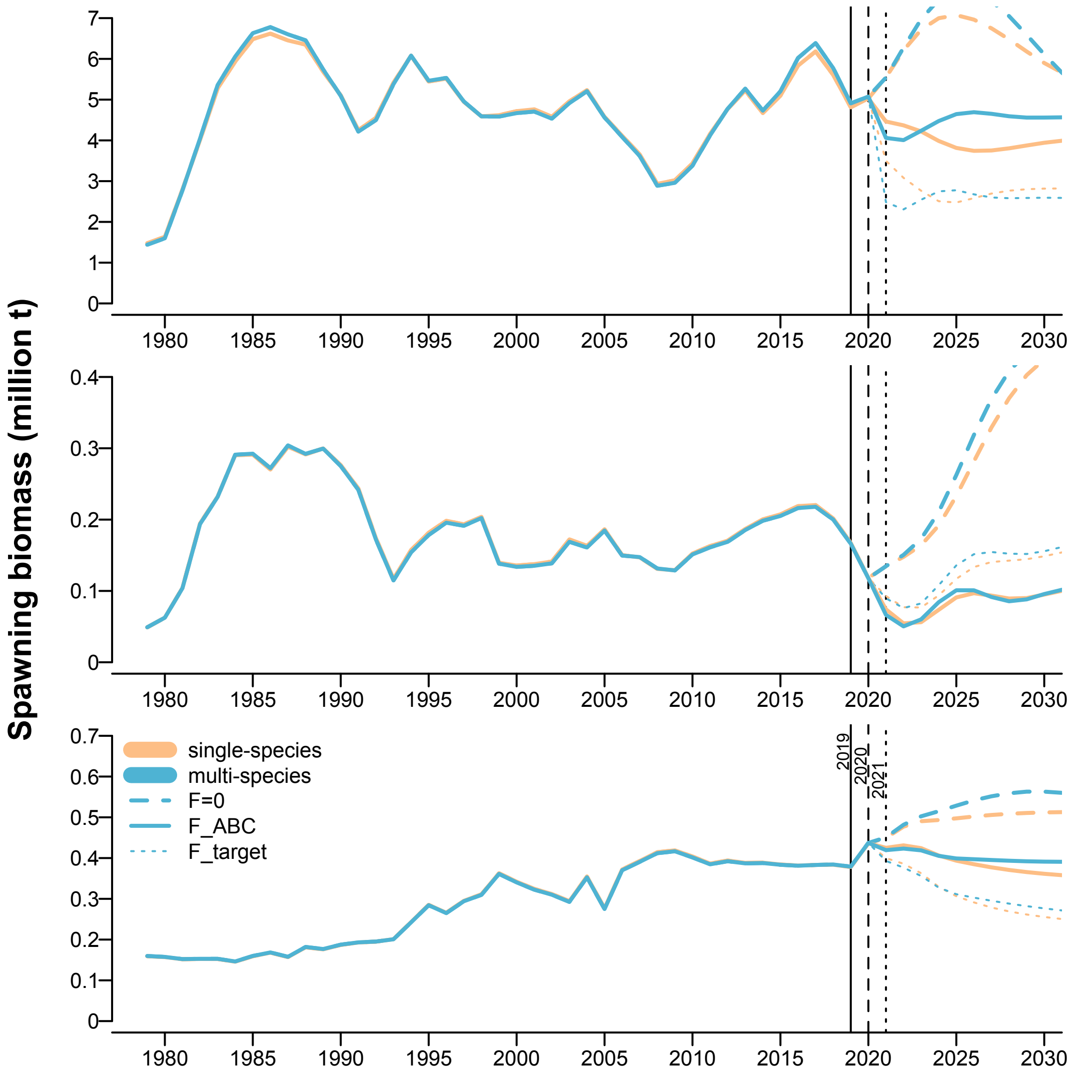

Figure 20: Spawning stock biomass for each species at the start of each model year. Vertical line represents end of 2019 start of 2020

	Walleye pollock	Pacific cod	Arrowtooth flounder
α	0.006	0.004	0.005
β	3.04	3.22	3.175
C_A	0.119	0.041	0.125
C_B	–0.46	–0.122	–0.199
C_Q	2.6	2.41	2.497
T_{C_o}	10	13.7	20.512
T_{C_M}	15	21	26
RFR_{juv}			
EBS	0.41	0.68	0.83
AI	0.24	0.48	0.52
GOA	0.49	0.41	0.79
RFR_{adult}			
EBS	0.79	1.57	1.29
AI	0.72	0.68	1.22
GOA	0.56	0.47	1.07
H			
EBS	11.798	8.691	4.651
AI	28.816	8.691	3.443
GOA	9.008	17.628	5.397
k			
EBS	0.375	0.511	1.235
AI	0.422	0.511	0.531
GOA	2.036	0.133	0.355
a_0			
EBS	0.357	–0.259	–1.495
AI	0.486	–0.259	–2
GOA	–0.445	0.115	–0.618
d			
EBS	0.548	0.715	0.835
AI	0.428	0.715	0.776
GOA	0.796	0.544	0.673
H_{spec}			
EBS	8.214	10.176	6.392
AI	11.869	12.468	4.583
GOA	11.669	10.613	5.502
k_{spec}			
EBS	0.672	0.33	0.405
AI	1.05	0.42	0.269
GOA	1.001	0.414	0.341
$a_{0\text{spec}}$			
EBS	0.025	–0.06	–0.049
AI	–0.018	–0.029	–0.84
GOA	0.078	–0.275	–0.577

Figure 21: Table 6. Temperature-dependent Von Bertalanffy parameter (parm) estimates, standard deviation in parameter estimates (stdev), and confidence intervals (CI).
