# Supplementary figures and images for: Ecosystem-based fisheries management forestalls climate-driven collapse
Source: Nat Commun. 2020 Sep 11;11:4579. doi: 10.1038/s41467-020-18300-3 (PMC7486947; doi:10.1038/s41467-020-18300-3)

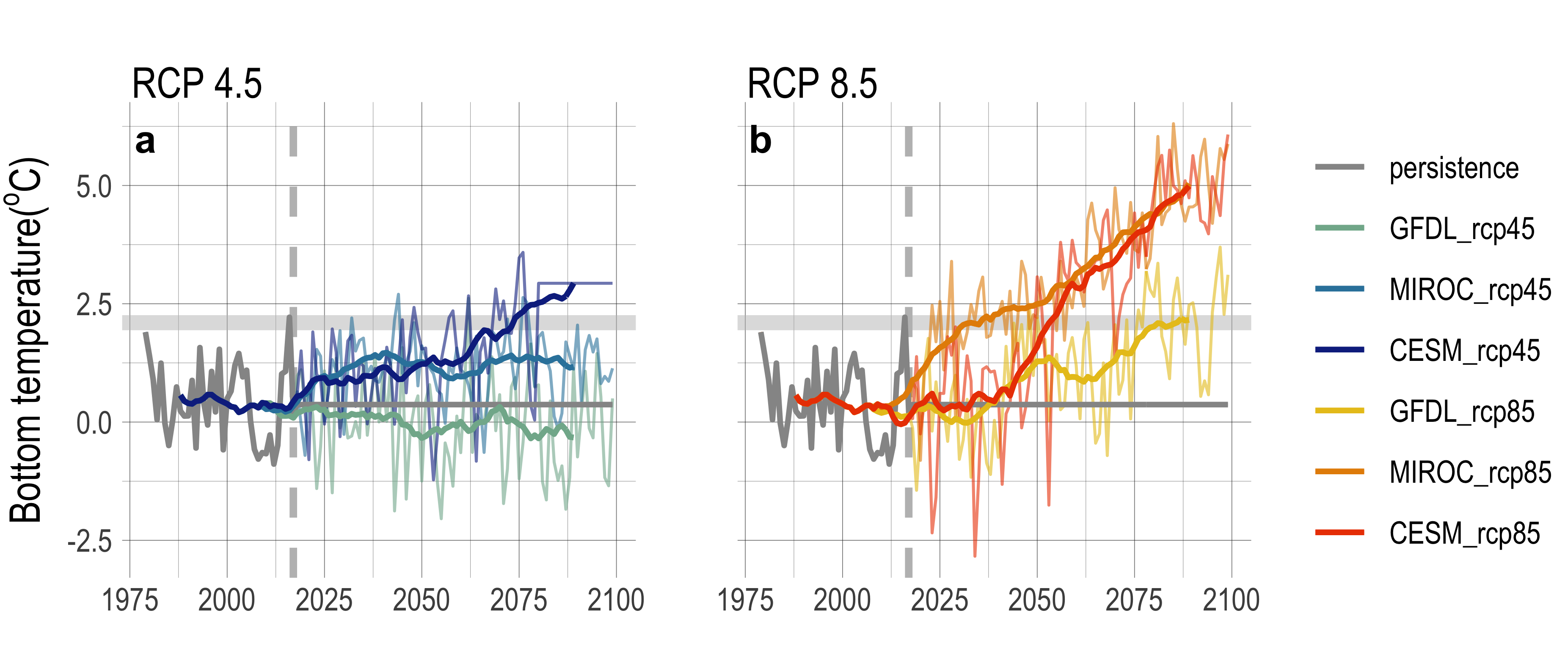

Supplement: Supplementary file 4 — Supplementary Software [file 41467_2020_18300_MOESM4_ESM.zip › Supplementary_Software/EBM_Holsman_NatComm-master/Figures/Fig2.tiff]

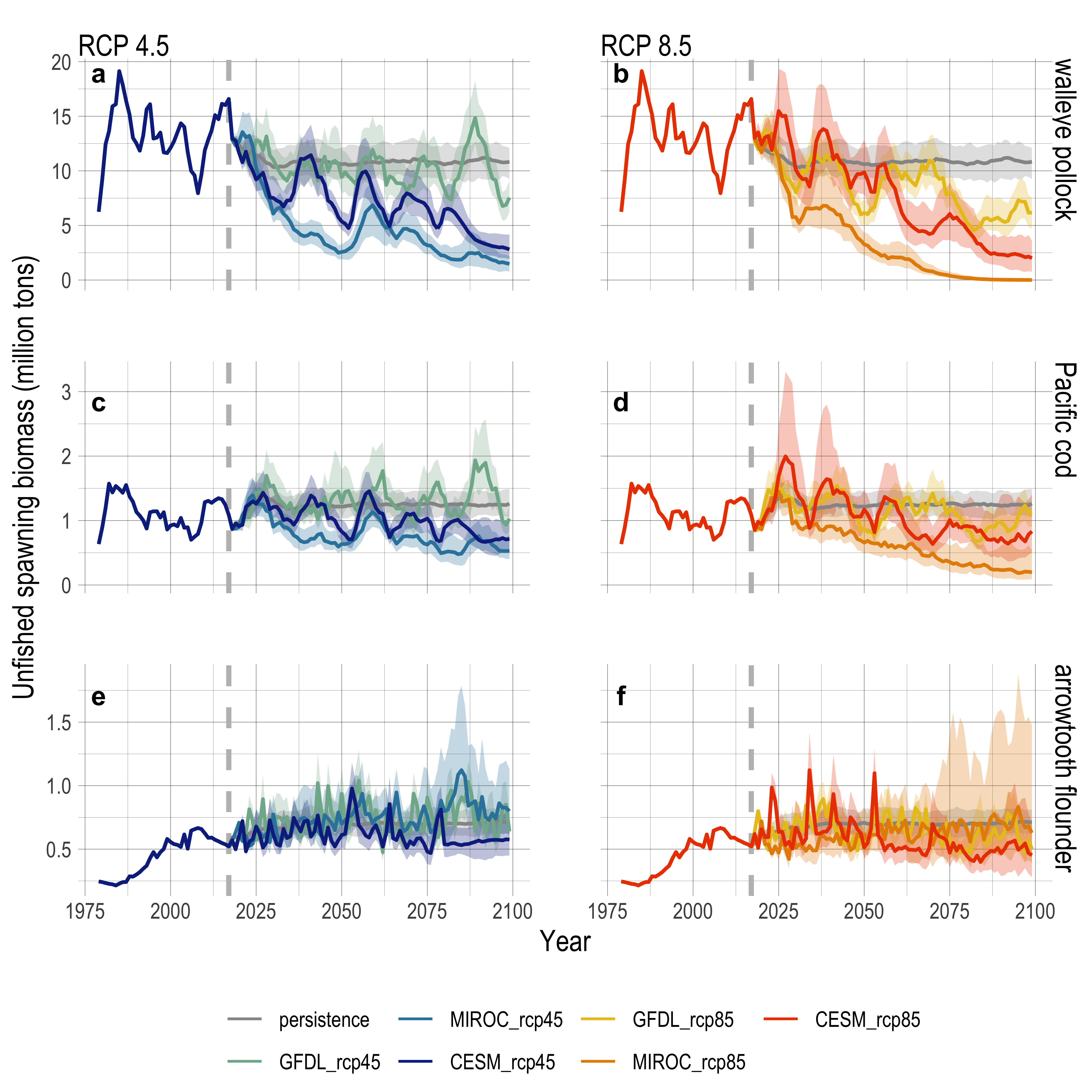

Supplement: Supplementary file 4 — Supplementary Software [file 41467_2020_18300_MOESM4_ESM.zip › Supplementary_Software/EBM_Holsman_NatComm-master/Figures/Fig3.tiff]

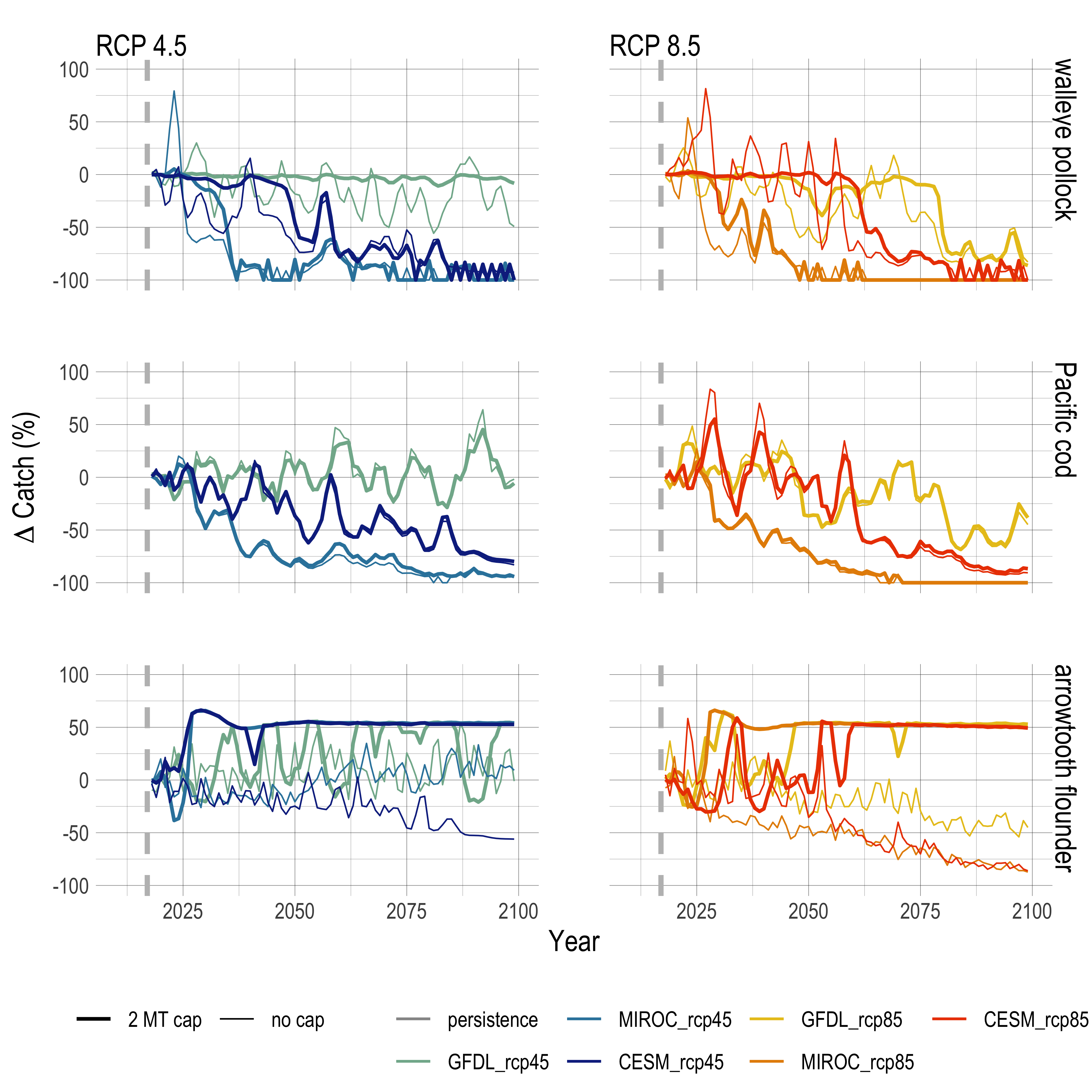

Supplement: Supplementary file 4 — Supplementary Software [file 41467_2020_18300_MOESM4_ESM.zip › Supplementary_Software/EBM_Holsman_NatComm-master/Figures/Fig4.tiff]

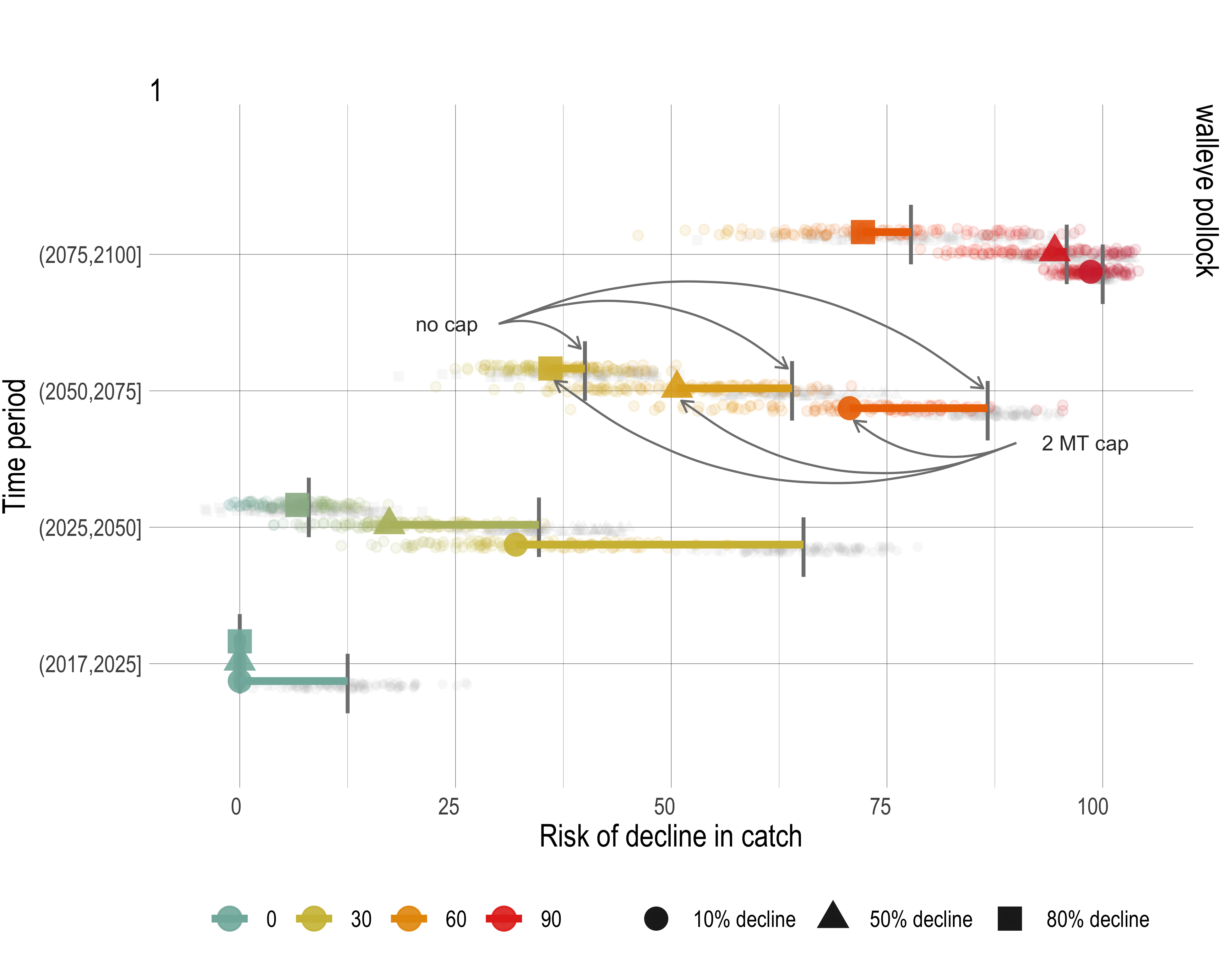

Supplement: Supplementary file 4 — Supplementary Software [file 41467_2020_18300_MOESM4_ESM.zip › Supplementary_Software/EBM_Holsman_NatComm-master/Figures/Fig5.tiff]

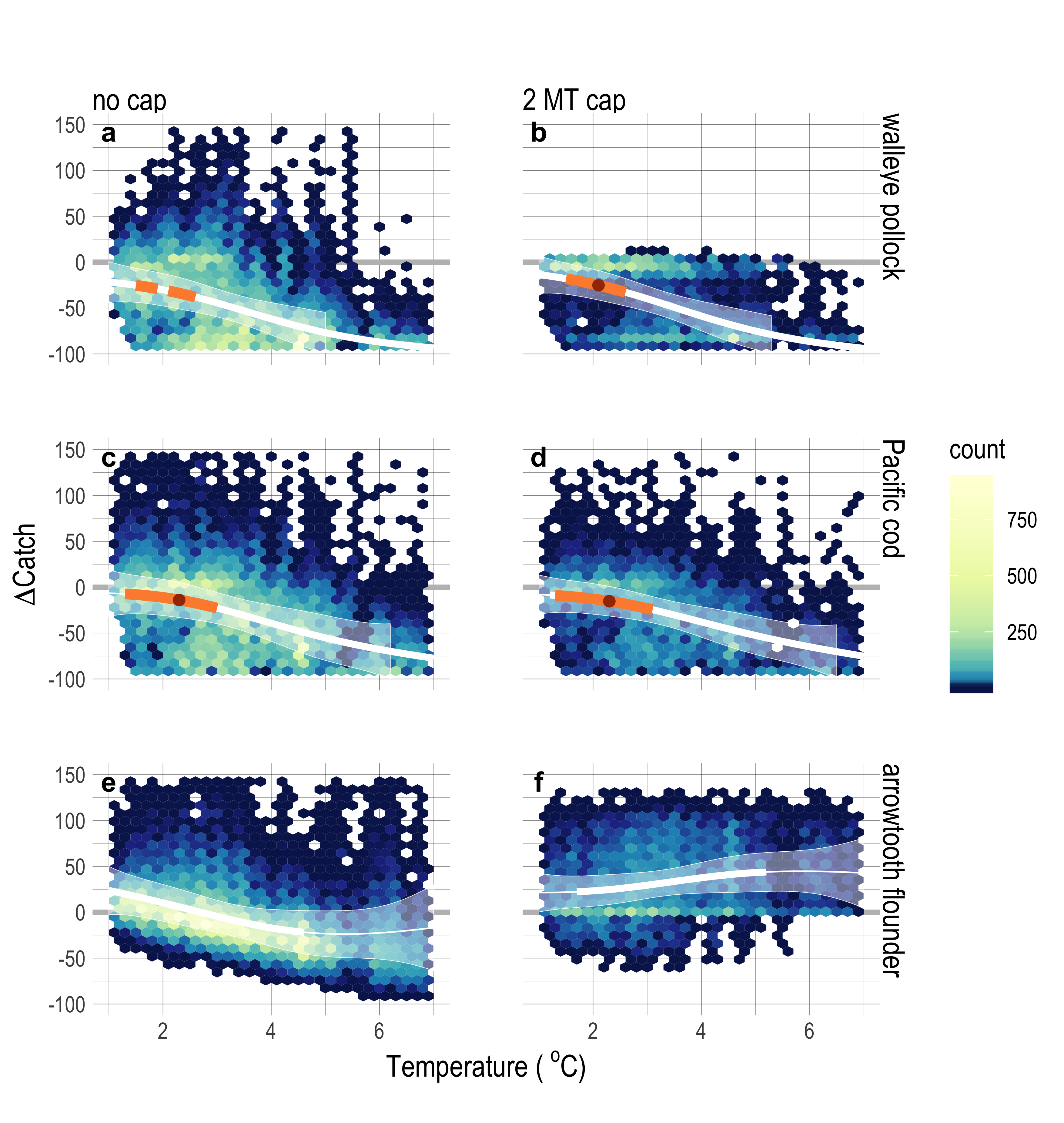

Supplement: Supplementary file 4 — Supplementary Software [file 41467_2020_18300_MOESM4_ESM.zip › Supplementary_Software/EBM_Holsman_NatComm-master/Figures/Fig6.tiff]

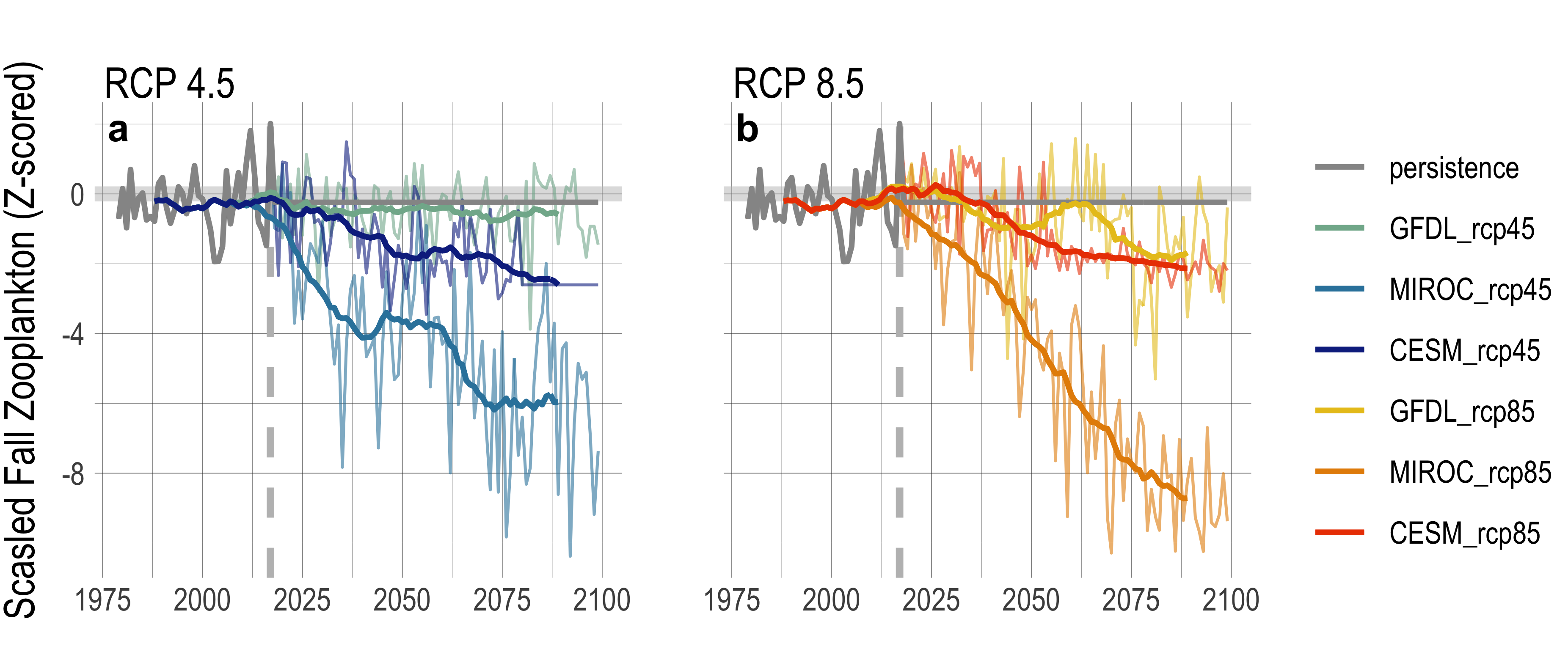

Supplement: Supplementary file 4 — Supplementary Software [file 41467_2020_18300_MOESM4_ESM.zip › Supplementary_Software/EBM_Holsman_NatComm-master/Figures/FigS1.tiff]

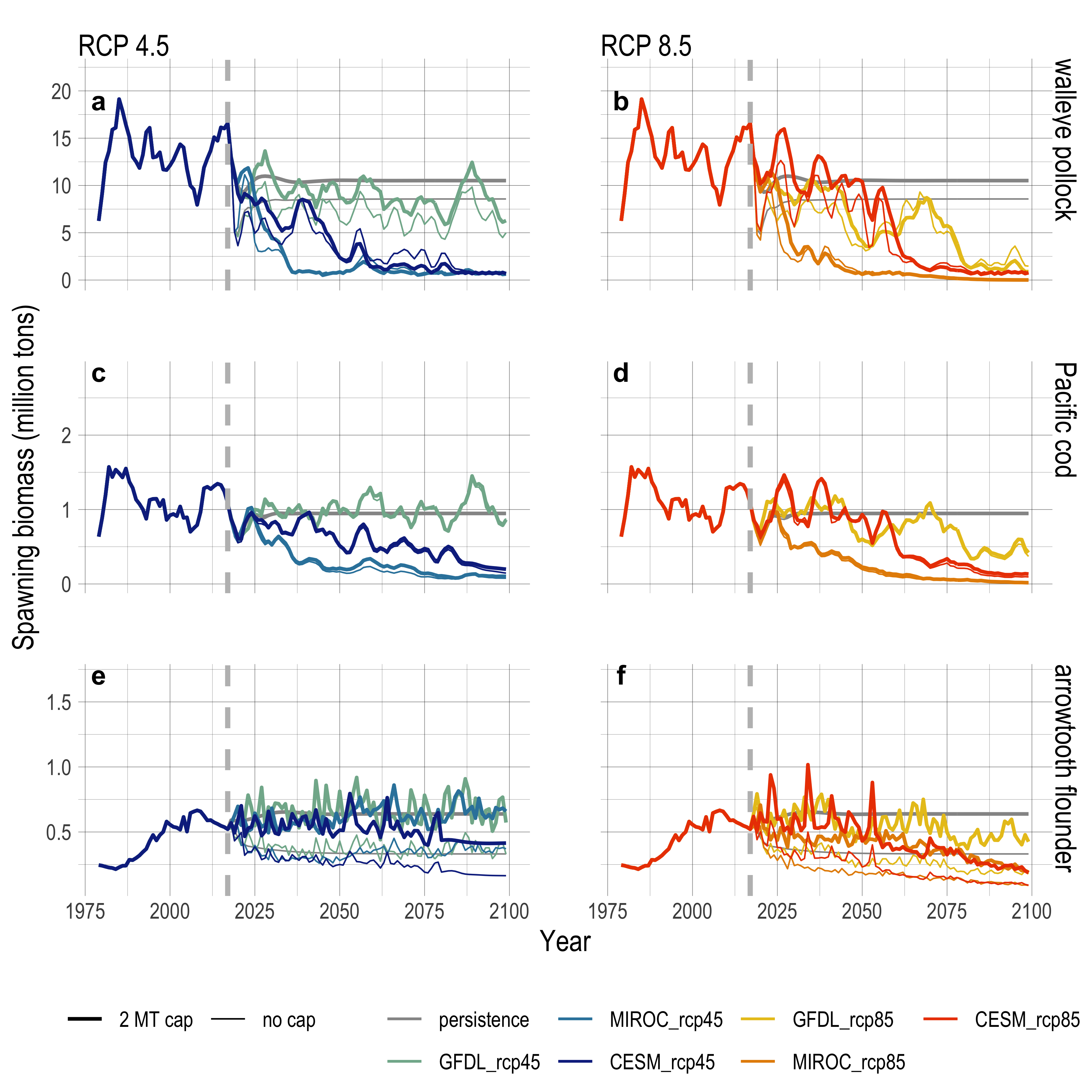

Supplement: Supplementary file 4 — Supplementary Software [file 41467_2020_18300_MOESM4_ESM.zip › Supplementary_Software/EBM_Holsman_NatComm-master/Figures/FigS2.tiff]

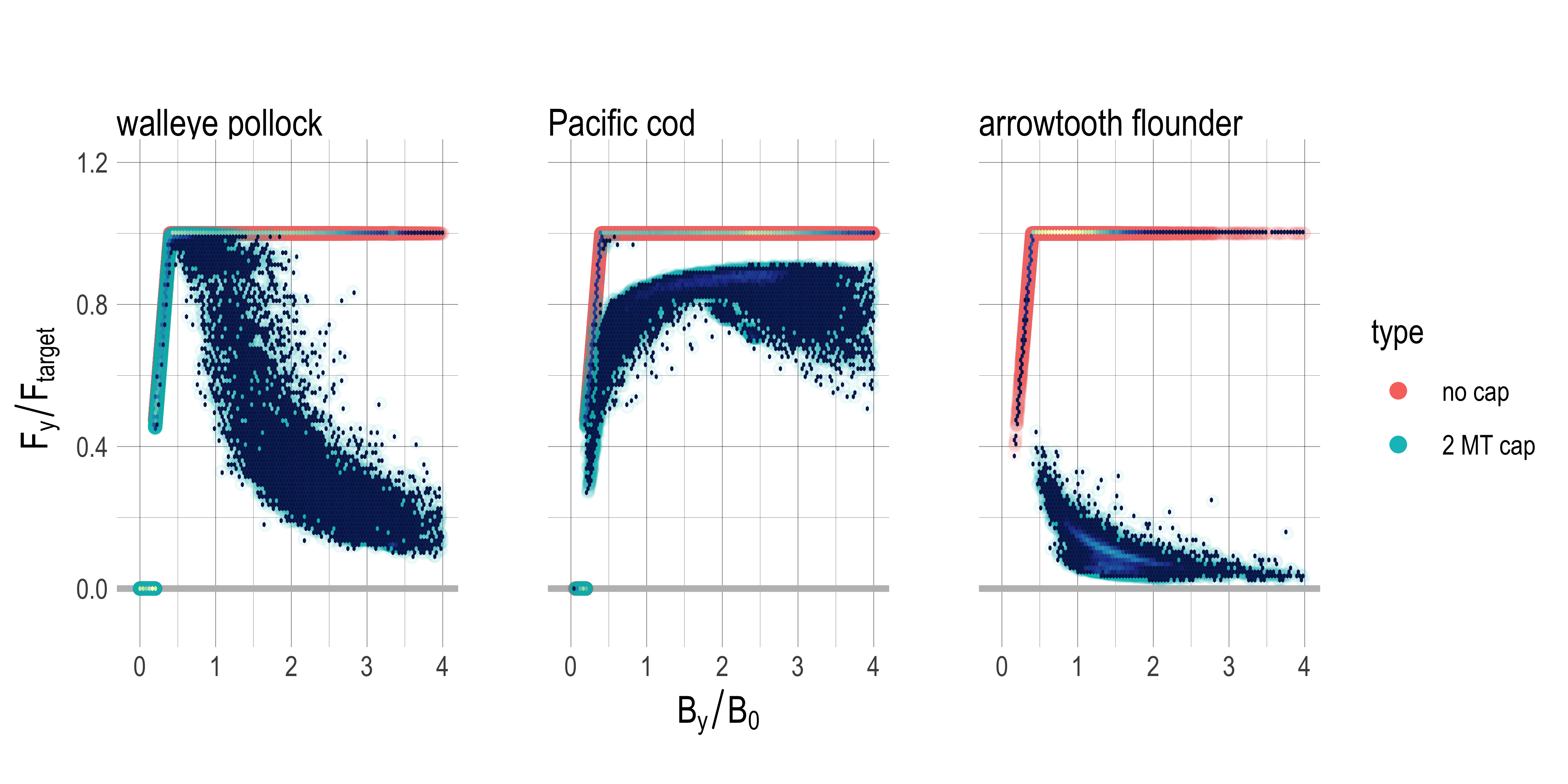

Supplement: Supplementary file 4 — Supplementary Software [file 41467_2020_18300_MOESM4_ESM.zip › Supplementary_Software/EBM_Holsman_NatComm-master/Figures/FigS3.tiff]

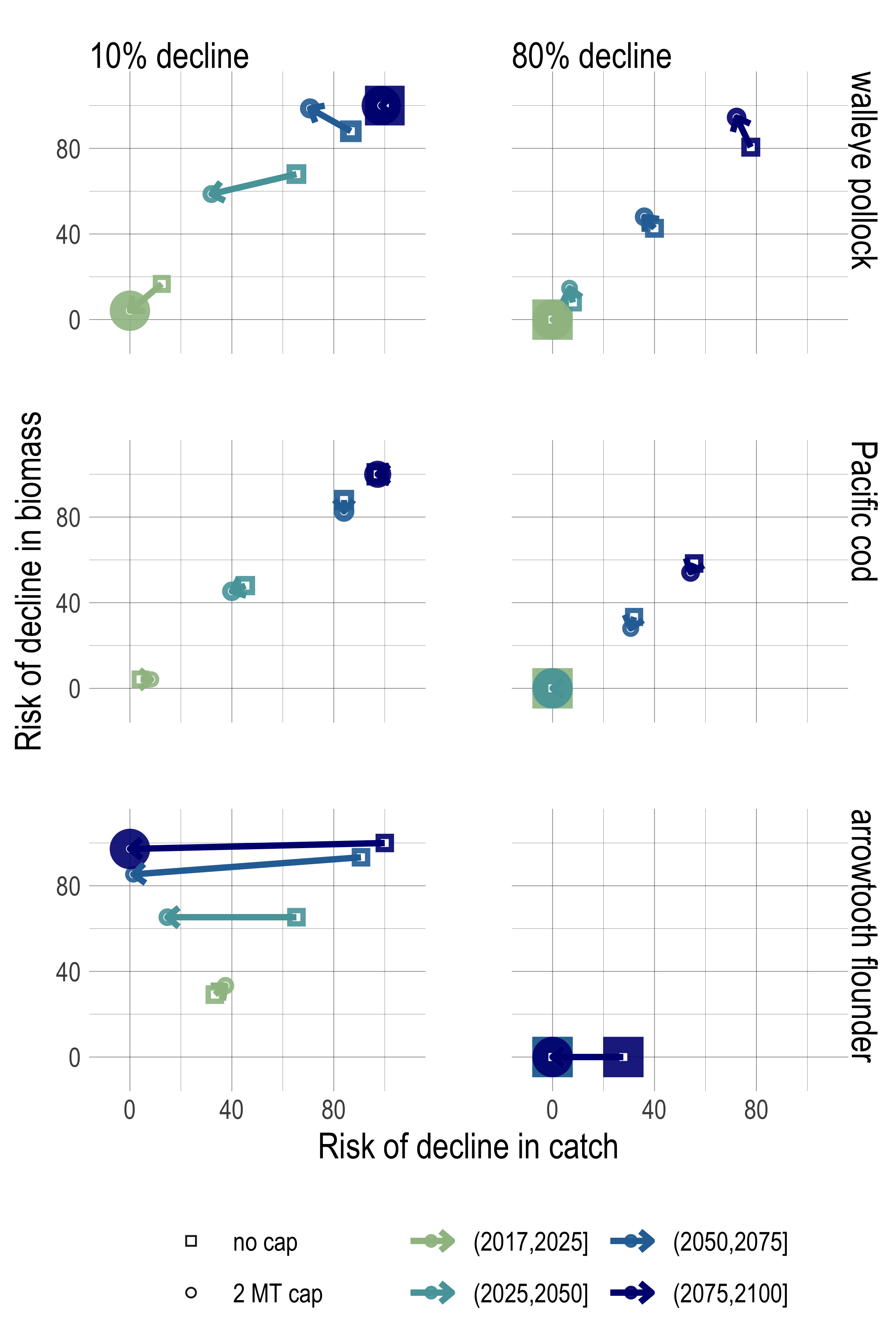

Supplement: Supplementary file 4 — Supplementary Software [file 41467_2020_18300_MOESM4_ESM.zip › Supplementary_Software/EBM_Holsman_NatComm-master/Figures/FigS4.tiff]

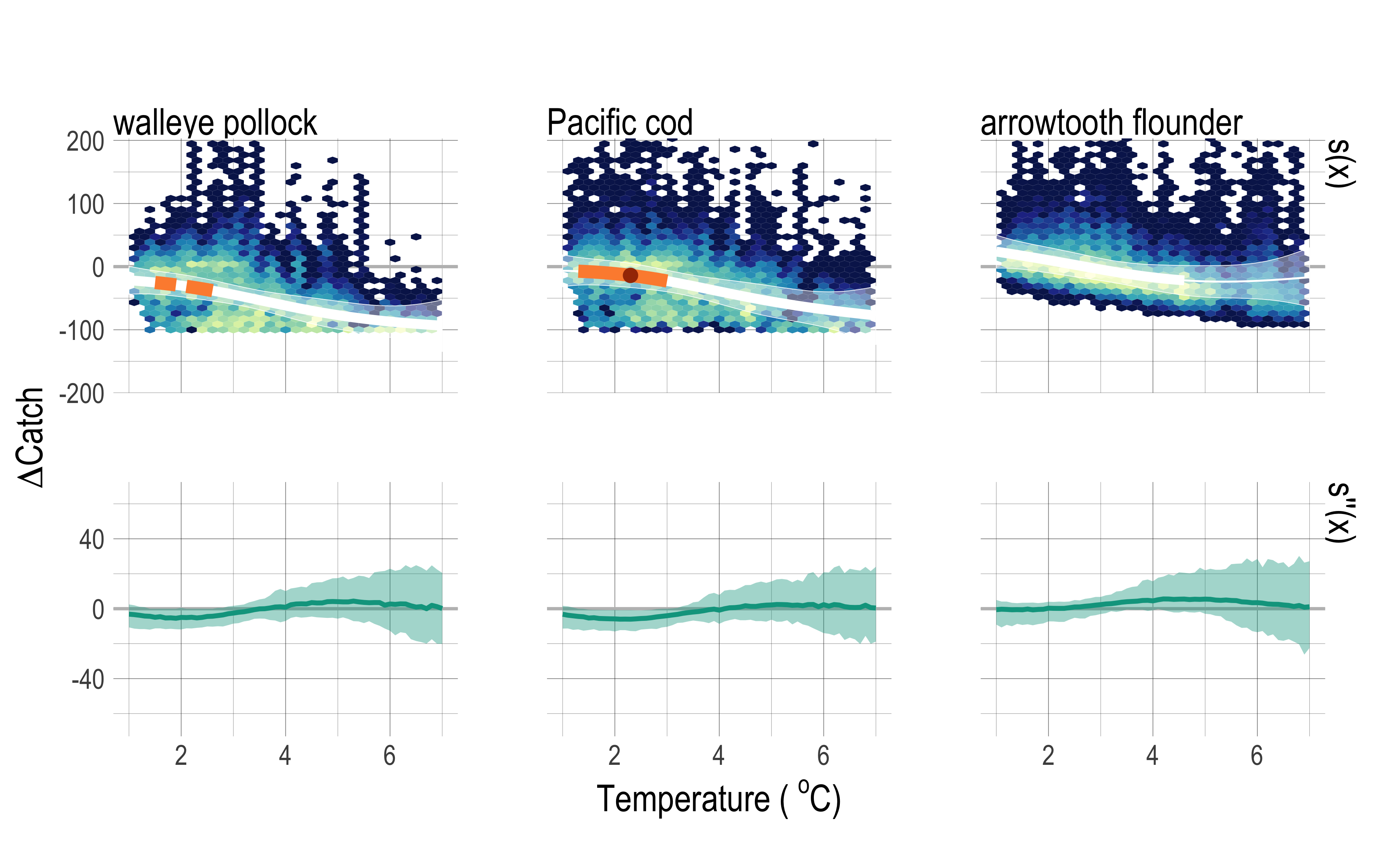

Supplement: Supplementary file 4 — Supplementary Software [file 41467_2020_18300_MOESM4_ESM.zip › Supplementary_Software/EBM_Holsman_NatComm-master/Figures/FigS5.tiff]
