# Supplementary material for: Ecosystem-based fisheries management forestalls climate-driven collapse
Source: Nat Commun. 2020 Sep 11;11:4579. doi: 10.1038/s41467-020-18300-3 (PMC7486947; doi:10.1038/s41467-020-18300-3)
Supplement: Supplementary file 5 — Reporting Summary [file 41467_2020_18300_MOESM5_ESM.pdf]

nature research

Corresponding author(s): Dr. Kirstin K. Holsman

Last updated by author(s): Jul 30, 2020

Reporting Summary

Nature Research wishes to improve the reproducibility of the work that we publish. This form provides structure for consistency and transparency in reporting. For further information on Nature Research policies, see Authors & Referees and the Editorial Policy Checklist .

Statistics

For all statistical analyses, confirm that the following items are present in the figure legend, table legend, main text, or Methods section.

n/a	Confirmed	
[ ]	[x]	The exact sample size (*n*) for each experimental group/condition, given as a discrete number and unit of measurement
[ ]	[x]	A statement on whether measurements were taken from distinct samples or whether the same sample was measured repeatedly
[ ]	[x]	The statistical test(s) used AND whether they are one- or two-sided<br>*Only common tests should be described solely by name; describe more complex techniques in the Methods section.*
[ ]	[x]	A description of all covariates tested
[ ]	[x]	A description of any assumptions or corrections, such as tests of normality and adjustment for multiple comparisons
[ ]	[x]	A full description of the statistical parameters including central tendency (e.g. means) or other basic estimates (e.g. regression coefficient) AND variation (e.g. standard deviation) or associated estimates of uncertainty (e.g. confidence intervals)
[x]	[ ]	For null hypothesis testing, the test statistic (e.g. *F*, *t*, *r*) with confidence intervals, effect sizes, degrees of freedom and *P* value noted<br>*Give P values as exact values whenever suitable.*
[x]	[ ]	For Bayesian analysis, information on the choice of priors and Markov chain Monte Carlo settings
[ ]	[x]	For hierarchical and complex designs, identification of the appropriate level for tests and full reporting of outcomes
[ ]	[x]	Estimates of effect sizes (e.g. Cohen's *d*, Pearson's *r*), indicating how they were calculated

Our web collection on statistics for biologists contains articles on many of the points above.

Software and code

Policy information about availability of computer code

Data collection	All code used to generate intermediate and final data is available at https://github.com/kholsman/EBM_Holsman_NatComm. Archived data sets (including multispecies assessment model simulations) are large (>10GB) and are available temporarily by request to kirstin.holsman@noaa.gov but will be archived and made publicly accessible through figshare.com at the time of publication. Assessment data includes NOAA Alaska Fisheries Science Center groundfish data collected annually in the Bering Sea and available in data_input_files included in the github repository and also accessible by request from the authors. The CEATTLE and recruitment models are programed in AD Model Builder release version 11.6 (http://www.admb-project.org); the ROMSNPZ model is programmed in Regional Ocean Modeling System version 3.2; socioeconomic ATTACHv1.1.0 model (https://github.com/amandafaig/catchfunction), AIC and threshold analyses were conducted with R version 3.5.3 (2019-03-11) https://www.r-project.org using the mgcv package version 1.8-31.
Data analysis	Custom code was created for the multispecies stock assessment model (CEATTLE), recruitment model and projections, ROMSNPZ CMIP5 projections, and threshold analyses and plotting. Details can be found in Holsman et al, 2016, 2019, Hermann et al. 2019. All code is publicly available at the following github site and will be archived via Zenodo upon publication: https://github.com/kholsman/EBM_Holsman_NatComm.

For manuscripts utilizing custom algorithms or software that are central to the research but not yet described in published literature, software must be made available to editors/reviewers. We strongly encourage code deposition in a community repository (e.g. GitHub). See the Nature Research guidelines for submitting code & software for further information.

Data

Policy information about availability of data

All manuscripts must include a data availability statement. This statement should provide the following information, where applicable:

- Accession codes, unique identifiers, or web links for publicly available datasets
- A list of figures that have associated raw data
- A description of any restrictions on data availability

All data is available in the main text, the Supplementary Information or at the following Data Archive https://figshare.com/s/81007e2dd5edee0a5a7a (doi: 10.6084/

m9.figshare.12568625).

Field-specific reporting

Please select the one below that is the best fit for your research. If you are not sure, read the appropriate sections before making your selection.

☐ Life sciences ☐ Behavioural & social sciences ☒ Ecological, evolutionary & environmental sciences

For a reference copy of the document with all sections, see nature.com/documents/nr-reporting-summary-flat.pdf

Ecological, evolutionary & environmental sciences study design

All studies must disclose on these points even when the disclosure is negative.

Study description

Here we present results of management strategy evaluations specifically aimed at assessing the scope of adaptation through Ecosystem based management (EBM) for the nation's largest fisheries. Specifically, we used an ensemble of high-resolution coupled climate, ecosystem, fishery, and socio-economic models as part of the ongoing multidisciplinary Alaska Climate Integrated Modeling project (please see uploaded Hollowed et al., 2020) to quantify ecosystem-based management (EBM) reductions in climate-change risk to these key fisheries.

Research sample

Region: The Eastern Bering Sea of the United states EEZ. This region supports the largest fisheries in the US and is also increasingly impacted by loss of sea ice and climate-driven change. Since 2000 it is also a region that has successfully employed a number of ecosystem based management approaches including an ecosystem-based 2 million ton limit on total annual grounfish removals.

ROMSNPZ:
a) one high resolution hindcast of ocean conditions and lower-trophic level biomass :1979-2017
b) six high resolution projections of ocean conditions and lower-trophic level biomass : 2006-2100
 * 2 representative concentration pathways: AR5 CMIP5 RCP 4.5 and 8.5
 * 3 global climate models : GFDL-ESM2M, NCAR CESM, MIROC ESM
c) one climate-constant persistence scenario (average of 2006-2017 conditions); 2017-2100

Population dynamics; annual timestep; Eastern Bering Sea shelf US stocks; 3 fish species using a multi-species stock assessment model: www.fisheries.noaa.gov/resource/data/2018-climate-enhanced-multi-species-stock-assessment-walleye-pollock-pacific-cod-and
a) walleye pollock (Gadus chalcogrammus)
b) Pacific cod (G. microcephalus)
c) arrowtooth flounder (Atheresthes stomias)

Social-economic management strategies:
a) no-fishing
b) harvest = acceptable biological catch determined from the assessment model and with a sloping harvest control rule
c) as in (b) but also with the social-economic modeled effects of the (status quo) EBM 2 million ton cap on annual groundfish harvest

Sampling strategy

Details of projection realizations that composed the ensemble members see Hermann et al. 2019 (https://academic.oup.com/icesjms/article/76/5/1280/5477847) and Hollowed et al. 2020 (uploaded and also available at https://search.proquest.com/openview/fa178eb91b9aced054ce510bdde90bdb/1?pq-origsite=gscholar&cbl=2049538).

The CMIP3 suite was selected during the BSP to span a broad range of potential sea ice dynamics (Hermann et al., 2016). Selected models from the CMIP5 suite included: the Geophysical Fluid Dynamics Laboratory (GFDL) – ESM 2M (ESM2M) (Dunne et al., 2012); the National Center for Atmospheric Research (NCAR) Community Earth Systems Model (CESM) (Kay et al., 2015); and the MIROC ESM (Watanabe et al., 2011) (Table 1). These three models were selected because they projected a broad range of global patterns for precipitation and SST, and provided contrasting views of future ocean conditions in the EBS. Output from these models under two RCPs (4.5 and 8.5; Van Vuuren et al., 2011; Van Vuuren and Carter, 2014) were used to drive the Bering10K regional model. RCP 8.5 and 4.5 represent a high-baseline carbon emission scenario and a moderate mitigation scenario, respectively.

We used hindcasts (1979-2017) and 2006-2100 climate change projections from a high resolution ROMSNPZ model (couple regional oceanographic -nutrient-phytoplankton- zooplankton model; see Hermann et al. 2019) to evaluate the effect of climate change on the biomass three fishery species in the Bering sea in order to contrast the potential of Ecosystem Based Management measures to help species and fisheries adapt to various levels of climate change under low carbon mitigation ("high baseline"; RCP 8.5) and moderate carbon mitigation scenarios (RCP 4.5). For projections from 2006-2100, the high resolution ROMSNPZ model is forced with boundary conditions from Coupled Model Intercomparison Project Phase 5 global climate model projections of atmospheric and oceanic circulation under climate change. Structural differences among global climate models can result in divergent trajectories; as part of the interdiscipilinary Alaska Climate Integrated Modeling project, three global climate models from a subset of the CMIP5 esemble members that reasonably replicated ice dynamics in the Bering sea were selected for this study: 1) the Geophysical Fluid Dynamics Laboratory (GFDL) – ESM 2M (ESM2M) (Dunne et al., 2012); 2) the National Center for Atmospheric Research (NCAR) Community Earth Systems Model (CESM) (Kay et al., 2015); and 3) the MIROC ESM (Watanabe et al., 2011) [see Hollowed et al. 2020 and Hermann et al. 2019 for more details]. This resulted in a suite of 6 projections of Eastern Bering Sea conditions including bottom and surface temperature, summer "cold pool", and large zooplankton (key prey resource) abundance during spring and fall (critical

periods for juvenile pollock and cod survival). We additionally included a persistence scenario as our "null" climate-constant scenario (i.e., average of 2006-2016 conditions).

For this study we choose three data-rich focal species that vary in economic and ecological importance: 1) the largest fishery in the United States for walleye pollock (Gadus chalcogrammus; ~1.4 million T yr-1 and 1.34 billion $ first wholesale value in 2017), 2) Pacific cod (G. microcephalus), one of the most economically valuable groundfish fisheries in the USA and also an important predator of pollock, and 3) arrowtooth flounder, a warm-tolerant predator of pollock that has relatively minor economic value presently.

Finally, we evaluated the risk of 10%, 50%, and 80% decline in catch and biomass over the persistence scenario by modeling 1) projections without harvest (unfished spawning biomass), 2) projections where in each year harvest was set to the sustainable limit using current management approaches (sloping harvest control rule and harvest rate that results in 40% of unfished spawning biomass), and 3) as in #2 but also with the added effect of the status quo Ecosystem Based Management 2 million ton cap on total groundfish harvest (i.e., modeled through a social-economic model of management and fisher behavior). These approaches are detailed in the methods section.

Data collection

ROMSNPZ:
Boundary conditions were based on CMIP5 projections for the Geophysical Fluid Dynamics Laboratory (GFDL) – ESM 2M (ESM2M) (Dunne et al., 2012); the National Center for Atmospheric Research (NCAR) Community Earth Systems Model (CESM) (Kay et al., 2015); and the MIROC ESM (Watanabe et al., 2011) and available through the Asia-Pacific Data Resource Centre (APDRC) CMIP5 data portal repository: http://apdrc.soest.hawaii.edu/datadoc/cmip5.php.

Dynamically downscaled projections:
The Bering10K regional grid has approximately 10 km horizontal resolution, with ten vertical levels (see Hermann et al. 2016, 2019 for more detail). As stated in Hermann et al. 2019, fine-scale bathymetry is based on ETOPO5 and supplementary datasets as described in Danielson et al. (2011). At the edges of the model the boundary conditions are forced by global climate model states. The CMIP5 projections from global models were dynamically downscaled by Hermann et al. 2019 for the Bering Sea (resulting in approximately 100 fold increase in resolution and increased hindcast skill). In this approach the ROMS ocean model is forced at the surface by freshwater and heat fluxes and wind-stress values derived from atmospheric states of the global model projections and by models states of sea surface temperature. Lateral boundary conditions were forced with conditions from oceanographic component of the global model projections (see Hermann et al. 2019 and Hollowed et al. 2020 for a list of variables and details about methodology).

CEATTLE Multispecies Assessment Model & ATTACH socioeconomic harvest model:
Biomass, age composition, length composition, diet/stomach contents, and weight at age data for the multi-species stock assessment model were provided by the summer groundfish survey conducted annually by the National Oceanic and Atmospheric Administration Alaska Fisheries Science Center https://www.fisheries.noaa.gov/alaska/commercial-fishing/alaska-fisheries-science-center-interactive-data-maps). Data for each species is included in the "assessment_files" folder of the https://github.com/kholsman/EBM_Holsman_NatComm repository.

Catch data including total harvested biomass, age composition, and weight at age for each species was provided by AKFIN (http://www.akfin.org) and the observer program at AFSC and is also included in the "https://github.com/kholsman/EBM_Holsman_NatComm/assessment_scripts/CEATTLE/src/Data/dat_input_files/01_assessment_2018".

Annual ROMSNPZ indices:
From these high resolution bi-monthly projections annual indices were produced including summer survey replicated bottom temperature and cold pool area indices, as well as the density of large zooplankton in the fall and spring months across the survey area. These indices were further bias corrected (see methods section) and are available for viewing at https://kholsman.shinyapps.io/aclim and are also available in the folder "https://github.com/kholsman/EBM_Holsman_NatComm/assessment_scripts/CEATTLE/src/Data/dat_input_files/01_assessment_2018".

SocioEconomic model simulations:
The socioeconomic ATTACH model used data available from the catch observer database (AKFIN: www.akfin.org) and historical records for harvest setting by the North Pacific Fishery Management Council (https://www.npfmc.org). The R package ATTACHv1.6.0 code and documentation used in this study is available for download and can be found at: http://doi.org/10.5281/zenodo.3966545. Updated documentation and code for ATTACH can be found on the [attach github]("https://github.com/amandafaig/catchfunction").

Timing and spatial scale

The spatial scale of the ROMSNPZ model is a 10- Km horizontal grid that encompasses the entire Bering Sea Shelf and basin and portions of the Bering Strait and Gulf of Alaska in the North Pacific. The model has 10 layers that scale with water depth. The grid includes survey replicated sampling stations that match the location of the summer trawl survey conducted annually by the National Oceanic and Atmospheric Association Alaska Fisheries Science Center (AFSC). AFSC annual trawl survey biomass, age, length, and diet contents of fish species form the basis of the CEATTLE multispecies stock assessment. Hermann et al. 2016, 2019 found that survey replicated bottom temperatures correspond closely to observed bottom temperatures and so we used bottom temperatures from the ROMSNPZ as inputs into the temperature functions of the CEATTLE model that delineate growth and predation in both the estimation (hindcasts of the ROMSNPZ) and projections (under various climate scenarios). The ROMSNPZ hindcast period (1979-2017) was selected for this study to match the biological sampling period of the AFSC summer survey used in the CEATTLE multispecies stock assessment model. Projections were bias corrected and recentered using overlapping periods between the hindcast and projections (2006-2017) and resulting corrected projections were used from 2017-2100 for each RCP scenario X GCM

(with the exception of the CESM RCP4.5 model which only had global projections through 2085).

Data exclusions	No data were excluded from the study
Reproducibility	All figures and analyses should be reproducible from the provided code and data sources.
Randomization	This paper details model simulations under projections which are for the most part deterministic. However for Figure 3, 100 random draws from lognormal parameter distributions of recruitment with climate covariates (fall and spring zooplankton, bottom temperature, and cold pool area; all bias corrected and recentered according to the methods outlined in the paper) used to generated the 10th and 90th quantiles for figure 3.
Blinding	N/A this is model simulation.

Did the study involve field work? ☐ Yes ☒ No

Reporting for specific materials, systems and methods

We require information from authors about some types of materials, experimental systems and methods used in many studies. Here, indicate whether each material, system or method listed is relevant to your study. If you are not sure if a list item applies to your research, read the appropriate section before selecting a response.

Materials & experimental systems

n/a	Involved in the study
☒	☐ Antibodies
☒	☐ Eukaryotic cell lines
☒	☐ Palaeontology
☒	☐ Animals and other organisms
☒	☐ Human research participants
☒	☐ Clinical data

Methods

n/a	Involved in the study
☒	☐ ChIP-seq
☒	☐ Flow cytometry
☒	☐ MRI-based neuroimaging
